# Maize pollen carry bacteria that suppress a fungal pathogen that enters through the male gamete fertilization route

**DOI:** 10.3389/fpls.2023.1286199

**Published:** 2024-01-10

**Authors:** Anuja Shrestha, Victor Limay-Rios, Dylan J. L. Brettingham, Manish N. Raizada

**Affiliations:** ^1^ Department of Plant Agriculture, University of Guelph, Guelph, ON, Canada; ^2^ Department of Plant Agriculture, University of Guelph, Ridgetown, ON, Canada

**Keywords:** maize, pollen, microbiome, Gibberella ear rot, *Fusarium graminearum*, mycotoxin, style, silk

## Abstract

In flowering plants, after being released from pollen grains, the male gametes use the style channel to migrate towards the ovary where they fertilize awaiting eggs. Environmental pathogens exploit the style passage, resulting in diseased progeny seed. The belief is that pollen also transmits pathogens into the style. By contrast, we hypothesized that pollen carries beneficial microbes that suppress environmental pathogens on the style passage. No prior studies have reported pollen-associated bacterial functions in any plant species. Here, bacteria were cultured from maize (corn) pollen encompassing wild ancestors and farmer-selected landraces from across the Americas, grown in a common field in Canada for one season. In total, 298 bacterial isolates were cultured, spanning 45 genera, 103 species, and 88 OTUs, dominated by *Pantoea, Bacillus, Pseudomonas, Erwinia*, and *Microbacterium*. Full-length 16S DNA-based taxonomic profiling showed that 78% of bacterial taxa from the major wild ancestor of maize (Parviglumis teosinte) were present in at least one cultivated landrace. The species names of the bacterial isolates were used to search the pathogen literature systematically; this preliminary evidence predicted that the vast majority of the pollen-associated bacteria analyzed are not maize pathogens. The pollen-associated bacteria were tested *in vitro* against a style-invading *Fusarium* pathogen shown to cause Gibberella ear rot (GER): 14 isolates inhibited this pathogen. Genome mining showed that all the anti-*Fusarium* bacterial species encode *phzF*, associated with biosynthesis of the natural fungicide, phenazine. To mimic the male gamete migration route, three pollen-associated bacterial strains were sprayed onto styles (silks), followed by *Fusarium* inoculation; these bacteria reduced GER symptoms and mycotoxin accumulation in progeny seed. Confocal microscopy was used to search for direct evidence that pollen-associated bacteria can defend living silks against *Fusarium graminearum* (*Fg*); bacterial strain AS541 (*Kluyvera intermedia*), isolated from pollen of ancestral Parviglumis, was observed to colonize the susceptible style/silk entry points of *Fg* (silk epidermis, trichomes, wounds). Furthermore, on style/silk tissue, AS541 colonized/aggregated on *Fg* hyphae, and was associated with *Fg* hyphal breaks. These results suggest that pollen has the potential to carry bacteria that can defend the style/silk passage against an environmental pathogen – a novel observation.

## Introduction

In flowering plants, the male gametophyte (pollen) delivers sperm nuclei to the ovary to facilitate fertilization, initiating the process of seed development. To enable this fertilization, the ovary has a passage (style channel) to the external environment via which the male gametes migrate, after being released from pollen grains (Thompson and Raizada, 2018). Environmental pathogens, including pathogenic fungi, exploit this channel to enter the seed (Kapu and Cosgrove, 2010). Style-entering fungal pathogens cause billions of dollars of losses to farmers globally and endanger human and livestock health by depositing mycotoxins in the grain (Miller et al., 2007; Maiorano et al., 2009; Thompson and Raizada, 2018). Though many of these pathogens are airborne, they can also be hitchhikers on pollen, including viruses, bacteria, and some fungi (Card et al., 2007; Donati et al., 2018; Kim et al., 2018; Kim et al., 2021; Fetters et al., 2022). Ultimately, these pathogens reduce progeny seed fitness and endanger the genetic contribution of the male gametes.

However, the concept that the pollen microbiome is biased for pathogens seems completely contradictory to the process of evolutionary selection that should select against pollen/pollen tubes that harbor more pathogens than beneficial microbes. A hallmark of the evolutionary strategy of sexual organisms, including animals and plants, is that they produce large numbers of male gametes which compete with one another for limited numbers of eggs (Walsh and Charlesworth, 1992; Minnaar et al., 2019). Such gamete competition would presumably select for pollen that carries beneficial microbes. This competition between pollen grains could occur along the entire male gamete migration route within females, which includes pollen landing and germinating on stigma, pollen tube invasion between cells of receptive trichome hairs (Kroh et al., 1979), pollen tube growth through the style passageway, and finally ovule fertilization (Minnaar et al., 2019). Supporting this view, artificial selection on pollen (gametophyte generation) *in vitro* for resistance to fungal pathogens was shown to reduce the incidence of the corresponding diseases in the progeny, thus improving the fitness of the sporophyte generation (Shobha and Ravikumar, 2006; Purnachandra et al., 2018).

Further contradicting the prevailing view that pollen is biased towards pathogen transmission, prior studies have shown that seeds possess complex microbiomes (Fisher et al., 1992; Johnston-Monje and Raizada, 2011; Liu et al., 2012; Pal et al., 2022), of which several members can benefit their host (Truyens et al., 2015; Shehata et al., 2016b; Bodhankar et al., 2017; Shehata et al., 2017; Dumigan et al., 2021). This observation is relevant if seed-associated microbes, the founders of the plant microbiome, are inherited from pollen. Indeed, a recent study by Wu et al. (2022) reported that a specific *Bacillus* bacterial strain from maize pollen could be transmitted to progeny seed, based on bacterial whole genome sequencing. There have been no reports as to the functions of pollen-associated microbes in any plant species to the best of our knowledge, including no studies about the impact of pollen-associated microbes along the gamete migration route, particularly the style passage. In fact, there are only a few reports in the literature about pollen having microbiomes, mostly focusing on human allergies and bee impacts (Obersteiner et al., 2016; Zasloff, 2017; Manirajan et al., 2018; Oteros et al., 2018; McFrederick and Rehan, 2019). Sequencing technology limited the majority of these prior studies to the V4-V5 region of 16S rRNA which provides reliable taxonomic resolution only to the family level and misses important taxa due to primer bias (Greay et al., 2019; Abellan-Schneyder et al., 2021), making it difficult to assess the extent to which the pollen microbiome consists of pathogens versus mutualists/commensals.

Here, we hypothesized, first, that pollen carries primarily non-pathogenic bacteria. Second, we hypothesized that pollen-associated bacteria can actively defend the style passage against fungal pathogens including those that damage the resulting progeny seed. Finally, if truly beneficial to host plants, we hypothesized that these beneficial bacterial traits would have been subject to natural and farmer selection, and hence should be found in wild relatives and diverse cultivars of a crop species.

To test these hypotheses, as a model system, we used maize (*Zea mays* L., corn) and a Gibberella ear rot (GER)-causing *Fusarium* fungal pathogen which invades the style passage at fertilization. An important advantage of maize is that it is wind pollinated and sheds large numbers of pollen grains (10^11^ to 10^13^ per ha) for collection, each with a large surface area to carry microbes (80-125 µm diameter) (Hofmann et al., 2014). Maize is one of the world’s three most important grain crops (García-Lara and Serna-Saldivar, 2019) with extant wild relatives and a well-known history of evolution, domestication, migration, and human selection. Specifically, maize was domesticated in southwestern Mexico about 9000 years ago from wild tropical teosintes (primarily *Zea mays* ssp. *parviglumis*, with a minor contribution from *Zea mays* ssp. *mexicana*, (Matsuoka et al., 2002; Warburton et al., 2011; Prasanna, 2012; Bedoya et al., 2017). It was then migrated by farmers to different latitudes and altitudes, ultimately resulting in landraces adapted by distinct indigenous peoples to local environments and needs (Ruiz Corral et al., 2008; Vigouroux et al., 2008; Langner et al., 2019).

Within the genus *Fusarium*, the species *Fusarium graminearum* (*Fg*) and its relatives cause devastating economic impacts on farmers globally (Yang et al., 2013). *Fg* is commonly reported to be part of the *Fusarium graminearum* species complex, but re-classification has recently been proposed (Crous et al., 2021). *Fg* and its relatives cause GER disease primarily in temperate regions, but GER has also been reported in Central America, Mexico, and South America where maize was domesticated and diversified (van der Lee et al., 2015; Mendoza, 2016; Silva et al., 2017; Kelly and Ward, 2018; Machado et al., 2021). *Fg* primarily exists as airborne spores, but is also known to be carried on pollen (Naik and Busch, 1978; Miller et al., 2007; Kostić et al., 2019) and enters silks at the time of pollination (Sutton, 1982; Ali et al., 2005). A recent study by Arata et al. (2023) reported the presence of diverse *Fusarium* species in maize tassels, further suggesting that pollen could be a potential *Fusarium* inoculum source. *Fg* and related GER-associated species enter silks via the silk hairs (trichomes) or surface wounds, after which they migrate along silk channels to ovaries, leading to diseased grain (Ali et al., 2005; Miller et al., 2007; Thompson and Raizada, 2018). GER-associated *Fusarium* species also produce mycotoxins in the grain including deoxynivalenol (DON) and zearalenone (ZEA). DON suppresses host defense responses by inhibiting protein synthesis (McCormick et al., 2011), thereby helping *Fg* and related species to colonize the host plant (Mudge et al., 2006) which impacts the fitness of the resulting progeny. ZEA acts as a hormone that regulates fungal sexual reproduction (Wolf and Mirocha, 1973). *Fg*, other *Fusarium* species and their mycotoxins present in pollen were reported to disrupt pollen tube growth (Tejaswini, 2002; Kačániová et al., 2011; Kostić et al., 2019), thus directly affecting the genetic contribution of the male gamete.

In a prior study, open-pollinated maize silks were shown to have a complex microbiome, with a subset of bacterial taxa increasing in abundance after being challenged with *Fg* (Khalaf et al., 2021). Taxonomic assignment of the taxa was limited by short-read V4 16S sequencing, and it was unclear whether the bacterial taxa originated from pollen or silk maternal tissue.

The objectives of this study were as follows: (1) to collect pollen from a diversity of maize, including its wild ancestors and landraces spanning thousands of years of farmer selection across the Americas; (2) to culture bacteria from the pollen, and then taxonomically classify them using full-length 16S rRNA sequencing; (3) to conduct literature searches to predict the fraction of the pollen-associated bacterial species that are likely pathogens; (4) to screen the bacteria *in vitro* for activity against a GER-associated *Fusarium* sp. and check for conservation of this trait across maize accessions; (5) to spray a GER-associated *Fusarium* sp. alongside the most potent bacterial strains onto silks in greenhouse trials, and measure disease suppression in the progeny seed; and (6) to undertake confocal fluorescent imaging for direct evidence that pollen-associated bacteria can defend living silks against *Fusarium graminearum*.

## Materials and methods

### Selection criteria and sources of maize accessions

**Figure 1:**
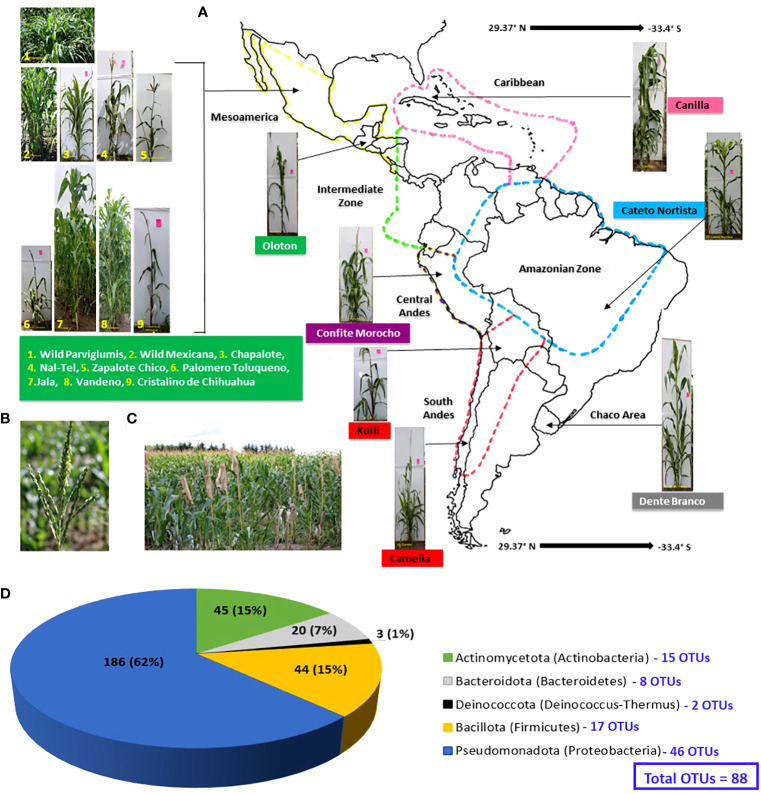
Origin of maize accessions used in this study to culture pollen-associated bacteria and their phylum and OTU level taxonomic distribution. Plants were grown in a common field in Canada. **(A)** A map of Latin America and the Caribbean showing the origin of the sixteen maize accessions used in this study. The map is adapted from a prior study (Bedoya et al., 2017). **(B)** Picture of a maize tassel shedding pollen. **(C)** Maize plants used in this study growing in a common field at Elora, Ontario, Canada, with tassel bags used to collect pollen. **(D)** Phylum and OTU level taxonomic distribution of the 298 bacterial isolates cultured from the pollen of the maize accessions in this study; the numbers inside the pie chart are the absolute number of isolates, while the brackets indicate their relative percentage in the collection. The numbers (in blue) corresponding to each phylum are the total number of OTUs belonging to that phylum (see **Supplementary Figure S1** for details).

A total of 16 accessions were included in this study: three were received from the U.S. Department of Agriculture (Germplasm Resources Information Network, GRIN) and 13 from the Maize Germplasm Bank of the International Maize and Wheat Improvement Center (CIMMYT) in Mexico. To maximize diversity, accessions were selected that originated from diverse latitudes, altitudes, and environmental conditions in the Americas. As noted below, accessions were also selected based on their fungal pathogen resistance, unique relevant traits and/or their historic importance to indigenous peoples (**Figure 1**; **Table 1**; **Supplementary Table S1**):

**Table 1:** Information about the origin of maize accessions selected in this study.

*Zea* accessions	Category	Seed Origin	Latitude	Longitude	Rainfall^^^	Altitude*	Accession ID
Cristalino de Chihuahua	Landrace	Mexico	29.37	-107.73	Dry	Highland	CIMMYTMA 7961^2^
Chapalote	Landrace, Primitive	Mexico	24.86	-107.42	Moderate	Lowland	NSL 2833^1^
Jala	Landrace	Mexico	21.09	-104.43	Moderate	Mid-altitude	CIMMYTMA 2246^2^
Canilla	Landrace	Cuba	20.9	-76.25	Moderate	Lowland	CIMMYTMA 5393^2^
Nal-Tel	Landrace, Primitive	Mexico	20.25	-89.65	Dry	Lowland	CIMMYTMA 2357^2^
Palomero Toluqueno	Landrace, Primitive	Mexico	19.283	-99.667	Wet	Highland	CIMMYTMA 6756^2^
*Zea mays* ssp. *mexicana*	Minor wild ancestor	Mexico	19.16	-98.55	Moderate	Highland	PI 566680^1^
*Zea mays* ssp. *parviglumis*	Major wild ancestor	Mexico	18.24	-99.54	Dry	Lowland	Ames 21826^1^
Vandeno	Landrace	Mexico	17.52	-101.28	Moderate	Mid-altitude	CIMMYTMA 177^2^
Zapalote Chico	Landrace	Mexico	16.217	-93.889	Moderate	Lowland	CIMMYTMA 10473^2^
Oloton	Landrace	Guatemala	14.633	-90.517	Moderate	Highland	CIMMYTMA 2510^2^
Confite Morocho	Landrace, Primitive	Peru	-12.77	-75.03	Wet	Highland	CIMMYTMA 8381^2^
Cateto Nortista	Landrace	Brazil	-16.667	-49.255	Wet	Mid-altitude	CIMMYTMA 26373^2^
Kulli	Landrace, Primitive	Bolivia	-18.18	-65	Moderate	Highland	CIMMYTMA 14235^2^
Dente Branco	Landrace	Uruguay	-32.683	-58.133	Wet	Lowland	CIMMYTMA 6162^2^
Camelia	Landrace, Primitive	Chile	-33.45	-70.667	Dry	Lowland	CIMMYTMA 15218^2^

^1^ Denotes seeds obtained from U.S. Department of Agriculture (Germplasm Resources Information Network, GRIN) and ^2^ from CIMMYT, Mexico.

*Lowland = 0-700 m.a.s.l; Mid-altitude = 701-1600 m.a.s.l; Highland = >1601 m.a.s.l.

^Rainfall classification: Dry= 500-<800 mm; Moderate=>800-<1270 mm; and Wet=>1270 mm.

Additional information is in **Supplementary Table S1**.

First, accessions were selected that originated at the center of maize diversification in Mexico and Central America, specifically (**Figure 1**): the ancient Mexican Jala landrace, known to have the largest cobs in the world and hence long silks (Rice, 2007); Zapalote Chico, known for its contributions to food security across Latin America (Taba, 1995); Vandeno, a Mexican progenitor of many modern maize varieties (Bedoya et al., 2017) that was reported to be resistant to aflatoxin produced by *Aspergillus flavus* (Ortega-Beltran et al., 2014); Nal-Tel, a primitive popcorn that was the foundation of the diets of the ancient Mayan culture in the Yucatan (Wellhausen et al., 1952; Turrent and Serratos, 2004; Santillán-Fernández et al., 2021); Palomero Toluqueno, the ancient Mexican progenitor of modern highland landraces (Perez-Limó et al., 2022); and Oloton, a highly pathogen resistant (CONABIO, 2017) Guatemalan highland landrace that became the staple food crop for the Mixe indigenous people in the high mountains of Oaxaca in Mexico (Pskowski, 2019).

Next, accessions were selected from the north and south migration of maize by indigenous peoples (**Figure 1**), away from its diversification center. Northward, the landrace Chapalote was selected, since it was one of the first maize landraces to enter the United States more than 2000 years ago (Da Fonseca et al., 2015) and has been continuously grown for 3000 years in Northern Mexico (Wellhausen et al., 1952; Bonavia, 2013). Cristalino de Chihuahua (Matsuoka et al., 2002) was also selected, as it is adapted to very dry environments (Ruiz Corral et al., 2008).

Landraces were also selected that were migrated by indigenous peoples across diverse South American landscapes, including (**Figure 1**): Confite Morocho, a primitive ancestor of maize from the Andes in central Peru, conversely known for its small ears (Grobman et al., 1961); the mid-altitude landrace Cateto Nortista from central Brazil (Bedoya et al., 2017); Kulli, a Bolivian highland landrace (Cuevas Montilla et al., 2011); Dente Branco, a lowland landrace from Uruguay that originated from the United States (Bedoya et al., 2017) and is resistant to *Fusarium* ear rot (USDA-ARS, 2018); and Camelia, another lowland landrace, but from Chile (Timothy et al., 1961) that is resistant to *Fusarium* ear rot (USDA-ARS, 2018).

Maize landraces also spread into the Caribbean, and from this region, the lowland landrace Canilla from Cuba was selected, grown by the pre-Columbian Taino peoples (Hatheway, 1957) (**Figure 1**).

### Plant growth conditions and experimental design for pollen collection

Since the maize accessions used in this study required a short day length between the V5 to V8 growth stages to induce eventual flowering, plants were initially grown in an indoor growth room with reduced day length until V8 before being transplanted to the field where the days were longer. Specifically, seeds were sown indoors in 5x5 inch biodegradable pots filled with a mixture of Sunshine Mix (LA4, Sungrow®Horticulture, Brantford, Canada) and living field soil from the Elora Research Station, Elora, Ontario, Canada (latitude: 43°41’ 3.59” N; longitude: -80° 25’ 22.79” W), as a potential source of microbes for the plant microbiome. Pots were arranged in trays in an indoor growth room with a 14 h photoperiod until the seedlings reached the V5 growth stage; the photoperiod was then reduced to 10 h until the V8 growth stage. Irrigation and fertilization (20:20:20 fertilizer with micronutrients) (Plant-Prod 20-20-20 Classic, #10529, Brampton, Canada) was done manually. Fluorescent light (LED 18 ET9/4/850 bulbs, GE) was used, supplemented with LED 9.5 A19/DIM/0/827/G4 1100 Lumen 2700K bulbs, Osram). The light intensity was 425-515 μmol/m^2^/s at pot level. Trays were rotated twice per week. At the V8 growth stage, a total of 780 large maize seedlings were transported to the field. The seedlings were loaded onto a trailer and left in the field for 3 days (2-3 h/day under direct sunlight and the remaining time under shade). After 3 days, the seedlings were transplanted to the field (July 10, 2019) at the Elora Research Station in a randomized block design with five replicate blocks, with six plants per block. The following treatments were applied: pre-transplant fertilizers (160 kg/ha of N, 60 kg/ha of P_2_O_5_, 80 kg/ha of K_2_O, and 10 kg/ha of S) as well as herbicides [4.0 L/ha of Primextra®II Magnum (Registration# 25730, Syngenta Canada) and 0.3 L/ha of Callisto® (Registration#100-1359, Syngenta, US)]. Manual irrigation using watering cans was done every day for one week after seedling transplantation, and thereafter the plants were rainfed.

### Pollen harvesting and bacterial culturing

Pollen harvesting occurred from August 13 to October 15, 2019. Once the maize started to shed pollen, tassels were bagged during the afternoons, and the pollen bags were collected the next morning. On average, a single tassel was bagged 5 times. In total, approximately 2500+ pollen bags were collected. The bags were brought to the lab and sorted to remove anthers and other debris. Each pollen sample was pooled by block (3-5 plants per block) and then stored at -80°C by adding 40% sterile glycerol for later culturing. For each maize accession, the pollen samples from all five blocks were pooled together for culturing bacteria. The frozen pollen samples were thawed and ground using sterile mortar and pestles in 600 µL of sterile 0.05 M sodium phosphate buffer (14.425 mL of 1 M Na_2_HPO_4_, and 10.575 mL of 1 M NaH_2_PO_4_, in a final volume of 500 mL of autoclaved ddH_2_O) at pH 7, then serially diluted: full concentration, 1/10, 1/100, and 1/1000 dilutions. Subsequently, 200 µL of each dilution was plated onto 150 mm X 15 mm Petri plates containing 50 mL of Reasoner’s 2A (R2A) agar medium, pH 7.2 (#OXCM0906B, Fisher Scientific, Canada). The plates were incubated for 3 days at 30°C, at which point unique colonies were re-streaked onto new R2A agar plates. These plates were returned to the incubator for two more days for a total of five incubation days to capture slow-growing colonies. A single pure colony from each new R2A plate was then grown in a 3 mL liquid culture of LB (Luria-Bertani, composed of 10 g NaCl, 5 g yeast extract, and 10 g tryptone, per liter, adjusted to pH 7.2) and incubated at 30°C with shaking at 200 rpm for 2 days. Then, glycerol stocks were made by adding 400 µL of the bacterial liquid culture with 600 µL of 40% sterile glycerol followed by storage at -80°C.

### Taxonomic analysis

One mL of each bacterial liquid culture was simultaneously used for genomic DNA isolation using QIAamp DNA mini kits (Catalog #51306, Qiagen, USA), following the manufacturer’s protocol. The DNA samples were quantified using a Qubit v1.2 fluorometer (Catalog #Q32857, Molecular Probes, USA). This extracted DNA was used to amplify full-length 16S rRNA using PCR. A PCR master mix working solution was prepared by mixing 20 µL of GoTaq Green Master Mix (M712C, Promega, USA), 1 µL of 10 µM 27f primer (5’-AGAGTTTGATCMTGGCTCAG-3’), and 1 µL of 10 µM 1492r primer (5’-GGTTACCTTGTTACGACTT-3’). To this, 100 ng of bacterial genomic DNA and Molecular Grade water were added to a total final volume of 40 µL. The PCR amplification conditions were as follows: initial denaturation at 96°C for 3 min, followed by 35 amplification cycles (94°C for 30 sec, 48°C for 30 sec, 72°C for 90 sec), and a final extension at 72°C for 7 min, using a PTC200 Cycler (MJ Scientific, USA). The amplified PCR products were verified by gel electrophoresis. Liquid amplicon mixtures were purified using the GFX PCR DNA purification kit (Catalog #45001489, GE Healthcare, Canada) and re-quantified using a Qubit v1.2 fluorometer. These quantified samples were used for PCR cycle sequencing. Template DNA at 28 ng per 1 kb was added to the bottom of the wells of a 96-well PCR plate which was then placed on a heat block at 96°C for 10 min until the DNA was dried completely. A master mix was prepared by mixing 1 µL BigDye (v.3.1) terminator mix, 2 µL 5X SeqBuffer, 8 µL Molecular Grade water, and 1 µL of 10 pmol/µL of each primer [27f primer (5’-AGAGTTTGATCMTGGCTCAG-3’) and 1492r primer (5’-GGTTACCTTGTTACGACTT-3’)]; it was mixed well and then this Master Mix was added to the dried DNA samples (12 µL per well) on the same PCR plate. The PCR plate was sealed and vortexed thoroughly to resuspend the DNA with Master Mix, followed by brief centrifugation. The plate was thermocycled under the following conditions: initial denaturation at 96°C for 2 min, followed by 30 amplification cycles (96°C for 30 sec, 50°C for 15 sec, 60°C for 4 min), using a PTC200 Cycler (MJ Scientific, USA). The plate was then submitted for sequencing at the Guelph MCB Genomics Facility. All 16S DNA sequences received were first trimmed and aligned to construct contigs using BioEdit software (Hall, 2011). Only one 27F or 1492R sequence was used if contigs could not be constructed. Nucleotide BLAST searches of the sequences were performed against the 16S ribosomal RNA sequence (Bacteria and Archaea) database at NCBI, optimized for Blastn, to obtain taxonomic predictions. The highest percent identity matches, with the higher query coverage, were counted as the top matches. The query coverage percentage reported was greater than 95%.

### Operational taxonomic unit assignment and phylogenetic tree construction

To assign each strain to a unique OTU, 16S DNA sequences sharing the same genus were multi-aligned using the ClustalW multiple-sequence DNA alignment tool (Madeira et al., 2019). Mismatches within 10 bp of the 5’ and 3’ termini were not counted as differences. Mismatches of a nucleotide base to ‘N’ were also not counted as a difference. Every unique sequence was assigned a unique operational taxonomic unit (OTU) number/group. Based on these criteria, a few of the OTUs represent more than one species. From the sequences within an OTU group, the sequence with the longest high-quality sequence was selected to represent that specific OTU group for constructing a phylogenetic tree. A total of 88 OTUs representing the complete culturable pollen library were used to generate a maximum likelihood (ML) phylogenetic tree with bootstrapping of 900 replicates, using MEGA-X software (Kumar et al., 2018).

### Systematic literature searches for evidence that the pollen-associated bacteria are known maize pathogens

Systematic literature searches were conducted as preliminary evidence to determine whether the cultured pollen-associated bacterial strains are known maize pathogens. The names of each bacterial species, along with the names of crops within the *Gramineae* family, were used as search terms in Web of Science and Google Scholar. The searches were restricted to pollen-associated bacterial species that were cultured in at least two maize accessions (**Supplementary Figure S3**).

### *In vitro* screening for anti-*Fusarium* activity

Dual culture assays were conducted to screen pollen-associated bacteria for anti-*Fusarium* activity *in vitro*, by adapting a prior protocol (Mousa et al., 2015). The GER-causing *Fusarium* strain used was *Fg*MT#1 (Genbank Accession OR730875), previously isolated from GER-diseased maize grain from Southwestern Ontario, Canada (Thompson, 2023). *Fusarium* mycelium was added to potato dextrose agar media, then poured into Petri dishes, into which wells were punched. Pollen-associated bacterial cultures in LB were added individually into the wells, incubated at 25°C for 48 h, and then the diameter of each *Fusarium* inhibition zone was measured. Each pollen-associated bacterium was screened independently in triplicate, then analyzed using One-Way ANOVA, and means were compared using Tukey’s pairwise comparison. See **Supplementary Methods** for details.

### Whole genome sequencing and genome mining

Anti-*Fusarium* bacterial isolates from the dual culture assays were sequenced and annotated at the Microbial Genome Sequencing Center (MiGS, Pittsburgh, USA). See **Supplementary Methods** for details.

### Suppression of GER-causing *Fusarium* in greenhouse trials

#### Selection of anti-*Fusarium* bacterial strains for greenhouse testing

Two criteria were used to select pollen-associated bacterial strains for greenhouse trials: potent anti-*Fusarium* activity in the dual culture assays (see above) along with susceptibility to common clinical antibiotics to ensure human safety in case of infection. Candidate anti-*Fusarium* bacterial strains were screened in triplicate against 20 antibiotics (see **Supplementary Methods** for details).

#### Plant growth conditions and experimental design

Surface-sterilized seeds of a moderately susceptible commercial maize hybrid DKC55-05RIB (Bayer Crop Science, Canada) were grown in the Guelph Crop Science Greenhouse Facility on May 25, 2021 (Trial 1) and June 11, 2021 (Trial 2) as described (See **Supplementary Methods** for details). There were a total of 168 maize pots arranged in a randomized block design. There were seven treatments (three pollen-associated bacterial candidates, a positive and negative control, as well as two non-pollen-associated bacterial candidates to be reported in a separate manuscript), arranged in 6 blocks, with each block having 4 treatment replicates. One replicate was considered to be a single plant in a single pot. A second completely independent trial was undertaken two weeks after the first one in a separate greenhouse room. Only the upper ear of each plant (the primary ear) was pollinated, treated, and scored.

#### First pollen-associated bacteria silk spray treatment

Each pollen-associated bacterium was cultured in LB liquid broth for 2 days at 30°C with shaking at 200 rpm. The bacterial liquid cultures were then centrifuged for 10 min, followed by resuspension in LB liquid media to an OD_600_ of 0.4-0.6. One mL of each bacterium was sprayed onto the silks 48 h after pollination. The air coolers in the zones were turned off each day from 4:30 pm (the time before starting the spray) to 6:00 am, to reduce airflow to avoid cross-contamination of the sprays.

#### *Fusarium* pathogen introduction

*Fusarium* isolate *Fg*MT#1 spore broth contained 0.35 g KNO_3_, 0.35 g KH_2_PO_4_, 0.175 g MgSO_4_, 0.175 g KCL, and 0.175 g dextrose, added to 175 mL of distilled water in a 1 L Erlenmeyer flask. In addition, 175 µL of micronutrient solution (20 mg/100 mL of each minor element: FeCl_2_, MnSO_4_, ZnSO_4_) was added, then the top of the flask was covered with aluminum foil and autoclaved. The solution was then allowed to cool at room temperature. Subsequently, two PDA plugs containing *Fg*MT#1 were added to each flask which was then covered with aluminum foil and placed in a large, dark-shaking incubator, and incubated for 2 weeks at 25°C and 120 rpm. The solution was then filtered using a cheesecloth and stored in the fridge. On the day of pathogen inoculation, the solution was standardized to 20,000 spores/mL by using a haemocytometer. At 72 h after the first bacteria silk spray treatment application, 1 mL of this *Fusarium* spore suspension was applied onto the silks that were marked/labeled as pollinated. The treated ears were covered with white plastic bags, which were tied to preserve humidity.

#### Second pollen-associated bacteria silk spray treatment

To ensure effective colonization of the bacteria, they were introduced a second time, 48 h after pathogen inoculation. The plastic bags of the ears were removed, then the silks were sprayed again with 1 mL of each bacterial treatment (prepared in the same way as the first application), and the bags were then restored. After a total of 10 days after the *Fusarium* treatments, the plastic bags were removed, and the ears were kept moist by misting water every afternoon for the next two weeks. The humidity of the greenhouse was kept high by spraying water onto the floors twice a day. In addition, each greenhouse zone was misted automatically every 2 min per hour (excluding 4 am to 10 am, to prevent pollination failure, because some reports suggest pollination is hampered if the humidity is high) (Mueller, 2017).

#### Control treatments

Proline fungicide was used as a positive control. A stock solution of Proline was prepared by adding 1.125% v/v (final concentration) of AGRAL®90 (registration# 11809, Syngenta Canada), a non-ionic wetting and spreading agent. Then, a single application of 1 mL of this solution was sprayed onto the silks 48 h after pollination, using spray bottles. For the negative control treatment, all the steps were identical except LB buffer was sprayed instead of bacterial cultures, as a true mock control.

#### Disease assessments

GER disease assessments were undertaken visually and blindly. The husks of the harvested ears were removed and each cob was phenotyped visually for the percentage of apparent infection, scored as the length of the diseased area from the cob tip (infection start site) relative to the total length of each respective cob. The disease was scored from 4 different sides per cob and then the average infected fraction of each cob was calculated, relative to its total length. The average grain weight was then calculated after shelling.

In addition, a visual disease severity assessment of individual kernels from each cob was undertaken blindly by a researcher who was not involved in the earlier disease assessments. Using cob pictures, kernels were individually scored only from one side/angle for each cob, representing half of the seed. A visual rating scale of 1-4 was developed for quantifying disease, as follows: 4 indicated the most infected [each kernel showed infection (i.e. mycelia) or damage (dark red color, shrunkenness) on all sides]; 3 indicated medium infection (limited to the top and sides of the kernel); 2 indicated low infection (either healthy kernel on the top portion but with damage on the sides or limited damage on the top portion with no damage on the sides); and 1 indicated no infection (no kernel damage and not covered with mycelium).

#### Quantification of GER-associated *Fusarium* mycotoxins

For *Fusarium* mycotoxin analysis, seeds were pooled from all cobs (4 cobs) from within each treatment in each block and mixed thoroughly; one-third of this mixture was used for mycotoxin analysis. All maize kernels from each sample were ground to a particle size of <850 µm using an M2 Stein mill (Fred Stein Lab, Inc. Atchinson, KS, USA). These ground samples were mixed and then a 10 g subsample was used for mycotoxin analysis. Measurements of grain DON and ZEA mycotoxins were conducted at Ridgetown Campus, the University of Guelph, using liquid chromatography-tandem mass spectrometry (LC-MS/MS) (see **Supplementary Methods** for details).

#### Statistical analyses

The percent diseased cob results, average grain weight, individual kernel disease severity and mycotoxins were modeled with a generalized linear mixed model (GLMM) using PROC GLIMMIX, then analyzed using One-Way ANOVA, where means were compared using Tukey’s pairwise comparisons except for the mycotoxin data for which means were compared using Dunnett’s comparisons in SAS version 9.4 (SAS Institute, Cary, NC) with a significance level of P ≤ 0.05.

### *Fusarium* vitality staining using light microscopy

To determine whether pollen-associated bacteria had direct fungicidal activity against *Fusarium* isolate *Fg*MT#1, *Fusarium* mycelia was placed in the center of each microscopic slide, along with candidate pollen bacterial culture to one side of *Fusarium*, and LB buffer to the opposite side as the negative control. After 24 h of incubation at 25˚C, the slides were stained with the vitality stain Evans blue (Catalog # E2129, Sigma Aldrich®, Missouri, USA) and imaged using a light microscope. There were 3 replicates for each bacterial candidate, with each slide placed in a separate Petri dish. See **Supplementary Methods** for details.

### Bacterial fluorescent tagging and confocal scanning fluorescence microscopy

Competent AS541 bacteria were transformed with plasmid pSW002-PpsbA-DsRed-Express2 (Wilton et al., 2018) and selected on LB agar containing tetracycline (5 mg/mL), incubated at 30°C for 24 h and screened for fluorescence. Seeds of modern maize inbreds (LH82, PHRE1) were surface sterilized, germinated in the lab for 1 week, then transplanted into pots containing 100% Turface® clay at the Crop Science Greenhouse Facility, University of Guelph. Under laboratory conditions, a prior protocol from our lab was followed (Thompson and Raizada, 2023) where DsRed-tagged AS541 culture was applied to the silks at the tip of each cob, then 24 h later, GFP-tagged *Fusarium graminearum* [transgenic strain ZTE-2A, in a GZ3639 background (Miller et al., 2004; Guenther and Trail, 2005; Cuomo et al., 2007) was applied. Wounds were made on the silks to mimic insect damage. Cobs were incubated in the dark at 25°C for 48 h, then the silk tissues were then stained with propidium iodide (1 mg/mL) (Catalog# P4864-10ML, Sigma-Aldrich, USA) and imaged using a confocal laser scanning microscope (model TCS SP5, Leica Microsystems, Mannheim, Germany) at the Molecular and Cellular Imaging Facility, University of Guelph.

## Results

### Taxonomic overview of pollen-associated bacteria cultured from wild and ancient maize across the Americas after growth in a common field

**Figure 2:**
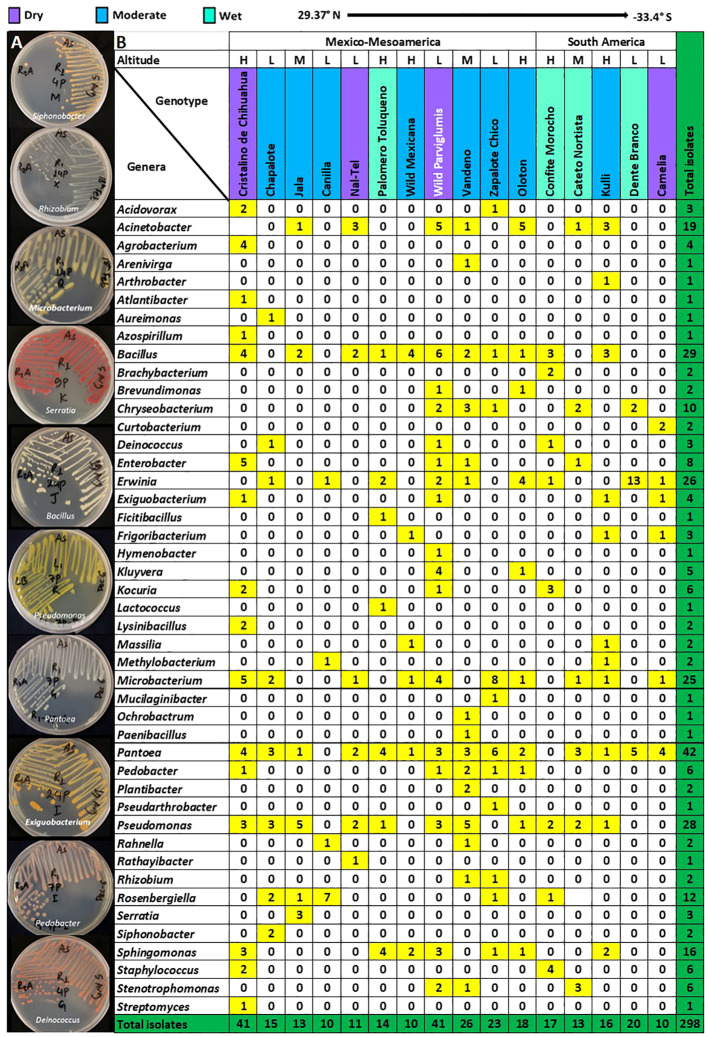
Predicted genus-level taxonomy of pollen-associated bacteria cultured from different host maize accessions spanning the Americas after growth in a common field. **(A)** Pictures of plates showing diverse bacterial genera after purification. **(B)** The distribution of the predicted bacterial genera across the examined host maize accessions. The predicted genera are based on full-length 16S Blastn searches at NCBI. The colors highlight the total number of isolates (including redundant and unique sequences) belonging to the corresponding bacterial genus, arranged by host maize accession. The data are organized by the latitude origin of source maize plants. The letter ‘H’ denotes a highland origin, ‘M’ denotes a mid-altitude origin, and ‘L’ denotes a lowland origin with respect to source maize plants. The rainfall classification is shown in different color codes (Dry, Moderate, and Wet). All plants were grown in a common field in Elora, Ontario, Canada.

After growth in a common field for one season, a total of 298 pollen-associated bacterial isolates were cultured from sixteen diverse American maize accessions including wild teosintes and landraces (**Figure 1**; **Table 1**; **Supplementary Table S1**) based on full-length 16S rRNA sequencing of the isolates cultured (**Supplementary Figure S1**; **Figure 2**). The sequences of these bacterial strains were deposited in Genbank and assigned accession numbers (**Supplementary Table S2**). The isolates belonged to five phyla, dominated by Pseudomonadota (Proteobacteria) with 186 isolates, followed by Actinomycetota (Actinobacteria) with 45 isolates, Bacillota (Firmicutes) with 44 isolates, Bacteroidota (Bacteroidetes) with 20 isolates and Deinococcota (Deinococcus-Thermus) with 3 isolates (**Figure 1D**). Within the phyla Actinomycetota, Deinococcota, and Bacillota, all bacterial isolates belonged to only one class whereas, within the remaining phyla, they belonged to more than one class. In total, the diverse American maize pollen-associated cultured library spanned 45 predicted bacterial genera, 103 predicted species, and 88 unique operational taxonomic units (OTUs) (**Supplementary Table S2**). Some of the OTUs represent more than one predicted species (**Supplementary Table S2**). Of the bacterial genera, 11 belonged to phylum Actinomycetota with 15 unique OTUs, 5 belonged to Bacteroidota with 8 OTUs, 1 belonged to Deinococcota with 2 OTUs, and 7 belonged to Bacillota with 17 OTUs; however, 21 belonged to Pseudomonadota with 46 OTUs, representing more than 50% of all pollen-associated OTUs (**Figure 1D**; **Supplementary Figures S1A, B**).

### Prevalence of culturable maize pollen microbiota across maize accessions

We first asked whether some pollen-associated bacterial taxa were prevalent across maize accessions. Within the phylum Pseudomonadota, the class Gammapseudomonadota was cultured from all 16 host maize accessions studied, belonging to eleven different genera (*Atlantibacter, Enterobacter, Kluyvera, Rahnella, Erwinia, Pantoea, Rosenbergiella, Acinetobacter, Pseudomonas, Stenotrophomonas*, and *Serratia*) (**Figure 2**). Within phylum Bacillota, Class Bacilli was isolated from 12/16 maize accessions, represented by seven genera (*Bacillus, Exiguobacterium, Ficitibacillus, Lysinibacillus, Paenibacillus, Staphylococcus*, and *Lactococcus*). Within phylum Actinomycetota, class Actinobacteria was retrieved from 12/16 maize accessions, represented by eleven genera (*Brachybacterium, Arenivirga, Curtobacterium, Frigoribacterium, Microbacterium, Plantibacter, Rathayibacter, Arthrobacter, Kocuria, Pseudarthrobacter*, and *Streptomyces*).

At the genus level, *Pantoea* was the most conserved in the pollen microbial library, accounting for ~14% (42/298) of the total isolates, and also the most prevalent, shared across 14/16 maize accessions (**Figure 2**; **Supplementary Figure S2**). The second most conserved genus was *Bacillus*, accounting for ~10% (29/298) of the total isolates, and shared across 11/16 maize accessions. *Pseudomonas* was the next highest cultured genus, accounting for 9.4% (28/298) of the total isolates, and recovered from 11/16 maize accessions. *Erwinia* was the fourth most cultured genus with ~9% (26/298) of the total isolates and shared across 9/16 maize accessions. The fifth most cultured genus was *Microbacterium*, accounting for 8.4% (25/298) of the total isolates, and retrieved from 10/16 maize accessions (**Figure 2**; **Supplementary Figure S2**).

**Figure 3:**
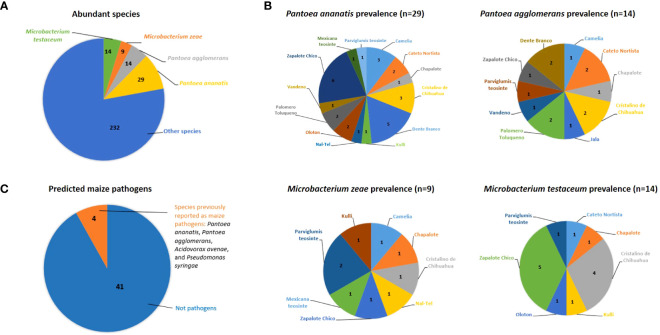
Abundant, prevalent and predicted pathogenic bacterial species cultured from the maize pollen library in this study. **(A)** The most abundant cultured bacterial species; the numbers shown are the number of isolates out of 298. The total number of bacterial species was 103 (See **Supplementary Table S2** for details). **(B)** Top 4 maize pollen-associated bacterial species in terms of their prevalence across maize accessions; the numbers shown are the number of isolates that were cultured from each maize accession (See **Supplementary Figure S3** and **Supplementary Table S2** for details). **(C)** Fraction of analyzed cultured pollen-associated bacterial species previously reported as maize pathogens. The numbers shown are the absolute number of isolates (See **Supplementary Table S4** for details).

At the predicted species level, *Pantoea ananatis* was the most prevalent (**Figure 3A**), shared between 13/16 maize accessions, followed by *P. agglomerans* (10/16 accessions), *Microbacterium zeae* (8/16 accessions), and *M. testaceum* (7/16 accessions) (**Figure 3B**; **Supplementary Table S2**).

Out of 88 OTUs, 45 were cultured from more than one host maize accession (**Supplementary Figure S3**). Of these shared OTUs, OTU16 (best match: *P. ananatis*) was the most prevalent, shared across 10/16 maize accessions (**Figure 3B**; **Supplementary Figure S3**). The next most prevalent OTUs, shared across 6/16 maize accessions, were OTU5 (best match: *Microbacterium zeae*), OTU8 (best match: *Erwinia aphidicola*), and OTU15 (best match: *M. testaceum*) (**Supplementary Figure S3**).

### Species and OTU level diversity within dominant pollen-associated bacterial genera

The genera that dominated the maize pollen bacterial library were found to have considerable species and OTU-level diversity (**Supplementary Figure S1**; **Supplementary Table S2**). The 42 *Pantoea* isolates encompassed 5 species (*P. ananatis*, *P. agglomerans*, *P. allii, P. anthophila*, and *P. brenneri*) and 7 unique OTUs (OTU7, OTU17, OTU69, OTU19, OTU42, OTU6, and OTU16). The 29 isolates of *Bacillus* spanned 13 diverse species and 8 unique OTUs. The 28 isolates of *Pseudomonas* included 11 species and 7 unique OTUs (**Supplementary Figure S1**; **Supplementary Table S2**).

### Taxonomic relationship of culturable pollen-associated microbes between the ancestors of maize and derived cultivated landraces

**Figure 4:**
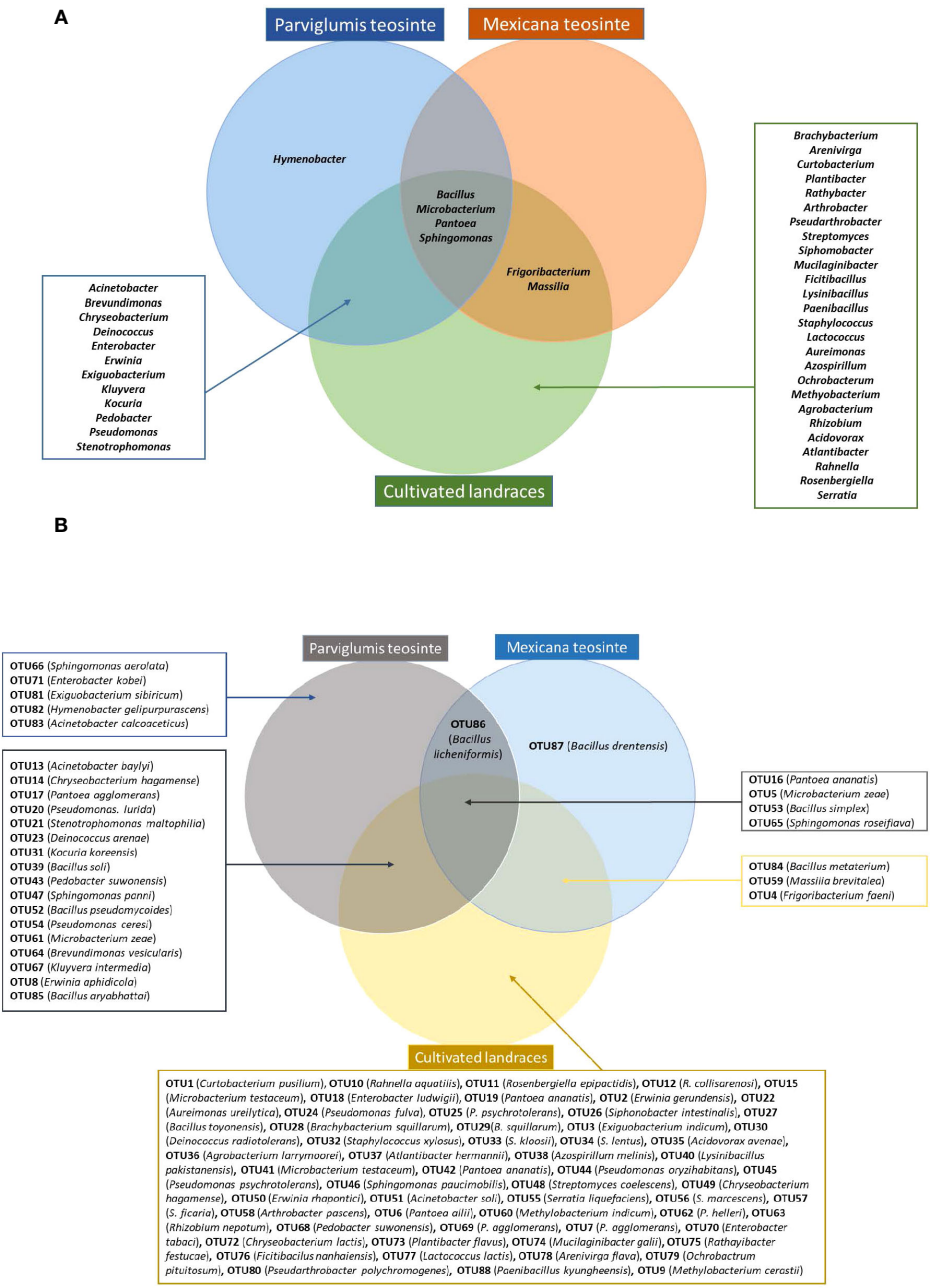
Venn diagram summarizing bacteria cultured from maize pollen of Parviglumis teosinte, Mexicana teosinte, and cultivated maize landraces that share predicted taxonomic identity based on 16S RNA sequences. **(A)** Bacteria sharing a genus-level taxonomy. **(B)** Bacteria sharing an OTU-level taxonomy.

The taxonomies of pollen-associated bacteria were compared between the wild progenitors, Parviglumis teosinte (*Z. mays* sp. *parviglumis*) and Mexicana teosinte (*Z. mays* sp. *mexicana*), and derived cultivated landraces after growth in a common field. Parviglumis was tied with the maize landrace Cristalino de Chihuahua in terms of the highest number of bacterial isolates from a host in this study, at 41/298 (~14%), spanning all 5 phyla noted above, 17/45 bacterial genera and 27/88 OTUs (**Supplementary Table S2**; **Figures 2**, **4**; **Supplementary Figure S2**). Of the 17 bacterial genera from Parviglumis pollen, 16 of them were also isolated from at least one cultivated landrace; similarly, 21 of its 27 OTUs (78%) were shared with pollen of at least one landrace (**Figure 4**).

Some bacteria present in Parviglumis pollen were not present in pollen of the cultivated landraces. At the genus level, *Hymenobacter* was only cultured from Parviglumis (**Figure 4**). Similarly, at the species level, *Acinetobacter calcoaceticus, B. licheniformis, B. soli*, *Enterobacter kobei*, *Exiguobacterium sibiricum*, *Hymenobacter gelipurpurascens*, *Microbacterium kyungheense*, *Pseudomonas cerasi, Sphingomonas aerolata*, and *S. yabuuchiae* were only retrieved from Parviglumis (**Supplementary Table S3**). At the OTU level: OTU66, OTU71, OTU81, OTU82, OTU83, and OTU86 were cultured from Parviglumis but were absent in all landraces (**Figures 2**, **4**; **Supplementary Table S3**).

By contrast, Mexicana teosinte, the minor ancestor of modern maize, yielded the lowest number of bacterial isolates in this study (10/298 or 3%), spanning only 6/45 bacterial genera and 9/88 OTUs (**Figures 2**, **4**; **Supplementary Table S2**). Mexicana pollen were the only source of 3 bacterial species: *Bacillus drentensis, B. loiseleuriae*, and *Massilia niabensis* (**Supplementary Table S3**). OTU87 (best match: *B. drentensis*) was unique to Mexicana. OTU86 (best match: *B. licheniformis*) was only found in Parviglumis and Mexicana (**Figure 4**; **Supplementary Figure S3**; **Supplementary Table S3**).

### Literature-based predictions of pathogenicity amongst maize pollen-associated bacterial species

Based on literature searches, out of 45 species cultured from maize pollen that were present in at least two maize accessions, only 4 species were previously reported as maize pathogens, specifically: *Pantoea agglomerans, Pantoea ananatis*, *Acidovorax avenae*, and *Pseudomonas syringae* (**Figure 3C**; **Supplementary Table S4**). Of these, only 6 OTUs spanning 3 species (*P. agglomerans, P. ananatis*, and *A. avenae*) were isolated from more than one maize accession (**Supplementary Figure S3**; **Supplementary Table S4**). By contrast, the majority of pollen-associated bacterial species present in at least two maize accessions were previously shown to possess diverse beneficial traits pertaining to abiotic and biotic stress tolerance in plants (**Supplementary Figure S3**; **Supplementary Tables S2**, **S4**).

### Pollen-associated microbiota uniquely cultured from individual maize landraces

In total, 21 out of the 45 cultured bacterial genera were each uniquely isolated from a single maize accession in the study panel (**Figure 2**; **Supplementary Table S2**). Within the dominant genera, there were also host-specific bacterial species and OTUs (**Supplementary Figure S3**; **Supplementary Tables S2**, **S3**).

### *In vitro* screening of pollen-associated bacteria for anti-*Fusarium* activity

**Figure 5:**
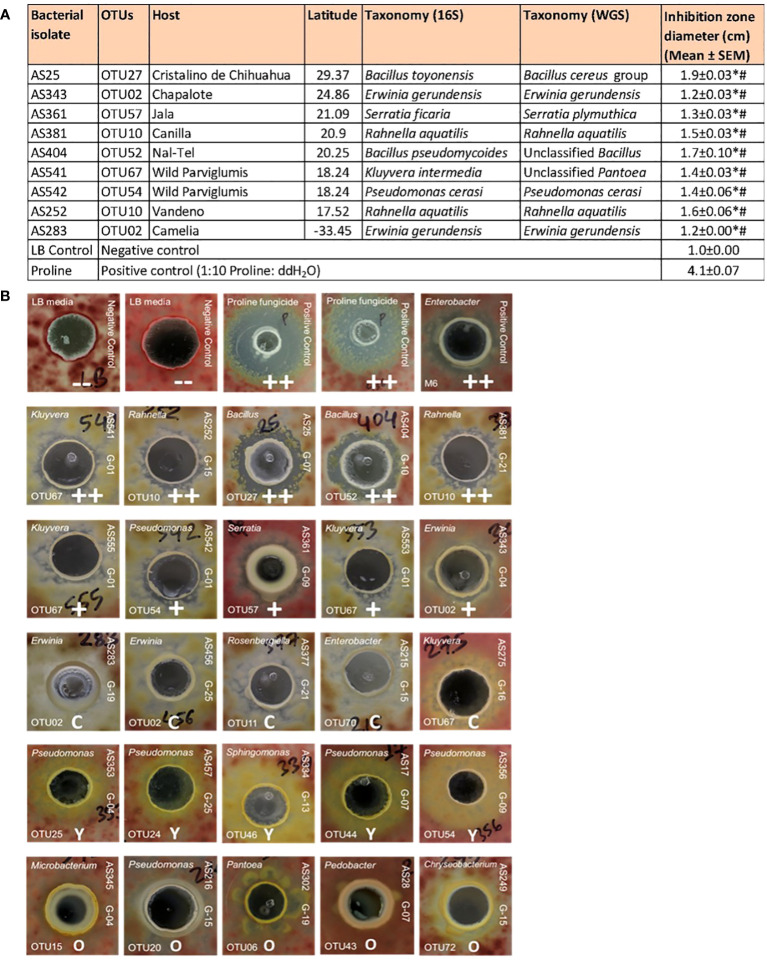
Testing pollen-associated bacteria for their ability to suppress GER-associated *Fusarium* isolate *Fg*MT#1 *in vitro*. **(A)** Summary of the bacterial isolates that showed anti-*Fusarium* activity along with their host source, 16S RNA and whole genome taxonomy, and their zone of inhibition against *Fusarium* (in cm) in dual culture assays. An asterisk (*) indicates that the zone of inhibition is significantly different from the LB buffer negative control; a number sign (^#^) indicates that the inhibition zone is significantly different from the Proline fungicide positive control (P ≤ 0.05, see Methods). **(B)** Results of the dual culture assays with some representative pictures. In each test, a single pollen-associated bacterial liquid culture or control solution was added into a well (center) in an agar plate embedded with *Fusarium* isolate *Fg*MT#1 (pink background). The rows on the top are the controls: LB buffer negative controls with no zones of inhibition (–), Proline fungicide positive control, and M6 positive control, which is an *Enterobacter* sp. previously shown to have anti-*Fg* activity (positive biological control). The pollen isolates in the second row have a strong zone of inhibition (++), third row isolates have minor inhibition zones (+), fourth row isolates have a clearing effect on *Fg*MT#1 without any organized zone of inhibition (C), fifth row isolates have a yellowing effect when interacting with *Fg*MT#1 (Y), and some sixth row isolates have an overgrowth effect (O). For each pollen isolate, the strain identifier information is noted: 16S BLAST genus prediction (top left corner), OTU number (bottom left corner), isolate sample identifier (ID) (top right side), and the source maize genotype ID (bottom right side). The host maize genotype IDs are as follows: G01, wild ancestral Parviglumis teosinte; G04, Chapalote; G07, Cristalino de Chihuahua; G09, Jala; G10, Nal-Tel; G13, Palomero Toluqueno; G15, Vandeno; G16, Oloton; G19, Camelia; G21, Canilla; and G25, Confite Morocho.

A total of 192 pollen-cultured bacterial isolates from diverse host maize accessions were screened for their ability to suppress the growth of a GER-associated *Fusarium* isolate (*Fg*MT#1) *in vitro* using dual culture assays (**Supplementary Table S2**). In total, 14 bacterial isolates reproducibly inhibited *Fusarium* growth (**Supplementary Table S2**). Among them, 9 inhibited *Fusarium* growth with strong zones of inhibition (**Figure 5A**); the inhibitory zones of these 9 strains were significantly different from the inhibitory zones of LB, the negative control, as well as Proline fungicide, the positive control (P ≤ 0.05). These anti-*Fusarium* strains belonged to diverse bacterial genera and were cultured from diverse host maize accessions (**Figures 5A, B**). AS361 (OTU57) was unique to the host Jala, whereas all the other anti-*Fusarium* strains were shared with multiple host accessions based on the 16S sequence. Strain AS25, belonging to a *Bacillus* OTU (OTU27, predicted to be *B. toyonensis*) resulted in the largest zone of inhibition (**Figure 5A**).

Of the 9 strong anti-*Fusarium* strains, AS252 and AS381 were the only isolates from the pollen library that belonged to the genus *Rahnella* (both OTU10, predicted to be *Rahnella aquatilis*, 99.36% identity) (**Figures 2**, **5A**; **Supplementary Figure S3**; **Supplementary Table S2**). They were cultured from two different maize host accessions, Vandeno and Canilla, respectively (**Figure 5A**). Similarly, anti-*Fusarium* strains AS283 and AS343 (**Figure 5A**) reported above, along with AS456 [(which showed a clearing effect without a measurable zone of inhibition (**Figure 5B**)], were the only 3 strains from the pollen library that belonged to OTU02 (*Erwinia*), cultured from maize hosts Camelia, Chapalote, and Confite Morocho (**Figure 5A**; **Supplementary Figure S3**; **Supplementary Table S2**).

In addition, some isolates displayed clearing effects without distinct zones of *Fusarium-*inhibition, or alternatively yellowing, or overgrowth effects (**Figure 5B**; **Supplementary Table S2**).

### Genome mining of pollen-associated anti-*Fusarium* bacteria

The genomes of the 9 strong anti-*Fusarium* bacteria, based on *in vitro* results, were mined for the presence of previously identified anti-*Fusarium* and/or anti-fungal genes (**Table 2**). The genomes of all anti-*Fusarium* strains encoded *phzF* (phenazine biosynthesis) and acetolactate decarboxylase (acetoin biosynthesis). Six strains encoded nitronate monooxygenase (NMO, nitro-oxidative damage mitigation), and four strains encoded butanediol dehydrogenase (2,3-butanediol biosynthesis). Other genes previously reported to have anti-*Fusarium* roles were not found (colicin V, surfactins, iturin, fengycin, and bacillomycin).

**Table 2:** Whole genome mining of pollen-associated anti-*Fusarium* bacterial strains to identify genes previously shown to contribute to biocontrol of *F. graminearum* and/or other fungal pathogens.

Bacterial strains	OTU	Taxonomy		Presence of Anti-*Fusarium*/Anti-Fungal Genes												
		16S	WGS	Phenazine biosynthesis (*phzF*)	Redox balance (Nitronate monooxygenase)	Acetoin biosynthesis (Acetolactate decarboxylase)	2,3-Butanediol biosynthesis (Butanediol dehydrogenase)	Chitosanase/chitinase	Surfactin synthetase (srfAA)	Colicin V biosynthesis	Iturin biosynthesis (ituAC)	Fusaric acid resistance (fusE)	Diacetylphloroglucinol biosynthesis (phlACBD)	Fengycin biosynthesis (fenC, fenD)	Bacillomycin D synthetase (bmyB)	Total
AS25	OTU27	*Bacillus toyonensis*	*Bacillus cereus group*													**5**
AS404	OTU52	*Bacillus pseudomycoides*	Unclassified *Bacillus*													**4**
AS541	OTU67	*Kluyvera intermedia*	Unclassified *Pantoea*													**3**
AS252	OTU10	*Rahnella aquatilis*	*Rahnella aquatilis*													**3**
AS283	OTU2	*Erwinia gerundensis*	*Erwinia gerundensis*													**3**
AS343	OTU2	*Erwinia gerundensis*	*Erwinia gerundensis*													**3**
AS361	OTU57	*Serratia ficaria*	*Serratia plymuthica*													**4**
AS381	OTU10	*Rahnella aquatilis*	*Rahnella aquatilis*													**3**
AS542	OTU54	*Pseudomonas cerasi*	*Pseudomonas cerasi*													**4**

The gray shading means “the presence of Anti-Fusarium/Anti-Fungal Genes”.

### Suppression of Gibberella ear rot in replicated greenhouse trials by pollen-associated bacteria

Three pollen-associated bacterial strains were selected for greenhouse trials based on the results of dual culture assays (**Figures 5A, B**), clinical antibiotic susceptibility testing (**Supplementary Figure S5**) and initial human Biosafety Risk Group 1 assignment, derived from 16S-based phylogenetic tree reconstruction (**Supplementary Figure S4**, see detailed figure legend). These were: AS25 (*Bacillus* OTU27, from Cristalino de Chihuahua), AS404 (*Bacillus* OTU52, from Nal-Tel), and AS541 (*Kluyvera* OTU67, from Wild Parviglumis) (**Figure 5A**).

**Figure 6:**
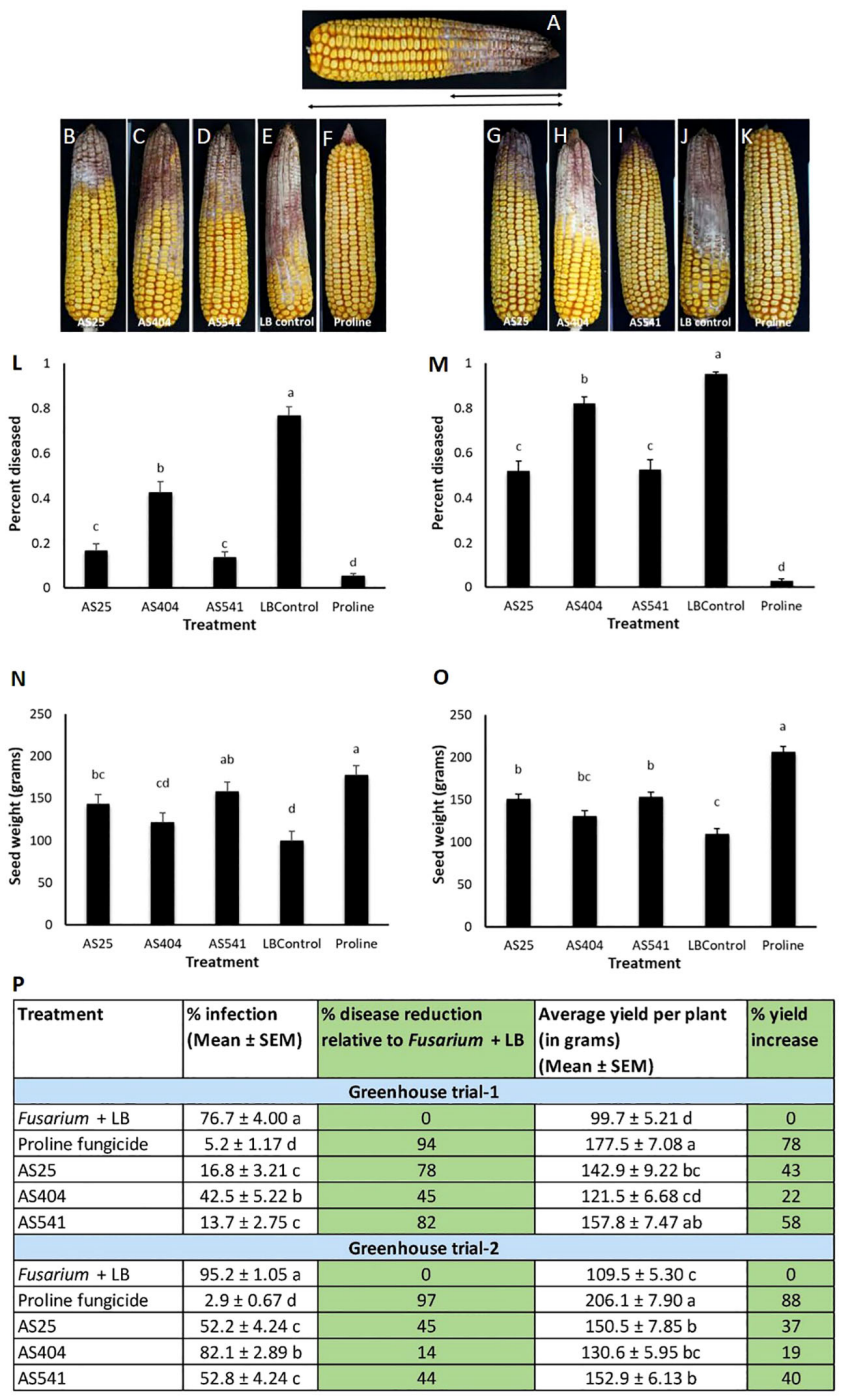
Greenhouse trials to test the ability of pollen-associated bacteria to suppress a GER-associated *Fusarium* species in modern hybrid maize. All silks were sprayed with a bacterial strain or a control solution before inoculation with *Fusarium* isolate *Fg*MT#1 and then again after (with the exception of Proline). **(A)** Picture of a mature cob illustrating the visual disease scoring method used which was quantified as the proportion of the diseased cob relative to the total length of the cob. The diseased portion was measured from tip to base (average of 4 measurements from 4 different sides of the cob), then multiplied by 100 to calculate the percentage of disease. **(B–F)** Representative treated cobs from each treatment in Trial 1. **(G–K)** Representative treated cobs from each treatment in Trial 2. **(L–O)** Quantification of the effects of different treatments on GER disease suppression: **(L, M)** percentage diseased ear in Trial 1 and Trial 2, respectively, and **(N, O)** average seed weight (in grams) in Trial 1 and Trial 2, respectively. **(P)** The effect of the bacterial strains on the percentage disease reduction and percentage yield increase relative to the *Fusarium* + LB buffer treatment (negative control). For both measurements, n=6 blocks per treatment, completely randomized (with 4 plants per treatment per block). Error bars indicate the standard error of the mean (SEM). The different letters on top of the histograms (panels L-0) and to the right of infection or yield measurements (in panel P) indicate that the mean values are significantly different from each other (P ≤ 0.05, see Methods).

The anti-*Fusarium* bacteria were sprayed onto silks prior to *Fusarium* inoculation, which resulted in significant reductions (P ≤ 0.05) in visual disease symptoms compared to the *Fusarium* + LB treatment, calculated by measuring the fraction of the cob length that was diseased (**Figures 6A, L, M, P**). Disease symptom reductions ranged from 45%-82% in Trial 1 and 14%-45% in Trial 2, with AS541 (from Wild Parviglumis) resulting in the highest disease suppression among the bacterial treatments. Proline fungicide resulted in the highest visual disease symptom reductions among all the treatments: 94% in Trial 1 and 97% in Trial 2. The three pollen-associated strains showed a similar trend of protecting the grain against yield loss in both trials. Compared to the *Fusarium* + LB treatment, the seed weight result was significant (P ≤ 0.05) for AS25 and AS541, but not AS404 (**Figures 6N, O**). Similar to visible disease symptom reduction, Proline fungicide resulted in the greatest protection against grain yield loss in both trials.

**Figure 7:**
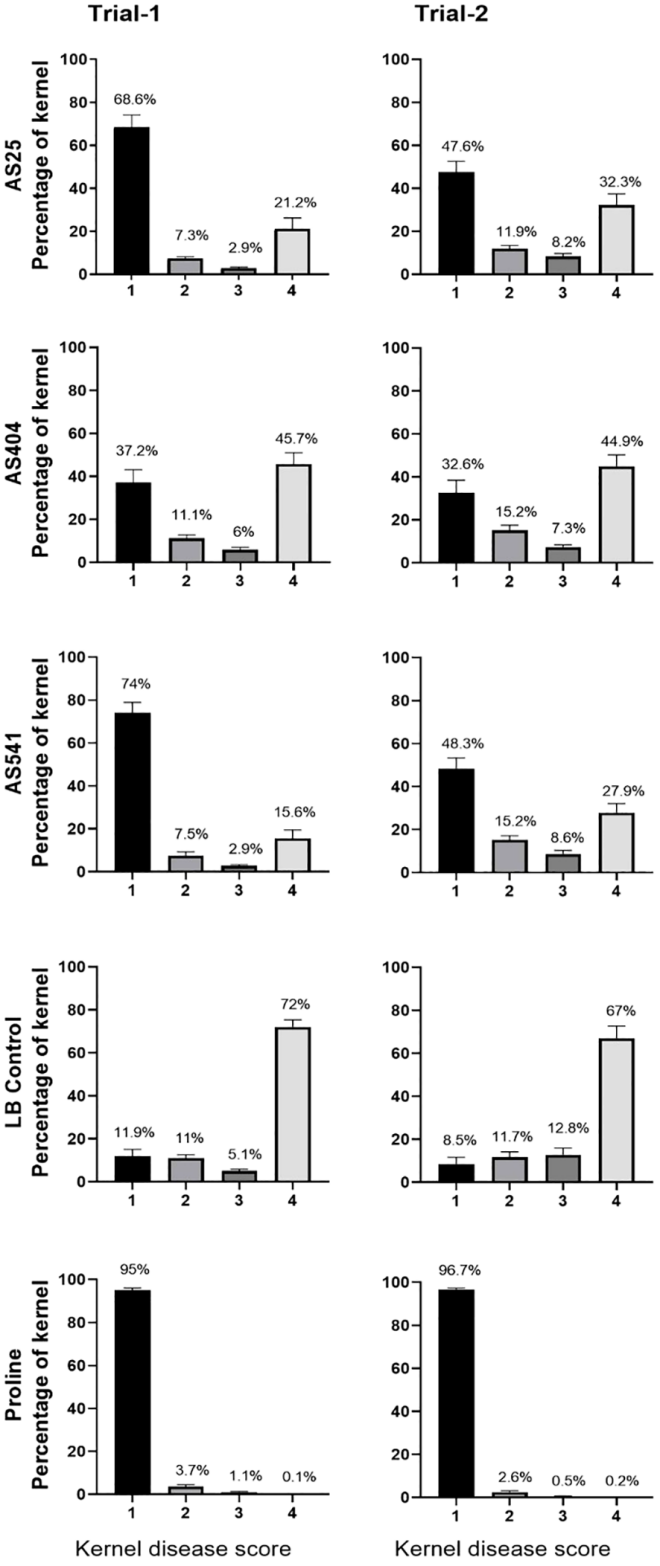
The ability of pollen-associated bacteria to suppress a GER-associated *Fusarium* species in modern hybrid maize based on visual disease severity assessment at the individual kernel level. All silks were sprayed with a bacterial strain or a control solution before inoculation with *Fusarium*, and then again after (except for Proline fungicide). The seeds were from greenhouse Trials 1 and 2. The scoring of kernel disease severity symptoms was done using a scale ranging from 1 to 4, with a value of 1 indicating no infection; 2 indicating low infection; 3 indicating medium infection; and 4 indicating highly infected. The percentages are the fraction of the kernel population with each disease value. Each percentage is the mean of 24 cobs (plants). Proline was the positive fungicide control; LB was the negative buffer control; AS541, AS404 and AS25 were the bacterial treatments used in the trials. All error bars indicate the standard error of the mean (SEM).

On an individual kernel basis, all three pollen-associated bacterial sprays resulted in a significant increase (P ≤ 0.05) in the percentage of seeds that had no visual disease symptoms (score of 1), compared to the *Fusarium*-only treated kernels (LB Control) (**Figure 7**; **Supplementary Tables S5A.3; 5A.4; 5B.3; 5B.4**), and a concurrent significant decline (P ≤ 0.05) in the percentage of seeds with severe disease (score of 4) (**Figure 7**; **Supplementary Tables S5A.1; 5A.2; 5B.1; 5B.2**). Of the three treatments, anti-*Fusarium* strain AS541 (*Kluyvera* OTU67, from wild Parviglumis) had the significant lowest percentage of diseased kernels (score of 4) in both trials (**Figure 7**; **Supplementary Table S5A.1; 5A.2; 5B.1; 5B.2**).

### Suppression of GER-associated grain mycotoxin accumulation by pollen-associated bacteria in replicated greenhouse trials

All three bacterial treatments showed similar trends of reducing GER-associated *Fusarium* mycotoxins (with the exception of DON-3-glucoside in Trial 2) by ~20-80% compared to the *Fusarium*-only treated plants (**Table 3**). Strain AS541 (*Kluyvera* OTU67, from wild Parviglumis) was generally the most potent across both trials; AS25 (*Bacillus* OTU27, from Cristalino de Chihuahua) was second in Trial 1, while AS404 (*Bacillus* OTU52, from Nal-Tel) was second in Trial 2 (**Table 3**).

**Table 3:** Reduction in mycotoxins in Gibberella ear rot (GER) diseased maize grain after treatment with pollen-associated anti-*Fusarium* strains.

Treatment	Trial 1		Trial 2	
	DON content (mean ± SEM)*	% DON reduction relative to *Fusarium* + LB	DON content (mean ± SEM)*	% DON reduction relative to *Fusarium* + LB
*Fusarium* + LB	222332.7 ± 26043.31	0	228889.9 ± 16449.41	0
Proline fungicide	2036.3 ± 246.98*	99	1084.3 ± 325.19*	100
AS25	110617.1 ± 44343.55*	50	150077.5 ± 23663.31*	34
AS404	154822.5 ± 38952.67	30	117391.9 ± 16457.99*	49
AS541	48445.4 ± 20831.08*	78	92820.4 ± 12060.09*	59
	3ADON content (mean ± SEM)*	% 3ADON reduction relative to *Fusarium* + LB	3ADON content (mean ± SEM)*	% 3ADON reduction relative to *Fusarium* + LB
*Fusarium* + LB	2845.6 ± 387.93	0	3015.1 ± 346.27	0
Proline fungicide	15.0 ± 2.58*	99	12.6 ± 3.71*	100
AS25	998.8 ± 421.20*	65	1614.2 ± 397.29*	46
AS404	1733.2 ± 415.88**	39	1449.9 ± 199.07*	52
AS541	465.9 ± 216.38*	84	932.4 ± 261.17*	69
	15ADON content (mean ± SEM)*	% 15ADON reduction relative to *Fusarium* + LB	15ADON content (mean ± SEM)*	% 15ADON reduction relative to *Fusarium* + LB
*Fusarium* + LB	1683.5 ± 392.82	0	1948.0 ± 105.21	0
Proline fungicide	5.5 ± 2.09*	100	4.5 ± 1.66*	100
AS25	969.6 ± 342.94	42	1484.2 ± 195.79	24
AS404	1317.2 ± 318.54	22	728.0 ± 189.70*	63
AS541	401.4 ± 133.08*	76	1304.1 ± 176.14*	33
	DON-3-glucoside content (mean ± SEM)*	% DON-3-glucoside reduction relative to *Fusarium* + LB	DON-3- glucoside content (mean ± SEM)*	% DON-3-glucoside reduction relative to *Fusarium* + LB
*Fusarium* + LB	29028.0 ± 6428.94	0	22650.0 ± 5070.14	0
Proline fungicide	28.8 ± 28.46*	100	35.0 ± 34.98*	100
AS25	15842.9 ± 5626.91	45	24441.1 ± 5828.14	-8
AS404	24200.5 ± 6233.34	17	19449.2 ± 7660.98	14
AS541	9194.2 ± 4249.57*	68	22345.9 ± 6634.02	1
	Zearalenone content (mean ± SEM)*	% Zearalenone reduction relative to *Fusarium* + LB	Zearalenone content (mean ± SEM)*	% Zearalenone reduction relative to *Fusarium* + LB
*Fusarium* + LB	47156.8 ± 11133.15	0	54430.4 ± 7406.29	0
Proline fungicide	211.9 ± 194.27*	100	14.8 ± 7.28*	100
AS25	26066.6 ± 14474.92	45	41417.0 ± 11919.59	24
AS404	34141.1 ± 11976.63	28	17317.6 ± 9631.88*	68
AS541	7778.3 ± 3996.59*	84	21771.1 ± 5297.94*	60

The seeds were from greenhouse Trials 1 and 2. Shown are the mean mycotoxin values (n=6 blocks, with 4 cobs pooled per block per treatment). All concentrations are in parts per billion (ppb). Asterisks indicate that the value is significantly different from the negative control (Fusarium + LB) (*=P ≤ 0.05; **= P ≤ 0.10).

### Light microscopy to determine whether pollen-associated bacteria exhibit direct fungicidal activity against a GER-associated *Fusarium* strain *in vitro*

**Figure 8:**
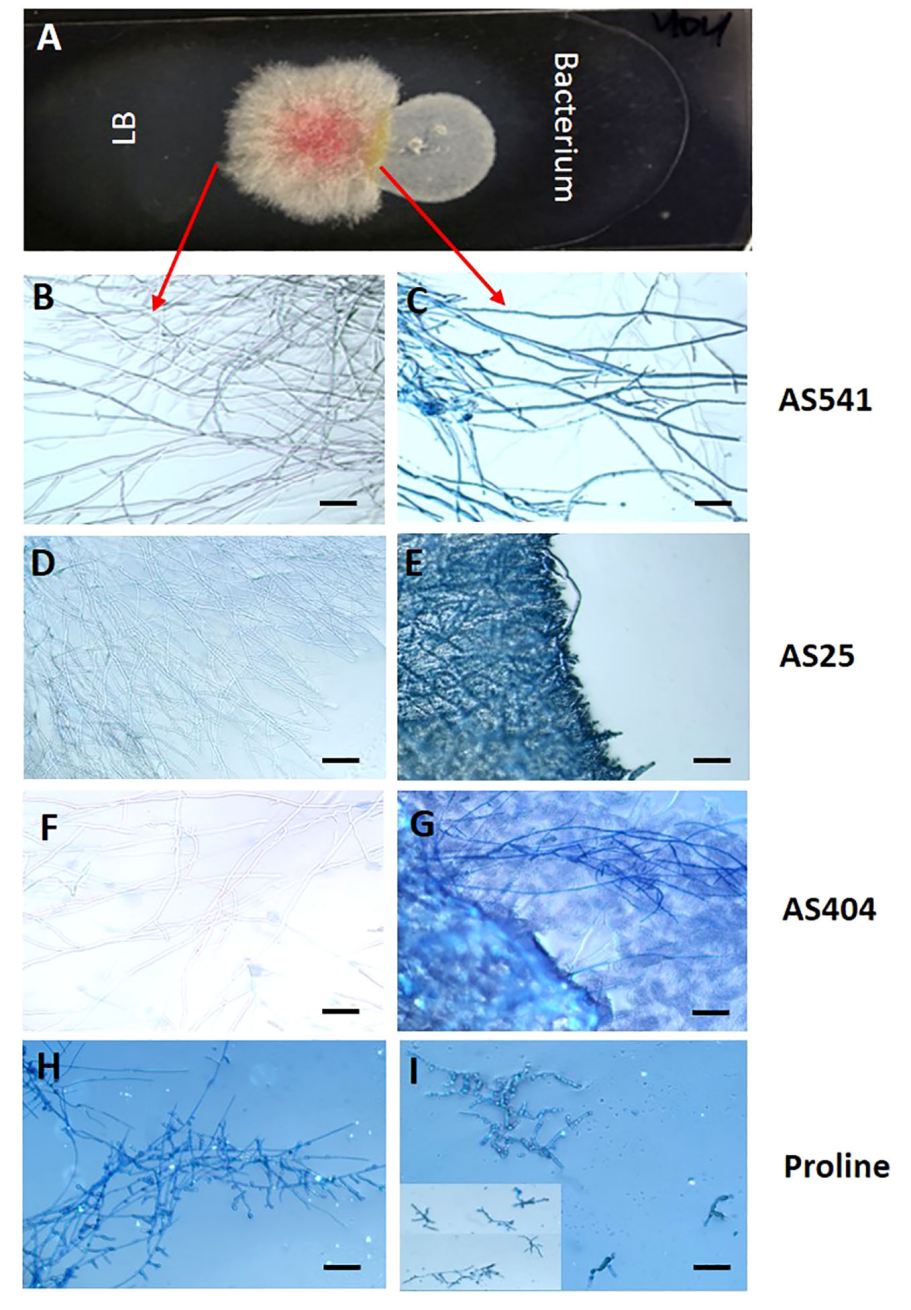
The interactions between a GER-associated *Fusarium* species and pollen-associated bacterial isolates after staining with the vitality stain Evans blue. **(A)** The methodology used: Microscope slides were coated with potato dextrose agar, then *Fusarium* isolate *Fg*MT#1 was inoculated into the center of each slide, then an anti-*Fusarium* bacterial strain or control on the right, and an LB liquid buffer control on the left. After co-incubation, Evan’s blue was added and then visualized using a light microscope: only dead *Fusarium* hyphae take up Evan’s blue. **(B, D, F, H)** *Fusarium* hyphae on the control side (no bacteria) and **(C, E, G, I)** the corresponding *Fusarium* hyphae from the side exposed to different treatments: **(C)** strain AS541, **(E)** strain AS25, **(G)** strain AS404, and **(I)** Proline fungicide. The inset in panel **(I)** is to display multiple hyphae. The scale bar in all images is 5 µM.

The earlier dual culture results showed that the 3 pollen-associated bacteria (AS25, AS404, AS541) tested in the greenhouse inhibited *Fusarium* growth. To test whether their activity was specifically fungistatic (i.e. competition) or fungicidal (i.e. direct killing), GER-associated *Fusarium* isolate *Fg*MT#1 and each bacterium were grown side by side on microscopic slides, then stained with Evans blue, which stains fungal mycelia blue when dead (i.e. indicative of a fungicidal mode of action). Microscopic analysis showed stained *Fusarium* hyphae blue after the application of all 3 anti-*Fusarium* bacterial strains, whereas unstained and healthy hyphae were observed in the non-bacterial controls (**Figure 8**). These results suggest that the 3 pollen-associated bacteria have fungicidal activity against a GER-causing *Fusarium* species.

### Live cell imaging of interactions between AS541 and *Fusarium graminearum* along the male gamete migration route (silks)

The earlier greenhouse trials showed that the anti-*Fusarium* bacterial strain AS541 could suppress Gibberella ear rot when sprayed onto silks, and furthermore that AS541 showed evidence of fungicidal activity *in vitro*. To understand whether AS541 protects the silk channels or only seeds, fluorescently tagged anti-*Fusarium* strain AS541 and *Fg* were co-applied onto intact silks from detached cobs and visualized using confocal scanning fluorescence microscopy. The anti-*Fusarium* AS541 bacterium was tracked using DsRed (here digitally transformed to a blue color), *Fg* using GFP (green color), and the silk cells were stained with propidium iodide (red color).

**Figure 9:**
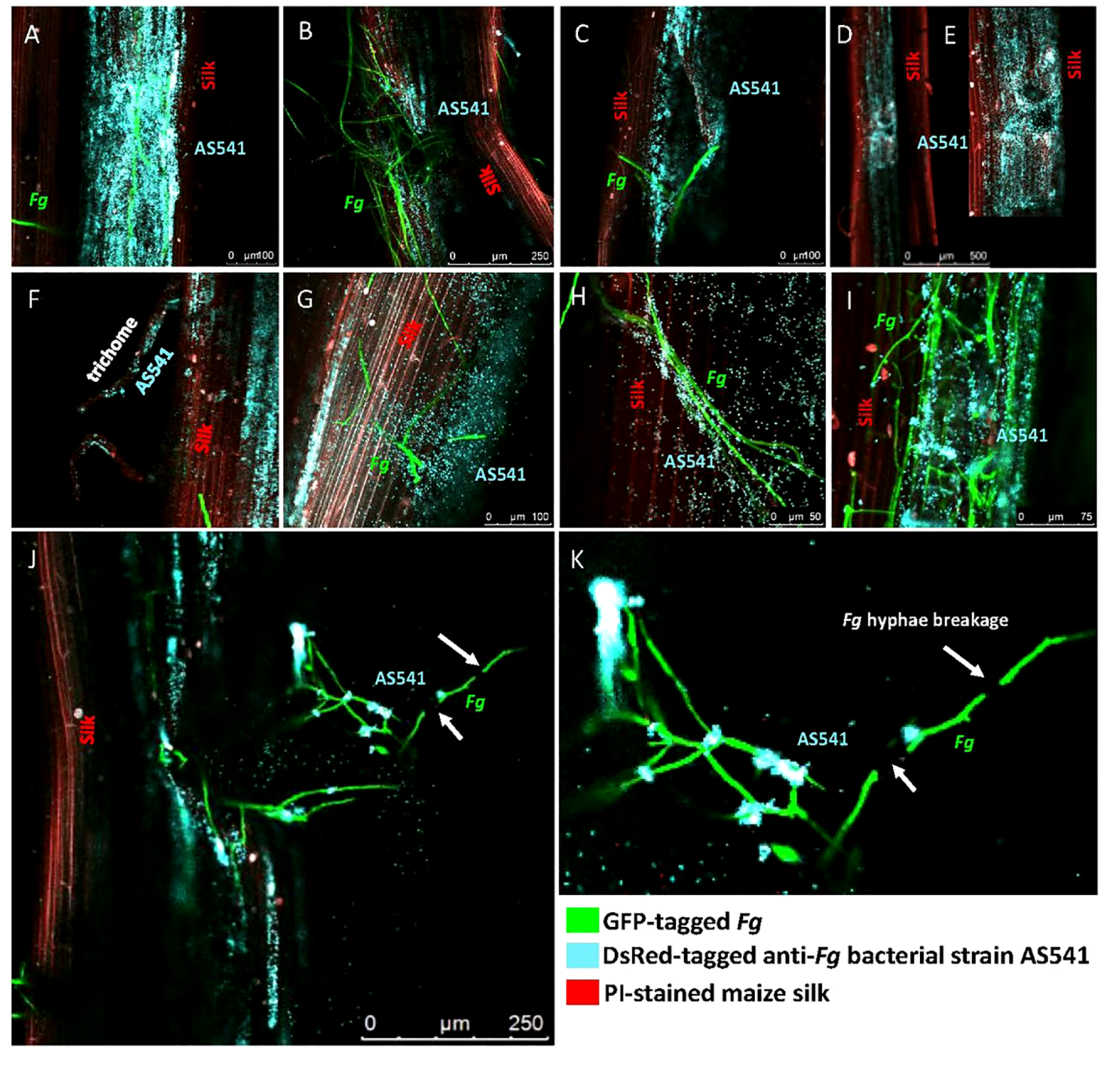
Confocal fluorescence microscopy imaging of maize silks inoculated with pollen-associated bacterial isolate AS541 with or without *Fusarium graminearum (Fg)* hyphae. AS541 was fluorescently tagged with DsRed, but false-colored blue, while *Fg* was tagged with a green fluorescent protein (GFP), and silks were stained with propidium iodide (red). In some experiments, silks were mechanically wounded to mimic insect damage, known to promote *Fg* colonization. **(A)** heavy colonization of AS541 on the silk surface around *Fg* hyphae; **(B)** AS541 colonization at a wound site associated with a significant presence of *Fg*; **(C)** AS541 colonization at a wound site with less presence of *Fg* hyphae; **(D, E)** AS541 colonization at wound sites in the absence of *Fg*; **(F)** AS541 colonizing a stigmatic trichome in the absence of *Fg*; **(G)** AS541 inhabiting a thick biofilm-like structure formed on the silk surface; **(H)** AS541 colonizing the surface of *Fg* hyphae; **(I)** AS541 encasing *Fg* hyphae on the silk surface; **(J, K)** Colonies of AS541 at or near the *Fg* breakpoints (white arrows).

In the presence of *Fg*, AS541 could sometimes be observed to heavily colonize the silk surface around *Fg* hyphae (**Figure 9A**). As *Fg* is known to enter through insect-mediated cob wounds, the silks were mechanically wounded with a needle: *Fg* was attracted to wound sites, but AS541 was also observed to colonize these sites, sometimes heavily (**Figure 9B**). At wound sites that were less masked by heavy *Fg* colonization, it appeared as if AS541 was attracted to the wound rather than *Fg* (**Figure 9C**). To test whether AS541 colonization around wound sites was stimulated by the presence of *Fg* or the wound itself, silks were wounded without *Fg* inoculation: the results showed that wounding alone was sufficient to promote colonization of AS541 (**Figures 9D, E**). *Fg* is also known to enter the silk channel via the trichomes; here, it was observed that AS541 could colonize trichomes, again even in the absence of *Fg* (**Figure 9F**). AS541 could also be observed to inhabit biofilm-like structures that protruded from the silk surface (**Figure 9G**, diffuse blue staining around bacteria on the right side). Taken together, these results suggest that AS541 could create presumptive barriers to *Fg* at known susceptible entry points associated with silks (silk epidermis, trichomes, and wounds).

In terms of targeted interactions of AS541 with *Fg* along the male gamete migration route, AS541 could be observed to colonize the surface of *Fg* hyphae (**Figure 9H**). In an independent trial, higher-resolution imaging revealed a biofilm-like structure containing AS541 cells encasing *Fg* hyphae on the silk surface (**Figure 9I**). In rare instances, breakage of *Fg* hyphae on surfaces of living silks could be observed associated with distinct colonies of AS541 at or near the *Fg* breakpoints (**Figures 9J, K**). Combined with the earlier Evan’s blue vitality staining, these results suggest that AS541 could aggregate and co-localize to *Fg* hyphae at locations associated with hyphae breaks on living silks.

## Discussion

During the process of fertilization, pollen reduces the survival of its future progeny by inadvertently carrying pathogens (Card et al., 2007; Donati et al., 2018; Kim et al., 2018; Kim et al., 2021; Fetters et al., 2022). Furthermore, the ovary becomes exposed to airborne pathogens including, in maize, Gibberella ear rot (GER) associated *Fusarium* species, because the ovary is connected to the style passage which, by necessity, is exposed to the environment to capture pollen; the style then transmits the male gametes to awaiting eggs (Kapu and Cosgrove, 2010; Thompson and Raizada, 2018; Khalaf et al., 2021). In the tropics and subtropics, other fungal pathogens also use the silk channel to infect the entire cob, including *Fusarium verticillioides, Aspergillus flavus, F. temperatum*, and *F. subglutinans* (Munkvold et al., 1997; Thompson and Raizada, 2018; Jabłońska et al., 2020). We hypothesized that maize pollen not only possess pathogens, but also carry beneficial anti-*Fusarium* bacteria to defend the style (silk) passage and resulting seed progeny. Pollen-associated bacteria have not previously been functionally analyzed in any plant species to the best of our knowledge.

Here, 298 bacterial isolates were cultured from pollen of diverse maize accessions, and bacterial taxonomy was assigned using full-length 16S RNA gene sequencing, which provided the highest-resolution examination (i.e. genus, species level) of a pollen microbiome compared to past studies which were limited by high-throughput sequencing technology limitations of the era (e.g. short reads) (Obersteiner et al., 2016; Manirajan et al., 2018; Oteros et al., 2018; McFrederick and Rehan, 2019). Our results showed that the culturable maize pollen microbiome possesses 5 phyla, 45 genera, 103 species, and 88 unique OTUs. In rank order, *Pantoea, Bacillus, Pseudomonas, Erwinia*, and *Microbacterium* genera were dominant and prevalent in pollen collected from diverse maize accessions (**Figure 2**; **Supplementary Figure S2**; **Supplementary Table S2**).

There were four major biological findings. First, consistent with our hypothesis, a systematic taxonomic comparison of the pollen-associated bacterial species to the pathogen literature identified only a small fraction of the bacteria as known maize pathogens (**Figure 3C**; **Supplementary Table S4**), but this result must be verified experimentally. Second, taxonomically diverse pollen-associated bacteria isolated from the major wild ancestor of maize and seven diverse landraces from Mexico, Chile and Cuba showed anti-*Fusarium* activity *in vitro* (**Figure 5**) and encoded *phzF* (**Table 2**), a key enzyme required for biosynthesis of the natural fungicide, phenazine (Mazurier et al., 2009; Pierson and Pierson, 2010). Third, greenhouse trials and confocal microscopy provided direct evidence that a subset of these bacteria could defend the style (silks) and/or the resulting seed progeny against anti-GER *Fusarium* species and their mycotoxins (**Figures 6**–**9**; **Table 3**). The pollen-associated anti-*Fusarium* strain used in confocal microscopy (AS541) was assigned to the species *Kluyvera intermedia* (based on 16S sequencing), which was recently shown by our group, in a parallel PacBio-based microbiome study (Khalaf et al., 2023), to be present in pollen of 15/17 diverse maize accessions. Finally, full-length 16S rRNA-based taxonomic profiling of pollen-associated bacteria showed that the majority of taxa from the major wild ancestor of modern maize were present in at least one post-domestication landrace in the study panel (**Figure 4**).

Combined, one possible interpretation of these results is that maize pollen-associated bacteria have been under long-term natural and human selection to protect their host plants. However, only a few pollen-associated bacteria were explicitly demonstrated here to be protective of their host plants. Furthermore, any interpretation of evolutionary selection should be viewed cautiously, as the pollen-associated bacteria in this study were cultured from plants grown for a single season, in a single field in Canada, not in their native habitats, and hence the bacteria may have been taken up from the common soil and environment of this study, rather than being vertically transmitted in plants – a pre-requisite for long-term selection. Future studies must be undertaken to determine the origin and extent of anti-pathogenic pollen-associated bacteria.

### A fraction of the maize pollen microbiome community can defend silks against GER-causing *Fusarium*

A previous study from our group (Khalaf et al., 2021) showed that the microbiome of pollinated maize silks changed in response to *Fusarium*, with multiple taxa increasing in abundance, but to the best of our knowledge this is the first functional study to demonstrate that pollen-associated microbes have anti-fungal traits. Here, in replicated greenhouse trials, three pollen-associated bacterial strains were sprayed onto maize silks prior to inoculation with a GER-causing *Fusarium* isolate, and shown to protect the resulting seed progeny against disease. The interactions between one strain (AS541) and *F. graminearum* (*Fg*) were also extensively studied on living silks using confocal microscopy.

All three tested pollen-associated bacterial strains [*Bacillus cereus* (AS25), *Bacillus pseudomycoides* (AS404), and *Kluyvera intermedia* (AS541)] suppressed disease symptoms and increased the percentage of seeds with no disease symptoms. Furthermore, *Fusarium* vitality staining in the presence of these pollen microbes suggested these strains have fungicidal activity against *Fusarium* strain *Fg*MT#1, further supported by the detailed confocal studies with AS541 and *Fg* using live silks. To the best of our knowledge, none of these bacterial strains was specifically reported to control GER disease in maize but was shown to suppress *Fg*, *Fusarium*, or other pathogens in other crops. It might be that previous researchers never attempted to spray these microbes on silks:

#### Bacillus cereus

*B. cereus* was previously reported to combat *Fusarium verticillioides in vitro*, in greenhouse and field trials in maize, where it reduced ear and root rots, reduced the accumulation of fumonisins, and increased grain yield (Martínez-Álvarez et al., 2016; Douriet-Gámez et al., 2018; Báez-Astorga et al., 2022). This observation is of interest, given that most maize accessions in this study originated from the tropics and subtropics where *F. verticillioides* is prevalent. *F. verticillioides* is pollen-philic and also uses pollen as a vector to reach the silk (Maiorano et al., 2009; Thompson and Raizada, 2018); it may be that farmers selected for *B. cereus* to combat *F. verticillioides* more than *F. graminearum* and related species. In this regard, prior studies have shown that *B. cereus* can successfully control *F. graminearum* seedling blight in wheat (Dal Bello et al., 2002) as well as *F. oxysporum* associated *Fusarium* wilt disease in tomatoes (Ramírez et al., 2022).

#### Bacillus pseudomycoides

*B. pseudomycoides* has been studied as a biocontrol agent in alfalfa against *F. graminearum*, *F. proliferatum*, and *F. oxysporum* (Knežević et al., 2021). It was also reported to control *F. oxysporum* and *Ralstonia syzigii* in chili pepper (Yanti et al., 2018), delay *Fusarium* infection in banana plants (Hadi et al., 2021), and antagonize *Fomes lamenensis* and *Ralstonia solani in vitro* (Vandana et al., 2018).

#### Kluyvera intermedia

*K. intermedia*, a member of the pan-American core maize pollen microbiome (Khalaf et al., 2023), was previously shown to antagonize the fungal pathogen *Rhizoctonia solani* (Anak et al., 2021), and the bacterial pathogen *Pseudomonas syringae* in kiwifruit (Tontou et al., 2016), but we could not find any reports from the literature of this species having anti-*Fg* or anti-*Fusarium* activities. It is also noteworthy that several studies reported that *K. intermedia* exhibited plant growth-promoting traits (Vazquez et al., 2000; Pallavi and Gupta, 2013; Boechat and Carlos, 2020; Pratiwi et al., 2020). In this study, we showed that *K. intermedia* (AS541) could colonize silks and create three types of presumptive barriers against *Fg* at known susceptible entry points associated with silks (silk epidermis, trichomes, and wounds). *Fg* spores germinate on silks, and hyphae spread to the surface and inside of the silks until they infect the ovules (Miller et al., 2007; Li et al., 2018). On the silk surface, AS541 appeared to form a biofilm, which is known to prevent fungal pathogen entry into plant tissues (Das et al., 2017; Muhammad et al., 2020). Biofilms are known to act as physical barriers (Stewart, 2002) and can also shield bacteria from antibiotics secreted by *Fg* as a counter-defense (Timmusk et al., 2019). Here, AS541 could also colonize silk trichomes, responsible for guiding gamete-delivering pollen tubes as well as fungal pathogens into the main silk channel during the initial stages of infection (Miller et al., 2007). Intriguingly, AS541 could colonize wound sites in the absence of *Fg*. After infection, on living silks, AS541 was observed to interact directly with *Fg* by colonizing and encasing hyphae and being associated with hyphal breaks, which might suggest that it is attracted to, and can kill, *Fg*. Combining the live silk imaging results and the potency of AS541 to suppress *GER* disease in greenhouse trials, we hypothesize that AS541 can prevent *Fg* and related species colonization, but once *Fg* colonizes, AS541 can kill it. Seek-and-kill behavior was reported for a root-inhabiting bacterial endophyte (*Enterobacter* strain M6) against *Fg* on the finger millet root surface (Mousa et al., 2016), as well as by *Burkholderia gladioli* against the fungal pathogen *Clarireedia jacksonii* (previously *Sclerotinia homoeocarpa*) *in vitro* (Shehata et al., 2016a). These results lend support to the hypothesis that immobile plant cells may have selected for mobile microbes to serve as an accessory defense system to target pathogens – a type of convergent evolution with circulating lymphocyte immunity cells in animals (Soliman et al., 2015; Talbot, 2015; Mousa et al., 2016). However, further studies are needed to determine whether AS541 is simply attracted to plant nutrients (especially at silk wound sites), and direct evidence is needed for chemo-attraction to *Fg* hyphae.

Nevertheless, these observations suggest that AS541 has multiple modes of action against *Fg* and lead to the hypothesis that there was a three-way co-evolution between AS541, maize (pollen, silks) and *Fusarium*. Future studies are needed to understand antibiosis modes of action, and the role of pollen tubes. It may be that after pollen lands on silks, pollen-associated microbes are transferred onto the silks directly, or via a pollen tube growing inside the silks. The last hypothesis is attractive, because *Fg* and other *Fusarium* species and their mycotoxins are present in pollen and disrupt pollen tube growth (Tejaswini, 2002; Kačániová et al., 2011; Kostić et al., 2019), thus creating selection pressure for microbes that could protect the pollen tube itself. Bacteria inside pollen tubes would presumably originate from inside pollen grains; a study by Manirajan et al. (2018) showed pollen contains bacteria on the pollen surface but whether bacteria also reside inside pollen remains unclear.

### Potential genetic mechanisms of anti-fungal activity

In terms of the genetic mechanisms underlying the fungicidal/*Fusarium* cleavage activities observed with the anti-*Fusarium* strains, the genomes of all 9 anti-*Fusarium* isolates contained the *phzF* gene, responsible for the biosynthesis of the natural fungicide, phenazine (Mazurier et al., 2009; Pierson and Pierson, 2010). Phenazine has been extensively explored for its application to plant disease management (Pierson and Pierson, 2010). Phenazines produced by some *Pseudomonas* species can suppress *Fg* mycelial growth (Zhou et al., 2016) and inhibit *Fg* spore germination, mycelial growth, and suppress DON mycotoxin production in wheat heads (Hu et al., 2014; Sun et al., 2021). Phenazine has been shown to disrupt the growth and virulence of *Fg* in part by disrupting histone acetyltransferases, leading to altered fungal gene expression (Chen et al., 2018). Additionally, phenazines antagonize *F. verticillioides* in maize (Diniz et al., 2022), and have been shown to inhibit the growth of both *F. verticillioides* and *Aspergillus flavus in vitro* (Palumbo et al., 2007), lending further support to the theory that these pollen-associated anti-*Fusarium* bacterial isolates might combat other silk-invading fungi across the Americas.

Some of the anti-*Fusarium* strains including *Bacillus* OTU27 also contained genes for the catalysis of chitin, a principle component of fungal cell walls (Mack et al., 2015). Chitinases hydrolyze the glycosidic and peptide bonds of chitin resulting in cell lysis, severe cell damage, and even pathogen death (Dimkić et al., 2022). The hydrolysis of chitin produces N-acetyl glucosamine, inducing resistance in plant hosts (Gohel et al., 2016). Bacterial chitinase activity has been reported to be the major mechanism for the biocontrol of diseases in some studies including in *Bacillus* species. For example, chitinase-producing *B. pumilis* (Dimkić et al., 2022), *B. subtilis* (Mahmoud, 2016), and *B. subtilis* SG6 (Zhao et al., 2014) have been recognized as biocontrol agents against *Fg* in wheat. Furthermore, a study by Vujanovic and Goh (2011) reported that chitinase and chitosanase-producing endophytic fungus *Sphaerodes mycoparasitica* absorbed and perhaps neutralized aurofusarin from attacked *Fusarium* cells after first lysing chitin; aurofusarin is a mycotoxin secreted by *Fg* (Vujanovic and Goh, 2011).

The extensive colonization of AS541 on silk trichomes and epidermis and around wound site surfaces in the absence of *Fg* could indicate direct signaling between pollen-associated bacteria and silk cells. In this context, it is noteworthy that the anti-*Fusarium* strains also encoded genes responsible for the biosynthesis of acetoin and butanediol, hormones known to trigger host Induced Systemic Resistance (ISR). These genes were found in several of the anti-*Fusarium* strains including *Bacillus* and *Pseudomonas*; AS541 encoded the acetoin biosynthetic gene. Prior studies have shown that acetoin-producing *Bacillus* and *Pseudomonas* can trigger ISR, including, for example, *Bacillus subtilis* in *Arabidopsis thaliana*, to protect against *Pseudomonas syringae* (Rudrappa et al., 2010), and *Bacillus velezensis* in tobacco (Peng et al., 2019). Acetoin is also reported to help trigger plant growth promotion (Ryu et al., 2004) as well as drought tolerance (Han et al., 2007), both of which may have relevance to silk growth and health. In Arabidopsis, 2,3-butanediol secreted by *Bacillus amyloliquefaciens* and *Bacillus subtilis* has been shown to elicit ISR (Ryu et al., 2004). In terms of silk colonization mechanisms, it is interesting to note that the genome of *K. intermedia* (AS541), here the most effective anti-*Fusarium* bacterial strain following silk spraying, encoded three Type III secretion system operons (**Supplementary Table S6**), while the least effective strain (AS404) encoded none, and the intermediate strain (AS25) encoded a single operon. In endophytes and epiphytes, Type III systems are known to facilitate the secretion of effectors that reduce host defenses, to promote stable colonization (Zboralski et al., 2022).

**Figure 10:**
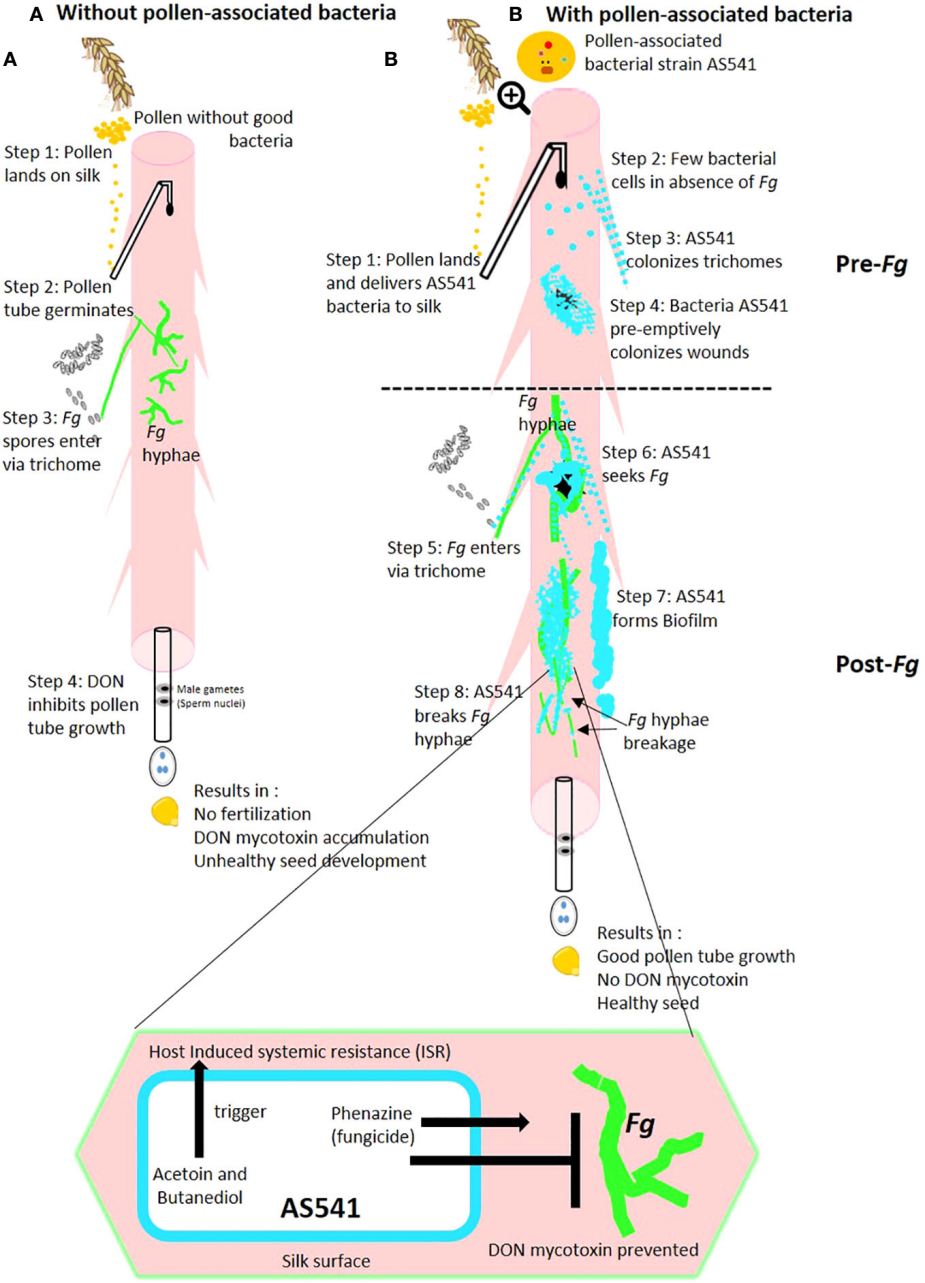
Hypothetical model of the mechanisms by which anti-*Fusarium* pollen-associated bacterial strain AS541 protects the male gamete migration route on maize silks in the: **(A)** absence of bacterial strain AS541; **(B)** presence of bacterial strain AS541. *Fg, Fusarium graminearum*; DON, deoxynivalenol.

These diverse, potential anti-*Fusarium* mechanisms encoded by pollen-associated bacteria shown by microscopy and/or predicted by their genome annotations are summarized in a hypothetical model (**Figure 10**) but need to be validated by additional experiments. Furthermore, the anti-fungal activities of pollen-associated bacteria may not be limited to silks or pollen tubes, but might be important in the pollen itself (gametophyte stage) or after fertilization in the seed or resulting progeny plants (sporophyte generation).

### Taxonomy-based predictions of pathogens versus mutualists amongst maize pollen-associated bacteria

As already noted, pollen is reported to transmit disease, including plant viral pathogens (Card et al., 2007) as well as fungal pathogens. For example, *Colletotrichum* fungal species use pollen to spread in *Citrus sinensis* (Marques et al., 2013). *Fg* and other *Fusarium* species are present in pollen, as noted earlier, but they are not known to be transmitted to seed (Tejaswini, 2002; Kačániová et al., 2011; Kostić et al., 2019). However, we could only find one report showing pollen-mediated transmission of bacterial pathogens: specifically *Pseudomonas syringae* pv. *actinidiae* (Psa) colonizes kiwifruit anthers both epiphytically and endophytically, resulting in contaminated pollen that transfers Psa to healthy plants (Donati et al., 2018). This raised the fundamental question as to whether maize pollen-associated bacteria are primarily pathogens or not. We hypothesized bacterial taxonomy could provide clues. To address the pathogen question systematically, here, we deliberately cultured pollen bacterial strains from a wide genetic diversity of maize originating from across the Americas, albeit all grown in a common field, and then assigned taxonomy using robust, full-length 16S RNA gene sequencing, as already noted.

The most important finding was that out of 45 unique bacterial species analyzed from maize pollen, only 4 were previously reported as pathogens in maize, specifically *Pantoea agglomerans, Pantoea ananatis*, *Acidovorax avenae*, and *Pseudomonas syringae* (**Figure 3C**; **Supplementary Table S4**):

#### Pantoea ananatis

*P. ananatis* has been reported as the causal agent of white spot disease in maize leaves (Paccola-Meirelles et al., 2001). However, *P. ananatis* is ubiquitous across ecosystems (De Maayer et al., 2014) and can be mutualists, saprophytes, or pathogens (Coutinho and Venter, 2009). In one study, three maize seed *P. ananatis* strains, S6, S7, and S8, differed dramatically in their impacts on host maize, from improving plant growth to being weakly pathogenic, or neutral, respectively (Sheibani-Tezerji et al., 2015). Indeed, the reports concerning *P. ananatis* suggest that it is mostly beneficial to host plant species. It was reported to reduce DON mycotoxin contamination by >50% in wheat spikes infected with *Fg* (Deroo et al., 2022), inhibit the maize-associated fungal pathogen *Lecanicillium aphanocladii in vitro* (Rijavec et al., 2007), suppress rice blast disease in field trials (Someya et al., 2003), and enhance rice growth and yield (Megías et al., 2016; Megías et al., 2017). Intriguingly, *P. ananatis* was shown to rapidly colonize wounds in grapevine before the establishment of the pathogen *Botrytis cinerea*, thereby suppressing mycelia growth and disease (Gasser et al., 2012), similar to our observations concerning strain AS541 at silk wounds in this study (**Figure 9**). Interestingly, *P. ananatis* is a well-known maize seed endophyte (Rijavec et al., 2007; Johnston-Monje and Raizada, 2011), raising the possibility that pollen transmits it. Consistent with this suggestion, *Pantoea* was identified as the most prevalent and abundant taxa in pollinated maize silks (Khalaf et al., 2021).

#### Pantoea agglomerans

*P. agglomerans* is the causal agent of leaf blight, vascular wilt (Morales-Valenzuela et al., 2007), and dry stalk rot diseases in maize (HuiYing et al., 2011). However, *P. agglomerans* has also been reported as a seed endophyte of maize (Johnston-Monje and Raizada, 2011), wheat (Quecine et al., 2012), and rice (Feng et al., 2006). It can promote growth in sugarcane (Quecine et al., 2012) and rice (Feng et al., 2006); enhance the growth of tropical maize in saline soil (Gond et al., 2015); and inhibit a broad range of fungal and bacterial pathogens including the rice blast fungus *Pyricularia grisea* (Kim and Lee, 2019), *Erwinia amylovora* in apple and pear (Pusey et al., 2008), and bacterial wilt in bean (Hsieh et al., 2005).

#### Acidovorax avenae

*A. avenae* causes Bacterial Leaf Blight in maize (Pataky et al., 1997; Munkvold and White, 2016), but is a widespread pathogen across the Poaceae including many cereal crops (Schaad et al., 2008). The evidence for *A. avenae* having beneficial activities in plants is limited to one report of it suppressing Bacterial Fruit Blotch disease in watermelon (Krittidetch et al., 2013).

#### Pseudomonas syringae

*P. syringae* causes Holcus Spot disease in maize (Gonzalez and Vidaver, 1979; Munkvold and White, 2016). However, some strains can be beneficial to host plants. For example, seed inoculation with *P. syringae* enhanced maize growth in compacted saline-sodic soil (Zafar-ul-Hye et al., 2018). Similarly*, P. syringae* pv. *syringae* strain 260-02 was shown to promote plant growth and to exert biocontrol of *P. syringae* pv. tomato strain DC3000, against *Botrytis cinerea* under greenhouse conditions (Passera et al., 2019).

 

The above literature evidence leaves open the question as to whether the above four pollen-associated bacterial species are pathogens or not, especially *P. ananatis* and *P. agglomerans.* Whereas *P. ananatis* and *P. agglomerans* have been reported as non-pathogenic maize seed endophytes, consistent with the pollen-transmitting strains being non-pathogenic, *A. avenae*, and *P. syringae* have not been reported as seed endophytes to the best of our knowledge but have been reported as seed-borne pathogens (Song et al., 2004; Dutta et al., 2014), raising the possibility that pathogenic *A. avenae* and *P. syringae* strains could be transmitted through pollen, which would be a novel finding. A vertically transmitted endophyte would be expected to be more prevalent than a pathogen, and consistent with this, we had 29 pollen isolates of *P. ananatis* (from 13/16 maize accessions), 14 isolates of *P. agglomerans* (from 10/16 maize accessions), but only 3 isolates each of *A. avenae* (from 2/16 maize accessions) and *P. syringae* (from a single maize accession) (**Supplementary Table S2**). Furthermore, it seems unlikely that the majority of these strains were pathogenic and transmitted disease to seed, because the seed and resulting plants, which were grown to maturity (n=30 plants per accession), did not have any of the above visual disease symptoms. As important, the seeds were not locally bulked but directly obtained from seedbanks at the International Maize and Wheat Improvement Center (CIMMYT, Mexico) and the U.S. Department of Agriculture (Germplasm Resources Information Network, GRIN) which have strict quality control standards during seed bulking. An interesting possibility is that there has been a selection for pollen to carry avirulent versions of pathogens to act as competitors, mimicking a common biocontrol strategy (Cotty and Bhatnagar, 1994; He et al., 2021). We also found different OTUs within these 4 species, raising the possibility that some are virulent and others are not. Alternatively, it might be that in maize, these bacterial species can switch between mutualistic and pathogenic states, as has been reported with other endophytes (Eaton et al., 2011; Fesel and Zuccaro, 2016). In summary, the totality of the evidence suggests that it is more likely that the majority of *P. ananatis* and *P. agglomerans* pollen strains are beneficial, or minimally commensal, and more likely that *A. avenae* and *P. syringae* strains are pathogenic. Now that a library of cultured pollen microbes is available, it will be possible to systematically spray them onto diverse maize accessions at different vegetative and reproductive growth stages in different environments to screen for disease symptoms and to test for pathogenicity and/or beneficial traits.

In contrast to the above four bacterial species, none of the other pollen-associated bacterial species in this study, present in at least two maize accessions, has been reported as a pathogen in maize based on the literature, and in fact, there is considerable evidence they may be beneficial (mutualists, symbionts) to host plants, with different species conferring different benefits (**Figure 3C**; **Supplementary Table S4**) – though the literature may be biased against reporting commensals. Based on their species classification, the literature predicts that these remaining non-rare pollen-associated bacteria help plants tolerate abiotic stress; specifically, plant trials show that they can: promote plant growth and biomass; solubilize phosphate, potassium, and zinc; fix nitrogen; assist with nutrient uptake; produce siderophores; enhance chlorophyll content under salinity stress; and enhance plant growth under metal stress conditions (see references in **Supplementary Table S4**). Concerning pests and pathogens, in plant trials, these bacterial species have been shown to: promote insect pest mortality; suppress diverse fungi including *Fusarium* and *Aspergillus* species and associated diseases and mycotoxins (see references in **Supplementary Table S4**).

Therefore, the full-length 16S-based taxonomy of the diverse American cultured maize microbiome adds support to the hypothesis that the vast majority of pollen-associated microbes are not pathogens and may in fact be beneficial or minimally commensal to host plants. As the literature predicted that each pollen-associated bacterial species has a unique suite of beneficial traits, it may be that pollen carries a library of bacteria to combat diverse pathogens and stresses. It could be that a portion of the pollen microbiome occupies the surface of pollen grains (Manirajan et al., 2016; Compant et al., 2021) to suppress bacterial and other pathogens similar to the skin microbiome inhibiting human skin infections (Grice and Segre, 2011; Byrd et al., 2018). In other words, these pollen bacteria may be acting as epiphytes to inhibit competitors. There would be strong natural and farmer selection pressure for such protection.

### Preliminary evidence for long-term natural and human selection for pollen-associated bacteria with beneficial traits

It is intriguing that 16 of the 17 bacterial genera present in the pollen of the major wild ancestor of maize were also isolated from at least one cultivated landrace. At the OTU level, of the 27 bacterial OTUs present in the ancestor, 21 (78%) were shared with the pollen of at least one cultivated landrace (**Figure 4**; **Supplementary Table S2**). One possible interpretation is that these taxa were retained, either through vertical transmission or conserved host compatibility alleles, across the domestication boundary 10,000 years ago (Matsuoka et al., 2002; Warburton et al., 2011; Prasanna, 2012; Bedoya et al., 2017). Pollen flow between teosinte and domesticated maize has been reported to contribute to maize genetic diversification (Van Heerwaarden et al., 2011), and now we hypothesize those findings should be expanded to include bacteria. Furthermore, many OTUs were shared by diverse landraces originally selected by distinct indigenous peoples, across distant latitudes, diverse altitudes, and precipitation zones (**Supplementary Figure S3**). Since the majority of these bacterial taxa are not presumptive pathogens and in fact are reported to promote plant health (**Supplementary Table S4**) as mentioned above, then one explanation, as already noted earlier, is that pollen of the ancestors of modern maize carried beneficial bacteria, then distant farmers independently selected for their retention. As already noted, this interpretation is limited by the fact that most seeds in this study originated from common seedbanks, and plants were subsequently grown in a common Canadian field for only one season. A future experiment is needed that surveys maize pollen in their native habitats.

In terms of beneficial host traits, 8/9 strong anti-*Fusarium* strains came from landraces that originated from the center of diversity of maize in Mexico or the northern migration route, while only one strain came from the southern route (**Figure 2**; **Supplementary Figure S2**; **Figure 5**), perhaps suggestive of a historical influence (e.g. human migration). Furthermore, the accessions that originated from the 5 driest environments in this study gave rise to 6/9 of the strong anti-*Fusarium* strains (AS25, AS283, AS343, AS404, AS541, AS542), while accessions that originated from the 5 wettest environments had zero anti-*Fusarium* strains that we could identify (**Figure 2**; **Supplementary Figure S2**; **Figure 5**). It is intriguing that *F. verticillioides* is more prevalent in dry regions in the Americas (Miller, 2001; Sancho et al., 2017; Pfordt et al., 2020); again, it may be that ancient farmers selected against *F. verticillioides* more than *Fg* and related species. It is noteworthy that only 1/9 strong anti-*Fusarium* strains originated from a highland-derived host, which normally would be associated with high rainfall, but in this case, the host was the landrace Cristalino de Chihuahua, derived from a high desert environment with the least rainfall in this study (**Supplementary Figure S2**; **Figure 5**).

Overall, it may be that long-term selection for the microbial function is equally important as selection for taxonomy. Indeed, this study revealed that the pollen of 8 different maize host accessions originating from 30˚N to 33˚S possessed at least one bacterium with strong anti-*Fusarium* activity – but that this trait could be supplied by taxonomically different species and genera (**Figure 5A**). The strong anti-*Fusarium* activity was found in the extant wild ancestor of modern maize (Parviglumis) as well as cultivated landraces that were reportedly first selected by indigenous peoples at different eras. Indigenous maize farmers may have inadvertently selected for pollen-associated microbes possessing anti-fungal genes (e.g. *phzF*) (**Table 2**) that could suppress *Fusarium* through selection of healthy seeds for replanting. There may have been natural selection against its mycotoxins which disrupt pollen/pollen tube growth as already noted (Kostić et al., 2019).

## Conclusion

In this study, we presented four lines of evidence to suggest that in plants (maize), the male gametophyte (pollen) carries a few bacteria that improve progeny fitness, thereby protecting the genetic contribution of the male gametes. Specifically, we profiled cultured pollen-associated bacteria using full-length 16S sequencing and systematically reviewed the corresponding literature, which predicted that the majority of maize pollen-associated bacteria are not pathogens, but might be beneficial or minimally commensal, based on their taxonomy. In support of the latter prediction, we presented preliminary evidence for the conservation of pollen-associated bacterial species from the major wild ancestor Parviglumis teosinte and its derived landraces cultivated by distinct indigenous peoples across the Americas. However, further experiments are needed to rule out that these bacteria originated from the common environment in which the pollen samples were collected. Regardless of their origin, we showed that the pollen of these diverse wild and cultivated maize accessions possess bacteria with anti-*Fusarium* traits, both *in vitro* and *in planta*; for the *in planta* assay, we sprayed silks to replicate the route that male gametes and *F. graminearum* and other GER-associated *Fusarium* species utilize. Finally, we used live cell confocal microscopy-based imaging to help understand how one pollen-derived strain (AS541) protects the male migration route, specifically by colonizing silk trichomes, silk surfaces, and wounds prior to *Fg* infection, and post-infection, by colonizing and apparently breaking *Fusarium* hyphae. These results infer that the microbiome plays an important role in the competition between eukaryotic male gametes, and acts along the male gamete migration route on host females (Kroh et al., 1979; Walsh and Charlesworth, 1992; Minnaar et al., 2019).

## Data availability statement

The datasets presented in this study can be found in online repositories. The names of the repository/repositories and accession number(s) can be found in the article/**Supplementary Material**.

## Author contributions

AS: Conceptualization, Formal analysis, Investigation, Methodology, Visualization, Writing – original draft. VL-R: Investigation, Writing – review & editing. DB: Investigation, Writing – review & editing. MR: Conceptualization, Funding acquisition, Supervision, Writing – review & editing.

## Supplementary material

Supplementary Figure 1 Summary of the taxonomy of cultured bacteria from pollen of diverse American maize grown in a common field at the phylum, class, and OTU level. **(A)** Diagrammatic sketch of the taxonomies (full-length 16S RNA) of the pollen-associated bacteria based on phylum and class. **(B)** Maximum likelihood (ML) phylogenetic tree of pollen-associated bacteria cultured from different host maize accessions based on unique operational taxonomic units (OTUs). Bootstrap values are indicated above the branches.

Supplementary Figure 2 Predicted genus-level taxonomy of pollen-associated bacteria cultured from different host maize accessions spanning the Americas after growth in a common field. The predicted genera are based on full-length 16S Blastn searches at NCBI. The colors (yellow and dark green) highlight the total number of isolates (including redundant and unique sequences) belonging to the corresponding bacterial genus, arranged by host maize accession. The data is organized from the driest to the wettest environments of source maize plants, differentiated with different color codes (Dry, Moderate, and Wet) with the actual rainfall values (in mm) of the location from which the seeds were collected. The letter ‘H’ denotes a highland origin, ‘M’ denotes a mid-altitude origin, and ‘L’ denotes a lowland origin with respect to source maize plants. The latitudes and countries from where the seeds of host accessions were collected are noted. All plants were grown in Elora, Ontario, Canada.

Supplementary Figure 3 The most prevalent cultured pollen-associated bacteria at the OTU level from diverse American maize after growth in a common field. Full-length 16S RNA sequences sharing the same genus were multi-aligned, and each unique sequence was assigned a distinct OTU number (see Methods for stringency criteria). Shown are the resulting species- and OTU-level taxonomic predictions of pollen-associated bacteria that were cultured from more than one host maize accession. The corresponding species prediction is noted along with the percentage identity to the top match at NCBI in brackets. The data is organized by the rainfall environment of source maize plants with the actual rainfall value (in mm) classified as Dry, Moderate, and Wet. The letter ‘H’ denotes a highland origin, ‘M’ denotes a mid-altitude origin, and ‘L’ denotes a lowland origin with respect to source maize plants. The latitude values and the country of origin of the source maize plants are noted. The most conserved OTUs are highlighted horizontally. All plants were grown in a common field in Elora, Ontario, Canada.

Supplementary Figure 4 Molecular identification and taxonomic characterization of pollen-associated anti-*Fusarium* bacterial strains used in the replicated greenhouse trials (see red arrows). Full-length 16S RNA sequencing, as well as whole genome sequencing, were undertaken to taxonomically identify the pollen-associated anti-*Fusarium* bacterial strains. A BLASTn search using the 16S RNA sequences was performed against the NCBI GenBank database revealing that the closest match to AS25 was *Bacillus toyonensis* (100% identity match); the closest match to AS404 was *Bacillus pseudomycoides* (99.44% identity match), and the best match to AS541 was *Kluyvera intermedia* (98.37% identity match). The Genbank accession numbers for these strains are MW644736, MW644738, and MW370137, respectively (**Supplementary Table S2**). However, the subsequent whole genome sequencing results provided more ambiguous identification as follows: AS25 was predicted to belong to *the Bacillus cereus* group, AS404 was identified as an unclassified *Bacillus*, and AS541 as an unclassified *Pantoea* (**Figure 5A**). Therefore, a 16S rRNA-based neighbor-joining phylogenetic tree of these anti-*Fg* bacterial strains was constructed using the 16S predicted taxonomies, which confirmed that AS25 is closely related to *Bacillus toyonensis*, and AS404 to *Bacillus pseudomyocides.* However, AS541 seemed to be different from the other *Kluyvera intermedia* strains in the phylogenetic tree and hence located on a separate branch in the tree. The sequences which are closely related to these candidate strains were pooled from Genbank, then aligned followed by evolutionary analyses in MEGA X to generate a neighbor-joining tree for all strains, with bootstrap values based on 900 replicates (Saitou and Nei, 1987).

Supplementary Figure 5 Antibiotic susceptibility test using a disc diffusion method to select safe anti-*Fusarium* pollen-associated bacterial strains for the replicated greenhouse trials. **(A)** Anti-*Fusarium* bacterial strains were grown for two days in LB broth at 30°C, at 200 rpm. Then, a 500 µL liquid culture of each bacterial strain was spread evenly on a 150 mm X 15 mm plate containing Muller-Hilton agar. Ten antibiotic discs per plate were placed on the agar surface and pressed gently followed by incubation at 30°C for 24 h. Each antibiotic disc was tested in three different independent plates for each bacterial strain. **(B)** The zones of inhibition were measured in mm and categorized as Susceptible (S): a larger ring of inhibition at >15 mm diameter (green); Moderate (M): a smaller ring of inhibition (less than 15 mm diameter) (yellow); Resistant (R): No ring of inhibition to the antibiotic disc used (red).
