# Supplementary material for: Maize pollen carry bacteria that suppress a fungal pathogen that enters through the male gamete fertilization route
Source: Front Plant Sci. 2024 Jan 10;14:1286199. doi: 10.3389/fpls.2023.1286199 (PMC10806238; doi:10.3389/fpls.2023.1286199)
Supplement: Supplementary Figure 1 — Summary of the taxonomy of cultured bacteria from pollen of diverse American maize grown in a common field at the phylum, class, and OTU level. (A) Diagrammatic sketch of the taxonomies (full-length 16S RNA) of the pollen-associated bacteria based on phylum and class. (B) Maximum likelihood (ML) phylogenetic tree of pollen-associated bacteria cultured from different host maize accessions based on unique operational taxonomic units (OTUs). Bootstrap values are indicated above the branches. [file DataSheet_1.zip › Supplementary Methods.docx]

#### Shrestha et al. - Supplemental Methods

#### Details for *In vitro* Screening for Anti-*Fusarium* Activity, Whole Genome Sequencing and Gene Annotation, Selection of Anti-*Fusarium* Bacterial Strains for Greenhouse Testing, Plant Growth Conditions in Greenhouse Trials, Quantification of *Fusarium* Mycotoxins, and *Fusarium* Vitality Staining Using Light Microscopy

###

### *In vitro* screening for anti-*Fusarium* activity

Dual culture assays were conducted to screen pollen-associated bacteria for anti-*Fusarium* activity *in vitro*. The *Fusarium* strain, *Fg*MT#1 (Genbank Accession OR730875), was isolated courtesy of Michelle Thompson (Raizada Lab, University of Guelph) from Gibberella ear rot diseased maize grain from Southwestern Ontario in the Summer of 2018, received from the Ridgetown Campus, University of Guelph. Each pollen-associated bacterium was cultured in an LB liquid broth (pH 7.2), and grown for 2 days at 30°C with shaking at 200 rpm. The bacterial liquid cultures were then centrifuged for 10 min, followed by resuspension in LB liquid media to an optical density, OD_600_ of 0.4-0.6. The *Fusarium* strain was grown for 72 h (25°C, 120 rpm) in 10 mL of sterile potato dextrose broth media (Catalog # DF0549-17-9, Fisher Scientific, Canada). This 3-day-old *Fusarium strain* was vortexed with sterile, ceramic beads at high speed for 3 min to break the large mycelial mass and create a more uniform *Fusarium*  suspension. This uniform *Fusarium*  mycelium was then added to sterile, melted, cooled (41°C) potato dextrose agar media (1 mL of fungal mycelium into 100 mL of PDA media), swirled around in a bottle to mix in the mycelia, and promptly poured into Petri dishes (100 mm x 15 mm; 50 mL/plate), and allowed to solidify. Seven wells (1 cm diameter/well) were punched into the *Fusarium* -embedded agar using sterile glass tubes, into which the pollen-associated bacterial cultures were added (100 µL/well). These agar plates were then incubated at 25°C for 48 h. The diameter of each zone of inhibition was measured (cm) and pictures were taken. The positive control was: commercial Proline® 480 SC foliar fungicide (registration #28359, Bayer, Calgary, AB, Canada) at a concentration of 1:10 Proline: ddH_2_O. LB buffer was the negative control. Each pollen-associated bacterium was screened independently in triplicate (3 separate Petri dishes, with the positions randomized between plates). The diameter of inhibition zones was modeled with a generalized linear mixed model (GLMM) using PROC GLIMMIX, then analyzed using One-Way ANOVA, and means were compared using Tukey’s pairwise comparison in SAS 9.4 (SAS Institute, Cary, North Carolina, USA) with a significance level of P≤0.05. Here, the comparison of each pollen-associated anti-*Fusarium* bacterium, with the negative control (LB buffer) and the positive control (Proline fungicide) treatments are presented.

### Whole genome sequencing and genome mining

The anti-*Fusarium* bacterial isolates identified from the dual culture assays were grown in LB liquid broth (pH 7.2) for 48 h at 30°C with shaking at 200 rpm. The liquid cultures were used to isolate DNA using the DNeasy®UltraClean Microbial Kit (Catalog # 10196-4, Qiagen, USA) following the manufacturer’s protocol. The isolated genomic DNA was then sent to the Microbial Genome Sequencing Center (MiGS, Pittsburgh, Pennsylvania, USA) for whole genome sequencing using Illumina (NextSeq 2000 platform). At MiGS, quality control, and adapter trimming were undertaken with bcl2fastq (version 2.20.0.445, default parameters) (Illumina, 2022). Species-level taxonomic predictions were undertaken using MetaPhlAn3 (Tool: 3.0.7, December 9, 2020, database version; default parameters + ‘add_viruses) (Beghini et al., 2021). The short read mode of Unicycler (version 0.4.8, default parameters) (Wick et al., 2017) was used as a SPAdes optimizer (Prjibelski et al., 2020) to generate short-read assemblies. Gene annotation employed PGAP (Tool: Build5132, database version January 11, 2021, using default parameters) (Seemann, 2014) and prokka (tool and database version: 1.14.5; using default parameters + ‘—rnammer’ + ‘—rfam’, added ‘—metagenome’ when processing metagenomic/unclassified samples, added ‘—kingdom Viruses’ when processing viral & bacteriophage samples) (Feldgarden et al., 2019). The gene annotation file of each bacterial isolate was exported into an excel sheet and the gene names of interest using their acronyms were searched.

### Suppression of GER in Greenhouse Trials

#### Selection of anti-*Fusarium* bacterial strains for greenhouse testing

Due to ambiguous taxonomic results obtained from the whole genome sequencing for some anti-*Fusarium* bacterial candidates, further antibiotic susceptibility tests were conducted using the disc diffusion method to select safe strains for further greenhouse trials. Discs of 20 different clinical antibiotics were tested against the candidate anti-*Fusarium* strains to determine if they were susceptible or resistant (see Figure S5).

Candidate bacteria were grown in LB broth (pH 7.2) at 30°C, 200 rpm for 48 h, and then 500 µL of each candidate culture (OD_600_ 0.4-0.7) was added to each Petri dish (150 mm X 15 mm) containing 50 mL of Mueller-Hilton agar medium (Catalog # OXCM0337B, Fisher Scientific, Canada). Each bacterial culture was spread evenly on the agar plate using a sterile plate spreader and let to dry for ~5 min. Then, ten different antibiotic discs per plate were placed on the agar surface, evenly spaced, and pressed gently onto the agar with the help of forceps; the plates were then incubated at 30°C for 24 h. Afterward, the plates were checked, and the zones of inhibition were measured with a clear ruler. The plates were subsequently incubated for one additional day to compare zones of inhibition between 24 h and 48 h. Each antibiotic disc was tested in three different independent plates for each candidate bacterium.

#### Greenhouse Trials - Plant Growth Conditions

#### Seed sterilization

Seeds of a moderately susceptible commercial maize hybrid DKC55-05RIB (Bayer Crop Science, Canada) were surface-sterilized using the following procedure: seeds were washed in 0.1% Triton X-100 detergent for 10 min with shaking; the detergent was decanted, and replaced with 3% sodium hypochlorite for 10 min, followed by rinsing with autoclaved, distilled water. This was followed by washing with 95% ethanol for 10 min and then rinsing 5-6 times with autoclaved, distilled water. The last wash was plated on LB and PDA plates at 30°C for 2 days to confirm that the seeds were surface-sterilized effectively. No colonies were observed on those plates.

####

#### Seed germination

The sterilized seeds were germinated on medium-sized vermiculite and kept in a black flat tray with holes at the bottom, below which a white tray was used to hold water. The vermiculite was soaked with water before sowing the seeds. Seeds were sown, covered with a clear plastic lid, and kept in the dark for 5 days. The seedling trays were then placed under light indoors for 36 h, then moved to the greenhouse in the morning and transplanted in the late afternoon to early evening.

#### Seedling transplanting

Pots (20 L size) had holes drilled on the bottom and sides for drainage and were pre-sterilized with disinfectant Virkon® Greenhouse (Registration# 24210, Vetoquinol N-A. Inc., Quebec, Canada), and let to dry before being filled with Turface® clay (Turface Athletics Inc, USA). One-week-old, uniformly sized seedlings were transplanted into each pot (3 seedlings/pot) in the Crop Science Greenhouse Facility, the University of Guelph, on May 25, 2021 (Trial 1) and June 11, 2021 (Trial 2). Shade curtains were initially drawn across the greenhouse zones to prevent light damage to the transplanted seedlings. Drip irrigation (1-min supply at a constant 10 min interval) was started in the greenhouse 3 days before transplanting to ensure that the Turface was sufficiently moist. On the third day after transplanting, the shade curtains were removed to let the seedlings have direct access to natural sunlight.

#### Fertilizer application

Fertilizer (24-10-20 Drip Irrigation, # 10535, Plant-Prod, Brampton, Canada) was added via the same irrigation drip lines at the same time interval (at an injector ratio of 1:200 and 100 PPM nitrogen). Magnesium sulfate was added (1.746 g/L in water) as a starter fertilizer. Additionally, calcium carbonate was added to the pots every week (1 tablespoon/pot), followed by watering the plants to ensure the limestone powder was mixed with water. To prevent very acidic water (pH<3) detected in the irrigation source from entering the drip lines, potassium bicarbonate (20 g/L, pre-soaked in water for 1 h) was added to the fertigation source.

#### Light, airflow, and spacing conditions

Plants were grown to maturity using only natural sunlight, as the trial was conducted in summer. The light levels were measured with a Quantum photosynthetically active radiation (PAR) light meter with a separate sensor (Model #QMSS, Apogee Instruments, USA). At the start of the trial, the light levels were: canopy level (range 1150-1300 µmol m-2 s-1 across the blocks) and pot level (range 1000-1290 µmol m-2 s-1 across the blocks). By the V9 growth stage, the light levels were: canopy level (range 1310-1530 µmol m-2 s-1 across the blocks) and pot level (range 990-1250 µmol m-2 s-1 across the blocks). The evaporative cooler vents were switched on to enable airflow in the greenhouse zones. Two weeks after transplanting, the seedlings were thinned to one seedling remaining per pot. To ensure that the plants were receiving uniform sunlight, all pots within each block were rotated to the next block (6 blocks per trial).

#### Insect pest monitoring and control

To prevent aphids and thrip infestation in the greenhouse zones, sticky yellow bug traps were used. In addition, AMBLYforce^TM^S (*Amblyseius swirskii*) (#A1H, Beneficial Insectary, Redding, CA, USA) was used as biocontrol for aphids. Both of the greenhouse zones had thrips infestation at the seedlings stage which gradually disappeared as the crop matured. Additionally, Trial 1 plants were infested with aphids as well as spider mites at the maturity stage of the crop (once the fertilizer supply was stopped). To control these, the infested plant organs as well as the dead leaves were trimmed away.

#### Pollination

The emergence of silks was regularly monitored once the maize plants began tasseling, which started 46 to 50 days after seed sowing. Since all plants belonged to the same hybrid, the ears were not covered with ear bags. Once sufficient silks had emerged (~48 h after initial silk emergence), deliberate self-pollinations/sibling pollinations were done in the mornings. To collect pollen, tassels were bagged one day before, in the late afternoon. Since this hybrid produced up to 3 ears per plant in the trials, only the upper ear of each plant (the primary ear) was pollinated, treated, and scored.

####

#### Harvesting of ears

Three criteria were taken into account before deciding when to stop the supply of fertilizers and irrigation: hardening of the cobs, size of the kernels, and colour of the cobs (deep yellow/orange). Based on these criteria, fertilizers, and irrigation were stopped 45 days after the last pollination. The plants were then allowed to dry for an additional week before harvesting. Each primary ear was harvested with its husks on, packed in labeled bags with their pot number and stored in cardboard boxes inside the greenhouse zone before disease assessments.

#### Quantification of *Fusarium* Mycotoxins

For *Fusarium* mycotoxin analysis, seeds were pooled from all cobs (4 cobs) from within each treatment in each block and mixed thoroughly; one-third of this mixture was used for mycotoxin analysis. All maize kernels from each sample were ground to a particle size of <850 µm using an M2 Stein mill (Fred Stein Lab, Inc. Atchinson, KS, USA). These ground samples were mixed and then a 10 g subsample was used for mycotoxin analysis. Measurements of grain DON and zearalenone mycotoxins were conducted at Ridgetown Campus, the University of Guelph, as previously described (Limay-Rios and Schaafsma 2021) using liquid chromatography-tandem mass spectrometry (LC-MS/MS). The initial phase was performed using a Nexera XR ultra HPLC with a CTO-20AC column oven, two LC-20ADXR pumps, and a DGU-20A5 degasser attached to a SIL20ACXR autosampler (Shimadzu, Kyoto, Japan) equipped with a 100 µL injection syringe. Mass spectrometry (MS/MS) was performed using a QTRAP 6500 + hybrid triple quadrupole/linear ion trap system equipped with a TurboIonSpray probe in the electrospray ionization (ESI) source (AB SCIEX, Concord, Canada). Toxin data were statistically modeled with a GLMM using PROC GLIMMIX, then analyzed using One-Way ANOVA Dunnett’s comparisons in SAS version 9.4 (SAS Institute, Cary, NC) with a significance level of (P≤0.05).

### *Fusarium* Vitality Staining Using Light Microscopy

To determine whether pollen-associated bacteria had direct fungicidal activity against a GER-associated *Fusarium strain*, light microscopy was undertaken. Each bacterium was cultured in LB liquid (pH 7.2) by incubating at 30°C at 200 rpm for 48 h. *Fusarium* was cultured in Potato Dextrose Broth by incubating at 25°C at 120 rpm for 72 h. Microscopic slides were prepared with a thin layer of one mL PDA which was allowed to solidify. 50 µL of *Fusarium* mycelia was placed in the center of each microscopic slide, along with 50 µL of candidate pollen bacterial culture to one side of *Fusarium*, and LB buffer to the opposite side as the negative control. Positive control slides consisted of Proline fungicide (diluted 1:10 with ddH_2_O) in place of bacterial cultures. Microscopic slides were placed inside Petri dishes with their lids closed and incubated at 25°C. After 24 h, the slides were stained with the vitality stain Evans blue (Catalog # E2129, Sigma Aldrich®, Missouri, USA) by placing 1mL of stain on each slide, followed by a 5 min incubation at room temperature, then washing 3-4 times with autoclaved distilled water. Pictures were captured using a CCD camera attached to a light microscope. There were 3 replicates for each bacterial candidate, with each slide placed in a separate Petri dish.

**References**

Beghini, F., Mciver, L. J., Blanco-Míguez, A., 1​, ​, Dubois, L., Asnicar, F., et al. (2021). Integrating taxonomic, functional, and strain-level profiling of diverse microbial communities with bioBakery 3. *Elife*. 10, e65088. doi: 10.1101/2020.11.19.388223.

Feldgarden, M., Brover, V., Haft, D. H., Prasad, A. B., Slotta, D. J., Tolstoy, I., et al. (2019). Validating the AMRFinder tool and resistance gene database by using antimicrobial resistance genotype-phenotype correlations in a collection of isolates. *Antimicrob. Agents Chemother.* 63, e00483-19. doi: 10.1128/AAC.00483-19.

Illumina (2022). bcl2fastq: A proprietary illumina software for the conversion of bcl files to basecalls. Available at: https://support.illumina.com/sequencing/sequencing_software/bcl2fastq-conversion-software.html.

Limay-Rios, V., and Schaafsma, A. W. (2021). Relationship between mycotoxin content in winter wheat grain and aspirated dust collected during harvest and after storage. *ACS Omega* 6, 1857–1871. doi: 10.1021/acsomega.0c04256.

Prjibelski, A., Antipov, D., Meleshko, D., Lapidus, A., and Korobeynikov, A. (2020). Using SPAdes De Novo Assembler. *Curr. Protoc. Bioinforma.* 70, e102. doi: 10.1002/CPBI.102.

Seemann, T. (2014). Genome analysis Prokka: rapid prokaryotic genome annotation. *Bioinformatic Appl. Note* 30, 2068–2069. doi: 10.1093/bioinformatics/btu153.

Wick, R. R., Judd, L. M., Gorrie, C. L., and Holt, K. E. (2017). Unicycler: Resolving bacterial genome assemblies from short and long sequencing reads. *PLOS Comput. Biol.* 13, e1005595. doi: 10.1371/JOURNAL.PCBI.1005595.
