# Supplementary material for: Maize pollen carry bacteria that suppress a fungal pathogen that enters through the male gamete fertilization route
Source: Front Plant Sci. 2024 Jan 10;14:1286199. doi: 10.3389/fpls.2023.1286199 (PMC10806238; doi:10.3389/fpls.2023.1286199)
Supplement: Supplementary Figure 1 — Summary of the taxonomy of cultured bacteria from pollen of diverse American maize grown in a common field at the phylum, class, and OTU level. (A) Diagrammatic sketch of the taxonomies (full-length 16S RNA) of the pollen-associated bacteria based on phylum and class. (B) Maximum likelihood (ML) phylogenetic tree of pollen-associated bacteria cultured from different host maize accessions based on unique operational taxonomic units (OTUs). Bootstrap values are indicated above the branches. [file DataSheet_1.zip › Supplementary Tables S2 to S6.docx]

**Table S2.** A complete master list of pollen-associated bacterial strains isolated from diverse American host maize accessions with their closest taxonomic identities based on full-length 16S RNA sequence BLAST searches in Genbank. The 16S RNA sequences were deposited in Genbank and are retrievable by the accession numbers. Also shown are the dual culture assay results, describing the effect of each bacterial strain on the growth or colour of GER-associated *Fusarium* isolate FgMT#1 *in vitro*.

| **Host** | **Sample Name** | **Strain ID** | **OTUs No.** | **Predicted Genus** | **Predicted Species** | **% Query cover** | **% identity** | **Accession number** | **Dual culture assay** |
| --- | --- | --- | --- | --- | --- | --- | --- | --- | --- |
| Camelia | R1-19P-F | AS-296 | OTU1 | *Curtobacterium* | *pusillum* | 99 | 99.49% | OL604278 | No effect |
| Camelia | R1-19P-K | AS301 | OTU1 | *Curtobacterium* | *flaccumfaciens* | 99 | 99.42% | MW370160 |  |
| Camelia | R1-19P-A | AS-283 | OTU2 | *Erwinia* | *gerundensis* | 98 | 98.81% | OL604275 | Strong inhibition |
| Camelia | R1-19P-J | AS289 | OTU3 | *Exiguobacterium* | *indicum* | 100 | 99.10% | MW369975 | No effect |
| Camelia | R1-19P-C | AS294 | OTU4 | *Frigoribacterium* | *faeni* | 99 | 99.21% | MW369979 | No effect |
| Camelia | R1-19P-D | AS295 | OTU5 | *Microbacterium* | *zeae* | 99 | 99.28% | MW369980 | No effect |
| Camelia | R1-19P-B | AS284 | OTU6 | *Pantoea* | *ananatis* | 99 | 98.94% | MW369972 |  |
| Camelia | R1-19P-G | AS286 | OTU7 | *Pantoea* | *agglomerans* | 100 | 97.46% | MW369973 |  |
| Camelia | R1-19P-H | AS287 | OTU6 | *Pantoea* | *ananatis* | 99 | 99.36% | MW369974 |  |
| Camelia | R1-19P-M | AS302 | OTU6 | *Pantoea* | *ananatis* | 99 | 99.36% | MW370161 | Color change + overgrowth |
| Canilla | R1-21P-G | AS382 | OTU8 | *Erwinia* | *aphidicola* | 99 | 98.87% | MW370224 |  |
| Canilla | R1-21P-K | AS-564 | OTU9 | *Methylobacterium* | *cerastii* | 99 | 98.59% | OL604330 |  |
| Canilla | R1-21P-F | AS-381 | OTU10 | *Rahnella* | *aquatilis* | 100 | 99.36% | OL604294 | Strong inhibition |
| Canilla | R1-21P-A | AS-377 | OTU11 | *Rosenbergiella* | *epipactidis* | 100 | 100.00% | MW644734 | Strong inhibition |
| Canilla | R1-21P-B | AS-378 | OTU12 | *Rosenbergiella* | *collisarenosi* | 100 | 99.83% | OL604397 |  |
| Canilla | R1-21P-C | AS379 | OTU11 | *Rosenbergiella* | *epipactidis* | 99 | 99.65% | MW370221 |  |
| Canilla | R1-21P-E | AS-380 | OTU11 | *Rosenbergiella* | *epipactidis* | 100 | 99.93% | OL604293 |  |
| Canilla | R1-21P-H | AS383 | OTU11 | *Rosenbergiella* | *epipactidis* | 100 | 99.36% | MW370225 | No effect |
| Canilla | R1-21P-I | AS-384 | OTU11 | *Rosenbergiella* | *epipactidis* | 99 | 100.00% | OL604295 |  |
| Canilla | R1-21P-L | AS410 | OTU11 | *Rosenbergiella* | *epipactidis* | 99 | 99.72% | MW370239 |  |
| Cateto Nortista | R1-22P-F | AS463 | OTU13 | *Acinetobacter* | *baylyi* | 99 | 99.28% | MW370076 | color change. |
| Cateto Nortista | R1-22P-K | AS-468 | OTU14 | *Chryseobacterium* | *hagamense* | 99 | 98.91% | OL604313 | No effect |
| Cateto Nortista | R1-22P-N | AS470 | OTU14 | *Chryseobacterium* | *hagamense* | 99 | 98.20% | MW370083 |  |
| Cateto Nortista | R1-22P-O | AS476 | OTU18 | *Enterobacter* | *ludwigii* | 99 | 99.72% | MW370088 |  |
| Cateto Nortista | R1-22P-G | AS-464 | OTU15 | *Microbacterium* | *testaceum* | 100 | 99.70% | OL604311 | No effect |
| Cateto Nortista | R1-22P-I | AS-466 | OTU16 | *Pantoea* | *agglomerans* | 100 | 99.17% | OL604414 |  |
| Cateto Nortista | R1-22P-P | AS474 | OTU17 | *Pantoea* | *agglomerans* | 99 | 95.29% | MW370086 | color change |
| Cateto Nortista | R1-22P-C | AS475 | OTU19 | *Pantoea* | *ananatis* | 100 | 96.12% | MW370087 | No effect |
| Cateto Nortista | R1-22P-A | AS461 | OTU20 | *Pseudomonas* | *lurida* | 99 | 99.79% | MW370074 | No effect |
| Cateto Nortista | R1-22P-H | AS-465 | OTU20 | *Pseudomonas* | *lurida* | 99 | 99.86% | OL604312 |  |
| Cateto Nortista | R1-22P-B | AS-462 | OTU21 | *Stenotrophomonas* | *maltophilia* | 99 | 100.00% | OL604413 | No effect |
| Cateto Nortista | R1-22P-J | AS-467 | OTU21 | *Stenotrophomonas* | *tumulicola* | 99 | 99.76% | OL604415 | No effect |
| Cateto Nortista | R1-22P-L | AS-469 | OTU21 | *Stenotrophomonas* | *tumulicola* | 100 | 99.52% | OL604416 |  |
| Chapalote | R1-4P-E | AS347 | OTU22 | *Aureimonas* | *ureilytica* | 99 | 99.19% | MW370196 | No effect |
| Chapalote | R1-4P-G | AS348 | OTU23 | *Deinococcus* | *arenae* | 99 | 99.71% | MW370197 | No effect |
| Chapalote | R1-4P-A | AS-343 | OTU2 | *Erwinia* | *gerundensis* | 98 | 98.59% | MW644735 | Minor inhibition |
| Chapalote | R1-4P-C | AS345 | OTU15 | *Microbacterium* | *testaceum* | 99 | 99.64% | MW370195 | biofilm |
| Chapalote | R1-4P-N | AS367 | OTU5 | *Microbacterium* | *zeae* | 99 | 99.28% | MW370212 | No effect |
| Chapalote | R1-4P-D | AS-346 | OTU16 | *Pantoea* | *anthophila* | 100 | 99.85% | OL604393 | color change |
| Chapalote | R1-4P-I | AS-350 | OTU16 | *Pantoea* | *agglomerans* | 100 | 99.58% | OL604394 |  |
| Chapalote | R1-4P-O | AS368 | OTU7 | *Pantoea* | *agglomerans* | 99 | 97.25% | MW370213 |  |
| Chapalote | R1-4P-B | AS344 | OTU24 | *Pseudomonas* | *fulva* | 100 | 99.64% | MW383967 | color change |
| Chapalote | R1-4P-K | AS352 | OTU24 | *Pseudomonas* | *fulva* | 99 | 99.15% | MW370199 | color change |
| Chapalote | R1-4P-L | AS353 | OTU25 | *Pseudomonas* | *psychrotolerans* | 99 | 99.01% | MW370200 | Color change |
| Chapalote | R1-4P-H | AS349 | OTU11 | *Rosenbergiella* | *epipactidis* | 99 | 96.37% | MW367621 |  |
| Chapalote | R1-4P-R | AS371 | OTU11 | *Rosenbergiella* | *epipactidis* | 100 | 99.65% | MW370215 |  |
| Chapalote | R1-4P-M | AS354 | OTU26 | *Siphonobacter* | *intestinalis* | 100 | 96.08% | MW370201 | color change |
| Chapalote | R1-4P-Q | AS370 | OTU26 | *Siphonobacter* | *intestinalis* | 99 | 96.10% | MW370214 |  |
| Confite Morocho | R1-25P-R | AS486 | OTU27 | *Bacillus* | *wiedmannii* | 99 | 99.65% | MW370093 | light clearing |
| Confite Morocho | R1-25P-S | AS487 | OTU27 | *Bacillus* | *wiedmannii* | 100 | 99.65% | MW370094 |  |
| Confite Morocho | R1-25P-E | AS-488 | OTU27 | *Bacillus* | *proteolyticus* | 100 | 99.71% | OL604316 |  |
| Confite Morocho | R1-25P-D | AS454 | OTU28 | *Brachybacterium* | *squillarum* | 99 | 96.34% | MW370067 | No effect |
| Confite Morocho | R1-25P-P | AS478 | OTU29 | *Brachybacterium* | *squillarum* | 99 | 97.26% | MW370090 |  |
| Confite Morocho | R1-25P-M | AS-459 | OTU30 | *Deinococcus* | *radiotolerans* | 100 | 99.58% | OL604412 | No effect |
| Confite Morocho | R1-25P-G | AS-456 | OTU2 | *Erwinia* | *gerundensis* | 98 | 98.89% | OL604309 | Strong inhibition |
| Confite Morocho | R1-25P-L | AS505 | OTU31 | *Kocuria* | *koreensis* | 99 | 99.29% | MW370109 | No effect |
| Confite Morocho | R1-25P-T | AS506 | OTU31 | *Kocuria* | *koreensis* | 100 | 99.14% | MW370110 |  |
| Confite Morocho | R1-25P-H | AS-518 | OTU31 | *Kocuria* | *koreensis* | 99 | 99.56% | OL604318 |  |
| Confite Morocho | R1-25P-J | AS457 | OTU24 | *Pseudomonas* | *cremoricolorata* | 98 | 99.57% | MW370070 | color change |
| Confite Morocho | R1-25P-N | AS-460 | OTU25 | *Pseudomonas* | *oryzihabitans* | 100 | 99.57% | OL604310 | color change |
| Confite Morocho | R1-25P-K | AS458 | OTU11 | *Rosenbergiella* | *epipactidis* | 99 | 99.65% | MW370071 | color change |
| Confite Morocho | R1-25P-A | AS-451 | OTU32 | *Staphylococcus* | *xylosus* | 100 | 99.52% | OL604410 |  |
| Confite Morocho | R1-25P-B | AS-452 | OTU32 | *Staphylococcus* | *kloosii* | 99 | 99.71% | OL604411 | No effect |
| Confite Morocho | R1-25P-C | AS453 | OTU33 | *Staphylococcus* | *kloosii* | 100 | 100.00% | MW385282 |  |
| Confite Morocho | R1-25P-F | AS-455 | OTU34 | *Staphylococcus* | *lentus* | 100 | 100.00% | OL604308 |  |
| Cristalino de Chihuahua | R1-7P-K | AS-29 | OTU35 | *Acidovorax* | *avenae* | 100 | 99.34% | OL604276 | light clearing |
| Cristalino de Chihuahua | R1-7P-X | AS-42 | OTU35 | *Acidovorax* | *avenae* | 100 | 99.11% | OL604303 |  |
| Cristalino de Chihuahua | L1-7P-O | AS-14 | OTU36 | *Agrobacterium* | *larrymoorei* | 100 | 99.85% | OL604244 | No effect |
| Cristalino de Chihuahua | R1-7P-O | AS-33 | OTU36 | *Agrobacterium* | *larrymoorei* | 99 | 99.78% | OL604280 | No effect |
| Cristalino de Chihuahua | L1-7P-E | AS5 | OTU36 | *Agrobacterium* | *larrymoorei* | 99 | 99.85% | MW369758 | No effect |
| Cristalino de Chihuahua | L1-7P-U | AS-50 | OTU36 | *Agrobacterium* | *larrymoorei* | 100 | 99.85% | OL604317 |  |
| Cristalino de Chihuahua | R1-7P-D | AS64 | OTU37 | *Atlantibacter* | *hermannii* | 100 | 94.21% | MW383958 | No effect |
| Cristalino de Chihuahua | R1-7P-A3 | AS49 | OTU38 | *Azospirillum* | *melinis* | 99 | 97.73% | MW369788 |  |
| Cristalino de Chihuahua | L1-7P-V | AS20 | OTU39 | *Bacillus* | *pumilus* | 100 | 99.44% | MW369768 | No effect |
| Cristalino de Chihuahua | R1-7P-C | AS24 | OTU27 | *Bacillus* | *pseudomycoides* | 97 | 95.57% | MW367606 | color change |
| Cristalino de Chihuahua | R1-7P-E | AS-25 | OTU27 | *Bacillus* | *toyonensis/thuringiensis* | 100 | 100.00% | MW644736 | Strong inhibition |
| Cristalino de Chihuahua | L1-7P-D | AS4 | OTU27 | *Bacillus* | *mycoides* | 100 | 99.86% | MW369757 | color change |
| Cristalino de Chihuahua | L1-7P-M | AS12 | OTU18 | *Enterobacter* | *ludwigii* | 99 | 99.14% | MW369763 | No effect |
| Cristalino de Chihuahua | L1-7P-W | AS21 | OTU18 | *Enterobacter* | *ludwigii* | 99 | 99.72% | MW369769 |  |
| Cristalino de Chihuahua | R1-7P-G | AS-27 | OTU18 | *Enterobacter* | *ludwigii* | 99 | 99.57% | OL604270 |  |
| Cristalino de Chihuahua | R1-7P-Y | AS-43 | OTU18 | *Enterobacter* | *ludwigii* | 100 | 99.71% | OL604306 |  |
| Cristalino de Chihuahua | L1-7P-H | AS8 | OTU18 | *Enterobacter* | *ludwigii* | 100 | 99.64% | MW369761 | light clearing |
| Cristalino de Chihuahua | R1-7P-A | AS-22 | OTU3 | *Exiguobacterium* | *acetylicum* | 100 | 99.64% | OL604250 | No effect |
| Cristalino de Chihuahua | L1-7P-S | AS18 | OTU31 | *Kocuria* | *koreensis* | 100 | 99.79% | MW369767 | No effect |
| Cristalino de Chihuahua | R1-7P-H | AS45 | OTU31 | *Kocuria* | *koreensis* | 99 | 99.57% | MW369785 | No effect |
| Cristalino de Chihuahua | L1-7P-F | AS-6 | OTU40 | *Lysinibacillus* | *pakistanensis* | 99 | 99.14% | OL604331 | light clearing |
| Cristalino de Chihuahua | L1-7P-G | AS-7 | OTU40 | *Lysinibacillus* | *pakistanensis* | 100 | 99.14% | OL604332 |  |
| Cristalino de Chihuahua | L1-7P-J | AS-10 | OTU41 | *Microbacterium* | *testaceum* | 99 | 99.20% | OL604243 | biofilm |
| Cristalino de Chihuahua | L1-7P-Q | AS16 | OTU15 | *Microbacterium* | *testaceum* | 100 | 99.50% | MW369765 |  |
| Cristalino de Chihuahua | R1-7P-M | AS31 | OTU15 | *Microbacterium* | *testaceum* | 99 | 99.50% | MW369776 | color change |
| Cristalino de Chihuahua | R1-7P-Q | AS-35 | OTU5 | *Microbacterium* | *zeae* | 100 | 99.93% | OL604285 | No effect |
| Cristalino de Chihuahua | R1-7P-A2 | AS48 | OTU15 | *Microbacterium* | *testaceum* | 98 | 99.71% | MW369787 |  |
| Cristalino de Chihuahua | L1-7P-A | AS-1 | OTU42 | *Pantoea* | *ananatis* | 100 | 99.67% | OL604364 |  |
| Cristalino de Chihuahua | L1-7P-N | AS-13 | OTU16 | *Pantoea* | *agglomerans* | 100 | 99.17% | OL604365 | No effect |
| Cristalino de Chihuahua | R1-7P-P | AS-34 | OTU16 | *Pantoea* | *agglomerans* | 100 | 98.92% | OL604392 | No effect |
| Cristalino de Chihuahua | R1-7P-U | AS-39 | OTU6 | *Pantoea* | *allii* | 99 | 99.78% | OL604298 | color change |
| Cristalino de Chihuahua | R1-7P-I | AS-28 | OTU43 | *Pedobacter* | *suwonensis* | 100 | 99.41% | MW644747 | biofilm |
| Cristalino de Chihuahua | L1-7P-R | AS17 | OTU44 | *Pseudomonas* | *oryzihabitans* | 99 | 99.79% | MW369766 | color change |
| Cristalino de Chihuahua | R1-7P-L | AS-30 | OTU45 | *Pseudomonas* | *psychrotolerans* | 100 | 99.42% | OL604279 |  |
| Cristalino de Chihuahua | R1-7P-A1 | AS47 | OTU45 | *Pseudomonas* | *psychrotolerans* | 99 | 99.14% | MW369786 |  |
| Cristalino de Chihuahua | R1-7P-R | AS-36 | OTU46 | *Sphingomonas* | *sanguinis* | 99 | 98.28% | OL604287 | color change |
| Cristalino de Chihuahua | R1-7P-V | AS-40 | OTU47 | *Sphingomonas* | *panni* | 100 | 99.19% | OL604300 | No effect |
| Cristalino de Chihuahua | R1-7P-J | AS44 | OTU47 | *Sphingomonas* | *panni* | 99 | 99.04% | MW369784 |  |
| Cristalino de Chihuahua | L1-7P-B | AS2 | OTU34 | *Staphylococcus* | *sciuri* | 99 | 99.79% | MW349704 | No effect |
| Cristalino de Chihuahua | L1-7P-C | AS3 | OTU33 | *Staphylococcus* | *xylosus* | 100 | 99.79% | MW349705 | No effect |
| Cristalino de Chihuahua | L1-7P-K | AS-269 | OTU48 | *Streptomyces* | *coelescens* | 100 | 99.92% | OL604269 | No effect |
| Dente Branco | R1-18P-F | AS-262 | OTU49 | *Chryseobacterium* | *hagamense* | 100 | 99.01% | OL604383 |  |
| Dente Branco | R1-18P-N | AS-263 | OTU14 | *Chryseobacterium* | *hagamense* | 99 | 98.77% | OL604267 | No effect |
| Dente Branco | R1-18P-A | AS-199 | OTU8 | *Erwinia* | *aphidicola* | 99 | 99.65% | OL604246 |  |
| Dente Branco | R1-18P-B | AS200 | OTU8 | *Erwinia* | *aphidicola* | 99 | 99.50% | MW369905 | light clearing |
| Dente Branco | R1-18P-C | AS201 | OTU8 | *Erwinia* | *aphidicola* | 99 | 99.43% | MW369906 |  |
| Dente Branco | R1-18P-D | AS202 | OTU8 | *Erwinia* | *aphidicola* | 99 | 99.43% | MW369907 |  |
| Dente Branco | R1-18P-G | AS204 | OTU8 | *Erwinia* | *aphidicola* | 99 | 99.29% | MW369909 |  |
| Dente Branco | R1-18P-K | AS-207 | OTU50 | *Erwinia* | *persicina* | 100 | 99.76% | OL604373 |  |
| Dente Branco | R1-18P-L | AS-208 | OTU8 | *Erwinia* | *aphidicola* | 100 | 99.71% | OL604247 |  |
| Dente Branco | R1-18P-M | AS209 | OTU8 | *Erwinia* | *aphidicola* | 99 | 99.64% | MW369913 | light clearing |
| Dente Branco | R1-18P-P | AS210 | OTU8 | *Erwinia* | *aphidicola* | 99 | 99.36% | MW369914 |  |
| Dente Branco | R1-18P-Q | AS-236 | OTU8 | *Erwinia* | *aphidicola* | 100 | 99.64% | OL604259 |  |
| Dente Branco | R1-18P-R | AS237 | OTU8 | *Erwinia* | *aphidicola* | 99 | 99.22% | MW369934 |  |
| Dente Branco | R1-18P-O | AS-240 | OTU8 | *Erwinia* | *aphidicola* | 99 | 99.29% | OL604260 |  |
| Dente Branco | R1-18P-J | AS241 | OTU8 | *Erwinia* | *aphidicola* | 99 | 99.22% | MW369938 |  |
| Dente Branco | R1-18P-E | AS-203 | OTU16 | *Pantoea* | *agglomerans* | 100 | 99.52% | OL604370 | No effect |
| Dente Branco | R1-18P-H | AS-205 | OTU16 | *Pantoea* | *agglomerans* | 99 | 99.01% | OL604371 |  |
| Dente Branco | R1-18P-I | AS-206 | OTU16 | *Pantoea* | *agglomerans* | 100 | 99.30% | OL604372 |  |
| Dente Branco | R1-18P-U | AS-238 | OTU16 | *Pantoea* | *agglomerans* | 100 | 99.38% | OL604378 |  |
| Dente Branco | R1-18P-V | AS-239 | OTU16 | *Pantoea* | *agglomerans* | 100 | 99.15% | OL604379 | light clearing |
| Jala | R1-9P-F | AS-362 | OTU51 | *Acinetobacter* | *soli* | 98 | 99.78% | OL604289 |  |
| Jala | R1-9P-Q | AS373 | OTU84 | *Bacillus* | *metaterium* | 99 | 99.51% | MW370216 | No effect |
| Jala | R1-9P-M | AS-374 | OTU52 | *Bacillus* | *pseudomycoides* | 100 | 99.93% | OL604291 | No effect |
| Jala | R1-9P-G | AS-359 | OTU42 | *Pantoea* | *agglomerans* | 100 | 99.39% | OL604396 | No effect |
| Jala | R1-9P-B | AS-356 | OTU54 | *Pseudomonas* | *extremorientalis* | 99 | 99.78% | OL604286 | color change |
| Jala | R1-9P-C | AS357 | OTU24 | *Pseudomonas* | *syringae* | 99 | 99.64% | MW370204 | No effect |
| Jala | R1-9P-E | AS358 | OTU24 | *Pseudomonas* | *syringae* | 99 | 99.71% | MW370205 |  |
| Jala | R1-9P-N | AS-375 | OTU54 | *Pseudomonas* | *extremorientalis* | 99 | 99.78% | OL604292 | color change |
| Jala | R1-9P-P | AS376 | OTU24 | *Pseudomonas* | *syringae* | 99 | 99.64% | MW370219 | light clearing |
| Jala | R1-9P-J | AS372 | OTU11 | *Rosenbergiella* | *collisarenosi* | 100 | 99.63% | MW383968 | color change |
| Jala | R1-9P-A | AS-355 | OTU55 | *Serratia* | *liquefaciens* | 100 | 99.77% | OL604395 | No effect |
| Jala | R1-9P-K | AS-360 | OTU56 | *Serratia* | *marcescens* | 99 | 99.86% | OL604288 | light clearing |
| Jala | R1-9P-L | AS-361 | OTU57 | *Serratia* | *ficaria* | 100 | 98.99% | MW644737 | Minor inhibition |
| Kulli | R1-24P-B | AS416 | OTU13 | *Acinetobacter* | *baylyi* | 99 | 99.71% | MW370243 | color change |
| Kulli | R1-24P-K | AS420 | OTU13 | *Acinetobacter* | *baylyi* | 99 | 99.07% | MW370245 |  |
| Kulli | R1-24P-N | AS422 | OTU13 | *Acinetobacter* | *baylyi* | 99 | 99.36% | MW370247 |  |
| Kulli | R1-24P-P | AS-483 | OTU58 | *Arthrobacter* | *pascens* | 100 | 99.48% | OL604315 | light clearing |
| Kulli | R1-24P-A | AS415 | OTU53 | *Bacillus* | *simplex* | 99 | 99.37% | MW370242 | color change |
| Kulli | R1-24P-J | AS419 | OTU53 | *Bacillus* | *simplex* | 100 | 99.56% | MW385278 |  |
| Kulli | R1-24P-L | AS421 | OTU52 | *Bacillus* | *pseudomycoides* | 99 | 99.58% | MW370246 | No effect |
| Kulli | R1-24P-I | AS-418 | OTU3 | *Exiguobacterium* | *acetylicum* | 100 | 99.62% | OL604403 | No effect |
| Kulli | R1-24P-F | AS-535 | OTU4 | *Frigoribacterium* | *faeni* | 99 | 99.42% | OL604319 | No effect |
| Kulli | R1-24P-C | AS-562 | OTU59 | *Massilia* | *brevitalea* | 99 | 98.50% | OL604329 | No effect |
| Kulli | R1-24P-S | AS-569 | OTU60 | *Methylobacterium* | *indicum* | 99 | 94.84% | OL604421 | No effect |
| Kulli | R1-24P-R | AS472 | OTU15 | *Microbacterium* | *testaceum* | 99 | 99.64% | MW370084 | No effect |
| Kulli | R1-24P-D | AS-417 | OTU16 | *Pantoea* | *agglomerans* | 100 | 98.52% | OL604402 | No effect |
| Kulli | R1-24P-G | AS450 | OTU25 | *Pseudomonas* | *psychrotolerans* | 100 | 99.08% | MW370261 | color change |
| Kulli | R1-24P-E | AS449 | OTU65 | *Sphingomonas* | *roseiflava* | 100 | 99.85% | MW383970 | No effect |
| Kulli | R1-24P-Q | AS-473 | OTU46 | *Sphingomonas* | *paucimobilis* | 99 | 99.26% | OL604314 | No effect |
| Nal-Tel | R1-10P-G | AS-387 | OTU51 | *Acinetobacter* | *soli* | 100 | 100.00% | OL604296 | color change |
| Nal-Tel | R1-10P-H | AS-388 | OTU13 | *Acinetobacter* | *baylyi* | 99 | 99.71% | OL604297 | color change |
| Nal-Tel | R1-10P-J | AS389 | OTU13 | *Acinetobacter* | *baylyi* | 99 | 99.50% | MW370230 |  |
| Nal-Tel | R1-10P-B | AS-404 | OTU52 | *Bacillus* | *pseudomycoides* | 99 | 99.44% | MW644738 | strong inhibition |
| Nal-Tel | R1-10P-F | AS-406 | OTU85 | *Bacillus* | *aryabhattai* | 100 | 99.93% | OL604301 | No effect |
| Nal-Tel | R1-10P-C | AS405 | OTU5 | *Microbacterium* | *zeae* | 100 | 99.36% | MW370235 | No effect |
| Nal-Tel | R1-10P-I | AS-407 | OTU42 | *Pantoea* | *ananatis* | 100 | 99.75% | OL604302 | light clearing |
| Nal-Tel | R1-10P-L | AS-408 | OTU42 | *Pantoea* | *anthophila* | 100 | 99.87% | OL604399 |  |
| Nal-Tel | R1-10P-D | AS385 | OTU24 | *Pseudomonas* | *parafulva* | 99 | 99.07% | MW370227 | No effect |
| Nal-Tel | R1-10P-A | AS403 | OTU25 | *Pseudomonas* | *psychrotolerans* | 99 | 99.29% | MW370233 | color change |
| Nal-Tel | R1-10P-M | AS-539 | OTU75 | *Rathayibacter* | *festucae* | 99 | 99.86% | OL604322 |  |
| Oloton | R1-16P-A | AS-270 | OTU13 | *Acinetobacter* | *baylyi* | 99 | 99.14% | OL604271 | color change |
| Oloton | R1-16P-H | AS-274 | OTU51 | *Acinetobacter* | *soli* | 100 | 99.38% | OL604387 |  |
| Oloton | R1-16P-K | AS276 | OTU51 | *Acinetobacter* | *soli* | 99 | 99.01% | MW369964 | color change + clearing |
| Oloton | R1-16P-L | AS-277 | OTU13 | *Acinetobacter* | *baylyi* | 99 | 99.21% | OL604272 |  |
| Oloton | R1-16P-O | AS-280 | OTU13 | *Acinetobacter* | *baylyi* | 99 | 99.14% | OL604274 |  |
| Oloton | R1-16P-M | AS278 | OTU27 | *Bacillus* | *mycoides* | 100 | 99.51% | MW369966 |  |
| Oloton | R1-16P-R | AS300 | OTU64 | *Brevundimonas* | *vesicularis* | 100 | 99.92% | MW383965 | No effect |
| Oloton | R1-16P-E | AS-271 | OTU8 | *Erwinia* | *aphidicola* | 99 | 99.84% | OL604385 |  |
| Oloton | R1-16P-G | AS-273 | OTU50 | *Erwinia* | *rhapontici* | 100 | 100.00% | OL604386 |  |
| Oloton | R1-16P-P | AS-281 | OTU50 | *Erwinia* | *rhapontici* | 99 | 99.84% | OL604388 | light clearing |
| Oloton | R1-16P-Q | AS-282 | OTU8 | *Erwinia* | *rhapontici* | 99 | 99.72% | OL604389 |  |
| Oloton | R1-16P-I | AS275 | OTU67 | *Kluyvera* | *intermedia* | 99 | 98.16% | MW369963 | color change + clearing |
| Oloton | R1-16P-C | AS291 | OTU15 | *Microbacterium* | *testaceum* | 100 | 99.42% | MW369976 | No effect |
| Oloton | R1-16P-F | AS272 | OTU6 | *Pantoea* | *ananatis* | 100 | 99.15% | MW369960 | No effect |
| Oloton | R1-16P-B | AS290 | OTU16 | *Pantoea* | *ananatis* | 100 | 98.08% | MW385273 |  |
| Oloton | R1-16P-J | AS-293 | OTU68 | *Pedobacter* | *suwonensis* | 99 | 98.99% | OL604277 | No effect |
| Oloton | R1-16P-N | AS-279 | OTU24 | *Pseudomonas* | *parafulva* | 99 | 99.50% | OL604273 | color change |
| Oloton | R1-16P-D | AS-292 | OTU46 | *Sphingomonas* | *paucimobilis* | 99 | 99.26% | OL604390 | No effect |
| Palomero Toluqueno | R1-13P-M | AS-393 | OTU85 | *Bacillus* | *aryabhattai* | 100 | 100.00% | OL604299 | No effect |
| Palomero Toluqueno | R1-13P-Q | AS365 | OTU8 | *Erwinia* | *aphidicola* | 99 | 99.79% | MW370211 | light clearing |
| Palomero Toluqueno | R1-13P-P | AS-391 | OTU50 | *Erwinia* | *psidii* | 99 | 93.99% | OL604398 | biofilm |
| Palomero Toluqueno | R1-13P-L | AS-364 | OTU76 | *Ficitibacilus* | *nanhaiensis* | 99 | 99.16% | OL604290 | No effect |
| Palomero Toluqueno | R1-13P-F | AS-337 | OTU77 | *Lactococcus* | *lactis* | 100 | 99.86% | OL604282 | No effect |
| Palomero Toluqueno | R1-13P-D | AS-335 | OTU16 | *Pantoea* | *agglomerans* | 100 | 99.01% | OL604391 | No effect |
| Palomero Toluqueno | R1-13P-H | AS339 | OTU69 | *Pantoea* | *agglomerans* | 95 | 96.72% | MW367619 | No effect |
| Palomero Toluqueno | R1-13P-N | AS342 | OTU17 | *Pantoea* | *agglomerans* | 99 | 99.01% | MW370193 | No effect |
| Palomero Toluqueno | R1-13P-R | AS366 | OTU16 | *Pantoea* | *agglomerans* | 99 | 98.56% | MW385275 |  |
| Palomero Toluqueno | R1-13P-I | AS-340 | OTU25 | *Pseudomonas* | *psychrotolerans* | 99 | 99.64% | OL604283 | Color change |
| Palomero Toluqueno | R1-13P-A | AS-334 | OTU46 | *Sphingomonas* | *sanguinis* | 100 | 99.33% | OL604281 | Color change |
| Palomero Toluqueno | R1-13P-G | AS338 | OTU65 | *Sphingomonas* | *roseiflava* | 99 | 99.71% | MW370190 | Color change |
| Palomero Toluqueno | R1-13P-K | AS-341 | OTU65 | *Sphingomonas* | *roseiflava* | 99 | 99.85% | OL604284 |  |
| Palomero Toluqueno | R1-13P-J | AS390 | OTU46 | *Sphingomonas* | *paucimobilis* | 99 | 99.24% | MW385276 | No effect |
| Vandeno | R1-15P-H | AS-248 | OTU51 | *Acinetobacter* | *guillouiae* | 99 | 99.42% | OL604262 | No effect |
| Vandeno | R1-15P-V | AS-264 | OTU78 | *Arenivirga* | *flava* | 100 | 99.40% | OL604268 |  |
| Vandeno | R1-15P-A3 | AS-217 | OTU52 | *Bacillus* | *pseudomycoides* | 100 | 99.79% | OL604248 | No effect |
| Vandeno | R1-15P-T | AS254 | OTU52 | *Bacillus* | *pseudomycoides* | 99 | 99.51% | MW369950 | No effect |
| Vandeno | R1-15P-I | AS249 | OTU72 | *Chryseobacterium* | *lactis* | 99 | 98.55% | MW369946 | color change |
| Vandeno | R1-15P-F | AS-258 | OTU72 | *Chryseobacterium* | *lactis* | 99 | 98.75% | OL604265 |  |
| Vandeno | R1-15P-W | AS-259 | OTU72 | *Chryseobacterium* | *candidae* | 100 | 98.93% | OL604382 | No effect |
| Vandeno | R1-15P-S | AS215 | OTU70 | *Enterobacter* | *tabaci* | 99 | 96.15% | MW367612 | clearing |
| Vandeno | R1-15P-B | AS243 | OTU8 | *Erwinia* | *aphidicola* | 97 | 98.34% | MW369940 | some overgrowth |
| Vandeno | R1-15P-X | AS255 | OTU79 | *Ochrobactrum* | *pituitosum* | 99 | 98.63% | MW369951 | No effect |
| Vandeno | R1-15P-A | AS-242 | OTU88 | *Paenibacillus* | *kyungheensis* | 100 | 98.94% | OL604261 | No effect |
| Vandeno | R1-15P-L | AS-211 | OTU42 | *Pantoea* | *brenneri* | 100 | 99.86% | OL604374 |  |
| Vandeno | R1-15P-P | AS213 | OTU6 | *Pantoea* | *ananatis* | 99 | 99.15% | MW369916 | No effect |
| Vandeno | R1-15P-Q | AS214 | OTU17 | *Pantoea* | *agglomerans* | 99 | 99.08% | MW369917 | No effect |
| Vandeno | R1-15P-K | AS-250 | OTU68 | *Pedobacter* | *terrae* | 100 | 99.12% | OL604263 | biofilm |
| Vandeno | R1-15P-N | AS-251 | OTU68 | *Pedobacter* | *suwonensis* | 100 | 99.07% | OL604264 |  |
| Vandeno | R1-15P-D | AS245 | OTU73 | *Plantibacter* | *flavus* | 99 | 99.50% | MW369942 | No effect |
| Vandeno | R1-15P-R | AS-253 | OTU73 | *Plantibacter* | *flavus* | 99 | 99.39% | OL604380 | color change |
| Vandeno | R1-15P-M | AS212 | OTU62 | *Pseudomonas* | *helleri* | 99 | 99.43% | MW369915 | color change + light clearing |
| Vandeno | R1-15P-U | AS-216 | OTU20 | *Pseudomonas* | *lurida* | 100 | 99.48% | MW644748 | Biofilm interesting |
| Vandeno | R1-15P-E | AS246 | OTU20 | *Pseudomonas* | *lurida* | 99 | 99.29% | MW369943 | Biofilm interesting |
| Vandeno | R1-15P-G | AS247 | OTU54 | *Pseudomonas* | *congelans* | 100 | 99.64% | MW369944 | No effect |
| Vandeno | R1-15P-A2 | AS257 | OTU20 | *Pseudomonas* | *lurida* | 99 | 100.00% | MW369952 |  |
| Vandeno | R1-15P-O | AS252 | OTU10 | *Rahnella* | *aquatilis* | 99 | 99.01% | MW369949 | color change |
| Vandeno | R1-15P-C | AS244 | OTU63 | *Rhizobium* | *nepotum* | 100 | 99.63% | MW369941 | No effect |
| Vandeno | R1-15P-Y | AS-256 | OTU21 | *Stenotrophomonas* | *maltophilia* | 99 | 100.00% | OL604381 | No effect |
| Zapalote Chico | R1-14P-I | AS-222 | OTU35 | *Acidovorax* | *avenae* | 99 | 99.35% | OL604253 | No effect |
| Zapalote Chico | R1-14P-L | AS-196 | OTU85 | *Bacillus* | *aryabhattai* | 100 | 100.00% | OL604245 | No effect |
| Zapalote Chico | R1-14P-K | AS-223 | OTU14 | *Chryseobacterium* | *hagamense* | 99 | 98.78% | OL604254 | No effect |
| Zapalote Chico | R1-14P-C | AS-219 | OTU15 | *Microbacterium* | *testaceum* | 99 | 99.71% | OL604249 | No effect |
| Zapalote Chico | R1-14P-G | AS-220 | OTU15 | *Microbacterium* | *testaceum* | 100 | 99.85% | OL604251 |  |
| Zapalote Chico | R1-14P-H | AS-221 | OTU15 | *Microbacterium* | *testaceum* | 100 | 99.78% | OL604252 |  |
| Zapalote Chico | R1-14P-M | AS-224 | OTU15 | *Microbacterium* | *testaceum* | 100 | 99.48% | OL604255 |  |
| Zapalote Chico | R1-14P-Q | AS-227 | OTU61 | *Microbacterium* | *zeae* | 100 | 99.39% | OL604375 | No effect |
| Zapalote Chico | R1-14P-T | AS-229 | OTU15 | *Microbacterium* | *testaceum* | 99 | 99.78% | OL604256 |  |
| Zapalote Chico | R1-14P-V | AS-230 | OTU61 | *Microbacterium* | *zeae* | 100 | 99.51% | OL604376 |  |
| Zapalote Chico | R1-14P-A2 | AS-261 | OTU15 | *Microbacterium* | *testaceum* | 99 | 99.78% | OL604266 |  |
| Zapalote Chico | R1-14P-F | AS-266 | OTU74 | *Mucilaginibacter* | *galii* | 99 | 97.95% | OL604384 |  |
| Zapalote Chico | R1-14P-A | AS-193 | OTU16 | *Pantoea* | *agglomerans* | 100 | 99.31% | OL604366 | light clearing |
| Zapalote Chico | R1-14P-B | AS-194 | OTU16 | *Pantoea* | *ananatis* | 100 | 99.64% | OL604367 |  |
| Zapalote Chico | R1-14P-J | AS-195 | OTU16 | *Pantoea* | *ananatis* | 100 | 99.16% | OL604368 |  |
| Zapalote Chico | R1-14P-N | AS-197 | OTU16 | *Pantoea* | *ananatis* | 100 | 99.15% | OL604369 |  |
| Zapalote Chico | R1-14P-R | AS198 | OTU16 | *Pantoea* | *ananatis* | 100 | 99.17% | MW385268 | light clearing |
| Zapalote Chico | R1-14P-A5 | AS-235 | OTU16 | *Pantoea* | *ananatis* | 100 | 99.64% | OL604377 |  |
| Zapalote Chico | R1-14P-Z | AS-234 | OTU68 | *Pedobacter* | *suwonensis* | 99 | 98.98% | OL604258 | No effect |
| Zapalote Chico | R1-14P-Y | AS233 | OTU80 | *Pseudarthrobacter* | *polychromogenes* | 99 | 98.85% | MW383964 | No effect |
| Zapalote Chico | R1-14P-X | AS-232 | OTU63 | *Rhizobium* | *nepotum* | 99 | 99.25% | OL604257 | No effect |
| Zapalote Chico | R1-14P-P | AS226 | OTU11 | *Rosenbergiella* | *epipactidis* | 99 | 99.58% | MW369927 | No effect |
| Zapalote Chico | R1-14P-W | AS231 | OTU46 | *Sphingomonas* | *paucimobilis* | 100 | 99.25% | MW385270 | No effect |
| Wild Mexicana | R1-2P-F | AS-411 | OTU84 | *Bacillus* | *metaterium* | 100 | 99.83% | OL604400 | No effect |
| Wild Mexicana | R1-2P-G | AS412 | OTU87 | *Bacillus* | *drentensis* | 98 | 99.42% | MW370241 | No effect |
| Wild Mexicana | R1-2P-L | AS414 | OTU53 | *Bacillus* | *simplex* | 99 | 99.67% | MW384871 | No effect |
| Wild Mexicana | R1-2P-I | AS-447 | OTU86 | *Bacillus* | *loiseleuriae* | 100 | 99.40% | OL604409 | No effect |
| Wild Mexicana | R1-2P-E | AS-536 | OTU4 | *Frigoribacterium* | *faeni* | 100 | 99.49% | OL604320 | No effect |
| Wild Mexicana | R1-2P-C | AS-537 | OTU59 | *Massilia* | *niabensis* | 99 | 98.47% | OL604321 |  |
| Wild Mexicana | R1-2P-A | AS-443 | OTU5 | *Microbacterium* | *zeae* | 100 | 100.00% | OL604408 | No effect |
| Wild Mexicana | R1-2P-K | AS-413 | OTU16 | *Pantoea* | *ananatis* | 100 | 99.55% | OL604401 |  |
| Wild Mexicana | R1-2P-B | AS444 | OTU65 | *Sphingomonas* | *roseiflava* | 100 | 99.78% | MW370259 | no effect |
| Wild Mexicana | R1-2P-D | AS445 | OTU65 | *Sphingomonas* | *roseiflava* | 99 | 99.78% | MW370260 |  |
| Wild Parviglumis | R1-1P2020-E | AS-544 | OTU13 | *Acinetobacter* | *baylyi* | 99 | 99.64% | OL604324 |  |
| Wild Parviglumis | R1-1P2020-I | AS547 | OTU83 | *Acinetobacter* | *calcoaceticus* | 99 | 99.71% | MW370143 | No effect |
| Wild Parviglumis | R1-1P2020-J | AS548 | OTU13 | *Acinetobacter* | *baylyi* | 99 | 99.50% | MW370144 | light clearing |
| Wild Parviglumis | R1-1P2020-O | AS-550 | OTU13 | *Acinetobacter* | *baylyi* | 99 | 99.71% | OL604326 |  |
| Wild Parviglumis | R1-1P2020-Q | AS361 | OTU13 | *Acinetobacter* | *baylyi* | 100 | 99.71% | OL604327 |  |
| Wild Parviglumis | R1-1P-H | AS425 | OTU85 | *Bacillus* | *aryabhattai* | 99 | 100.00% | MW383969 |  |
| Wild Parviglumis | R1-1P-L | AS426 | OTU85 | *Bacillus* | *aryabhattai* | 100 | 99.86% | MW370250 | No effect |
| Wild Parviglumis | R1-1P-M | AS-427 | OTU52 | *Bacillus* | *pseudomycoides* | 100 | 100.00% | OL604304 | No effect |
| Wild Parviglumis | R1-1P-R | AS-428 | OTU53 | *Bacillus* | *simplex* | 99 | 99.86% | OL604305 | No effect |
| Wild Parviglumis | R1-1P-P | AS440 | OTU86 | *Bacillus* | *licheniformis* | 100 | 99.65% | MW370256 | No effect |
| Wild Parviglumis | R1-1P-Q | AS-441 | OTU39 | *Bacillus* | *soli* | 99 | 98.34% | OL604307 |  |
| Wild Parviglumis | R1-1P-Z | AS477 | OTU64 | *Brevundimonas* | *vesicularis* | 99 | 99.85% | MW370089 | No effect |
| Wild Parviglumis | R1-1P2020-G | AS545 | OTU14 | *Chryseobacterium* | *hagamense* | 99 | 98.77% | MW370141 | No effect |
| Wild Parviglumis | R1-1P2020-R | AS561 | OTU14 | *Chryseobacterium* | *hagamense* | 99 | 98.84% | MW370153 |  |
| Wild Parviglumis | R1-1P-N | AS-438 | OTU23 | *Deinococcus* | *arenae* | 100 | 99.80% | OL604406 | No effect |
| Wild Parviglumis | R1-1P2020-V | AS-556 | OTU71 | *Enterobacter* | *kobei* | 99 | 99.26% | OL604418 | No effect |
| Wild Parviglumis | R1-1P-V | AS-430 | OTU8 | *Erwinia* | *aphidicola* | 100 | 100.00% | OL604405 | light clearing |
| Wild Parviglumis | R1-1P2020-A | AS540 | OTU8 | *Erwinia* | *aphidicola* | 99 | 99.36% | MW370136 | No effect |
| Wild Parviglumis | R1-1P-U | AS429 | OTU81 | *Exiguobacterium* | *sibiricum* | 100 | 99.57% | MW385279 | No effect |
| Wild Parviglumis | R1-1P-A | AS-471 | OTU82 | *Hymenobacter* | *gelipurpurascens* | 100 | 98.25% | OL604417 | No effect |
| Wild Parviglumis | R1-1P2020-B | AS541 | OTU67 | *Kluyvera* | *intermedia* | 99 | 98.37% | MW370137 | Strong inhibition |
| Wild Parviglumis | R1-1P2020-S | AS-553 | OTU67 | *Kluyvera* | *intermedia* | 99 | 98.36% | MW644743 | Minor inhibition |
| Wild Parviglumis | R1-1P2020-U | AS-555 | OTU67 | *Kluyvera* | *intermedia* | 99 | 98.41% | MW644744 | Minor inhibition |
| Wild Parviglumis | R1-1P2020-N | AS560 | OTU67 | *Kluyvera* | *intermedia* | 99 | 98.30% | MW370152 |  |
| Wild Parviglumis | R1-1P-Y | AS481 | OTU31 | *Kocuria* | *koreensis* | 99 | 99.07% | MW370091 | No effect |
| Wild Parviglumis | R1-1P-B | AS431 | OTU61 | *Microbacterium* | *zeae* | 100 | 99.58% | MW385280 | No effect |
| Wild Parviglumis | R1-1P-O | AS-439 | OTU5 | *Microbacterium* | *zeae* | 100 | 99.54% | OL604407 | No effect |
| Wild Parviglumis | R1-1P2020-F | AS-549 | OTU5 | *Microbacterium* | *kyungheense* | 99 | 98.77% | OL604325 |  |
| Wild Parviglumis | R1-1P2020-K | AS-558 | OTU61 | *Microbacterium* | *kyungheense* | 100 | 99.58% | OL604420 |  |
| Wild Parviglumis | R1-1P-C | AS-423 | OTU16 | *Pantoea* | *agglomerans* | 100 | 99.27% | OL604404 | No effect |
| Wild Parviglumis | R1-1P-F | AS424 | OTU17 | *Pantoea* | *agglomerans* | 100 | 94.74% | MW370249 |  |
| Wild Parviglumis | R1-1P2020-W | AS-557 | OTU16 | *Pantoea* | *anthophila* | 100 | 99.22% | OL604419 |  |
| Wild Parviglumis | R1-1P2020-D | AS-543 | OTU43 | *Pedobacter* | *terrae* | 100 | 99.48% | OL604323 | No effect |
| Wild Parviglumis | R1-1P2020-C | AS-542 | OTU54 | *Pseudomonas* | *cerasi* | 99 | 99.86% | MW644745 | Minor inhibition |
| Wild Parviglumis | R1-1P2020-H | AS546 | OTU20 | *Pseudomonas* | *lurida* | 99 | 99.72% | MW370142 | light clearing |
| Wild Parviglumis | R1-1P2020-M | AS-559 | OTU20 | *Pseudomonas* | *lurida* | 100 | 100.00% | OL604328 | color change |
| Wild Parviglumis | R1-1P-E | AS433 | OTU65 | *Sphingomonas* | *yabuuchiae* | 99 | 99.20% | MW370254 | No effect |
| Wild Parviglumis | R1-1P-J | AS436 | OTU47 | *Sphingomonas* | *panii* | 100 | 99.84% | MW385281 | No effect |
| Wild Parviglumis | R1-1P-W | AS566 | OTU66 | *Sphingomonas* | *aerolata* | 99 | 99.04% | MW370157 |  |
| Wild Parviglumis | R1-1P2020-P | AS551 | OTU21 | *Stenotrophomonas* | *tumulicola* | 99 | 99.76% | MW385289 | No effect |
| Wild Parviglumis | R1-1P2020-T | AS554 | OTU21 | *Stenotrophomonas* | *maltophilia* | 99 | 100.00% | MW385290 | No effect |

**Table S3.** Table showing pollen-associated bacteria that are unique to a host maize accession. ‘Red’ text denotes a unique bacterial genus, ‘green’ text denotes a unique species, and ‘blue’ text denotes a unique OTU with respect to the indicated host.

| **Host** | **Sample Name** | **Strain ID** | **Predicted Genus*** | **Predicted Species*** | **OTUs No.*** | **% Query cover** | **% identity** | **Accession number** | **Dual culture assay** |
| --- | --- | --- | --- | --- | --- | --- | --- | --- | --- |
| Camelia | R1-19P-J | AS289 | *Exiguobacterium* | *indicum* | OTU3 | 100 | 99.10% | MW369975 | No effect |
| Camelia | R1-19P-F | AS-296 | *Curtobacterium* | *pusillum* | OTU1 | 99 | 99.49% | OL604278 | No effect |
| Camelia | R1-19P-K | AS301 | *Curtobacterium* | *flaccumfaciens* | OTU1 | 99 | 99.42% | MW370160 |  |
| Canilla | R1-21P-B | AS-378 | *Rosenbergiella* | *collisarenosi* | OTU12 | 100 | 99.83% | OL604397 |  |
| Canilla | R1-21P-K | AS-564 | *Methylobacterium* | *cerastii* | OTU9 | 99 | 98.59% | OL604330 |  |
| Cateto Nortista | R1-22P-C | AS475 | *Pantoea* | *ananatis* | OTU19 | 100 | 96.12% | MW370087 | No effect |
| Chapalote | R1-4P-B | AS344 | *Pseudomonas* | *fulva* | OTU24 | 100 | 99.64% | MW383967 | color change |
| Chapalote | R1-4P-E | AS347 | *Aureimonas* | *ureilytica* | OTU22 | 99 | 99.19% | MW370196 | No effect |
| Chapalote | R1-4P-M | AS354 | *Siphonobacter* | *intestinalis* | OTU26 | 100 | 96.08% | MW370201 | color change |
| Confite Morocho | R1-25P-A | AS-451 | *Staphylococcus* | *xylosus* | OTU32 | 100 | 99.52% | OL604410 |  |
| Confite Morocho | R1-25P-C | AS453 | *Staphylococcus* | *kloosii* | OTU33 | 100 | 100.00% | MW385282 |  |
| Confite Morocho | R1-25P-D | AS454 | *Brachybacterium* | *squillarum* | OTU28 | 99 | 96.34% | MW370067 | No effect |
| Confite Morocho | R1-25P-F | AS-455 | *Staphylococcus* | *lentus* | OTU34 | 100 | 100.00% | OL604308 |  |
| Confite Morocho | R1-25P-J | AS457 | *Pseudomonas* | *cremoricolorata* | OTU24 | 98 | 99.57% | MW370070 | color change |
| Confite Morocho | R1-25P-M | AS-459 | *Deinococcus* | *radiotolerans* | OTU30 | 100 | 99.58% | OL604412 | No effect |
| Confite Morocho | R1-25P-P | AS478 | *Brachybacterium* | *squillarum* | OTU29 | 99 | 97.26% | MW370090 |  |
| Confite Morocho | R1-25P-R | AS486 | *Bacillus* | *wiedmannii* | OTU27 | 99 | 99.65% | MW370093 | light clearing |
| Confite Morocho | R1-25P-E | AS-488 | *Bacillus* | *proteolyticus* | OTU27 | 100 | 99.71% | OL604316 |  |
| Cristalino de Chihuahua | L1-7P-J | AS-10 | *Microbacterium* | *testaceum* | OTU41 | 99 | 99.20% | OL604243 | biofilm |
| Cristalino de Chihuahua | L1-7P-O | AS-14 | *Agrobacterium* | *larrymoorei* | OTU36 | 100 | 99.85% | OL604244 | No effect |
| Cristalino de Chihuahua | L1-7P-R | AS17 | *Pseudomonas* | *oryzihabitans* | OTU44 | 99 | 99.79% | MW369766 | color change |
| Cristalino de Chihuahua | L1-7P-B | AS2 | *Staphylococcus* | *sciuri* | OTU34 | 99 | 99.79% | MW349704 | No effect |
| Cristalino de Chihuahua | L1-7P-V | AS20 | *Bacillus* | *pumilus* | OTU39 | 100 | 99.44% | MW369768 | No effect |
| Cristalino de Chihuahua | R1-7P-E | AS-25 | *Bacillus* | *toyonensis/thuringiensis* | OTU27 | 100 | 100.00% | MW644736 | Strong inhibition |
| Cristalino de Chihuahua | L1-7P-K | AS-269 | *Streptomyces* | *coelescens* | OTU48 | 100 | 99.92% | OL604269 | No effect |
| Cristalino de Chihuahua | R1-7P-L | AS-30 | *Pseudomonas* | *psychrotolerans* | OTU45 | 100 | 99.42% | OL604279 |  |
| Cristalino de Chihuahua | R1-7P-U | AS-39 | *Pantoea* | *allii* | OTU6 | 99 | 99.78% | OL604298 | color change |
| Cristalino de Chihuahua | R1-7P-A3 | AS49 | *Azospirillum* | *melinis* | OTU38 | 99 | 97.73% | MW369788 |  |
| Cristalino de Chihuahua | L1-7P-F | AS-6 | *Lysinibacillus* | *pakistanensis* | OTU40 | 99 | 99.14% | OL604331 | light clearing |
| Cristalino de Chihuahua | R1-7P-D | AS64 | *Atlantibacter* | *hermannii* | OTU37 | 100 | 94.21% | MW383958 | No effect |
| Dente Branco | R1-18P-K | AS-207 | *Erwinia* | *persicina* | OTU50 | 100 | 99.76% | OL604373 |  |
| Dente Branco | R1-18P-F | AS-262 | *Chryseobacterium* | *hagamense* | OTU49 | 100 | 99.01% | OL604383 |  |
| Jala | R1-9P-A | AS-355 | *Serratia* | *liquefaciens* | OTU55 | 100 | 99.77% | OL604395 | No effect |
| Jala | R1-9P-B | AS-356 | *Pseudomonas* | *extremorientalis* | OTU54 | 99 | 99.78% | OL604286 | color change |
| Jala | R1-9P-C | AS357 | *Pseudomonas* | *syringae* | OTU24 | 99 | 99.64% | MW370204 | No effect |
| Jala | R1-9P-K | AS-360 | *Serratia* | *marcescens* | OTU56 | 99 | 99.86% | OL604288 | light clearing |
| Jala | R1-9P-L | AS-361 | *Serratia* | *ficaria* | OTU57 | 100 | 98.99% | MW644737 | Minor inhibition |
| Kulli | R1-24P-P | AS-483 | *Arthrobacter* | *pascens* | OTU58 | 100 | 99.48% | OL604315 | light clearing |
| Kulli | R1-24P-C | AS-562 | *Massilia* | *brevitalea* | OTU59 | 99 | 98.50% | OL604329 | No effect |
| Kulli | R1-24P-S | AS-569 | *Methylobacterium* | *indicum* | OTU60 | 99 | 94.84% | OL604421 | No effect |
| Nal-Tel | R1-10P-M | AS-539 | *Rathayibacter* | *festucae* | OTU75 | 99 | 99.86% | OL604322 |  |
| Oloton | R1-16P-G | AS-273 | *Erwinia* | *rhapontici* | OTU50 | 100 | 100.00% | OL604386 |  |
| Palomero Toluqueno | R1-13P-F | AS-337 | *Lactococcus* | *lactis* | OTU77 | 100 | 99.86% | OL604282 | No effect |
| Palomero Toluqueno | R1-13P-H | AS339 | *Pantoea* | *agglomerans* | OTU69 | 95 | 96.72% | MW367619 | No effect |
| Palomero Toluqueno | R1-13P-L | AS-364 | *Ficitibacilus* | *nanhaiensis* | OTU76 | 99 | 99.16% | OL604290 | No effect |
| Palomero Toluqueno | R1-13P-P | AS-391 | *Erwinia* | *psidii* | OTU50 | 99 | 93.99% | OL604398 | biofilm |
| Vandeno | R1-15P-L | AS-211 | *Pantoea* | *brenneri* | OTU42 | 100 | 99.86% | OL604374 |  |
| Vandeno | R1-15P-M | AS212 | *Pseudomonas* | *helleri* | OTU62 | 99 | 99.43% | MW369915 | color change + light clearing |
| Vandeno | R1-15P-S | AS215 | *Enterobacter* | *tabaci* | OTU70 | 99 | 96.15% | MW367612 | clearing |
| Vandeno | R1-15P-A | AS-242 | *Paenibacillus* | *kyungheensis* | OTU88 | 100 | 98.94% | OL604261 | No effect |
| Vandeno | R1-15P-G | AS247 | *Pseudomonas* | *congelans* | OTU54 | 100 | 99.64% | MW369944 | No effect |
| Vandeno | R1-15P-H | AS-248 | *Acinetobacter* | *guillouiae* | OTU51 | 99 | 99.42% | OL604262 | No effect |
| Vandeno | R1-15P-I | AS249 | *Chryseobacterium* | *lactis* | OTU72 | 99 | 98.55% | MW369946 | color change |
| Vandeno | R1-15P-R | AS-253 | *Plantibacter* | *flavus* | OTU73 | 99 | 99.39% | OL604380 | color change |
| Vandeno | R1-15P-X | AS255 | *Ochrobactrum* | *pituitosum* | OTU79 | 99 | 98.63% | MW369951 | No effect |
| Vandeno | R1-15P-W | AS-259 | *Chryseobacterium* | *candidae* | OTU72 | 100 | 98.93% | OL604382 | No effect |
| Vandeno | R1-15P-V | AS-264 | *Arenivirga* | *flava* | OTU78 | 100 | 99.40% | OL604268 |  |
| Zapalote Chico | R1-14P-Y | AS233 | *Pseudarthrobacter* | *polychromogenes* | OTU80 | 99 | 98.85% | MW383964 | No effect |
| Zapalote Chico | R1-14P-F | AS-266 | *Mucilaginibacter* | *galii* | OTU74 | 99 | 97.95% | OL604384 |  |
| Wild Mexicana | R1-2P-G | AS412 | *Bacillus* | *drentensis* | OTU87 | 98 | 99.42% | MW370241 | No effect |
| Wild Mexicana | R1-2P-I | AS-447 | *Bacillus* | *loiseleuriae* | OTU86 | 100 | 99.40% | OL604409 | No effect |
| Wild Mexicana | R1-2P-C | AS-537 | *Massilia* | *niabensis* | OTU59 | 99 | 98.47% | OL604321 |  |
| Wild Parviglumis | R1-1P-U | AS429 | *Exiguobacterium* | *sibiricum* | OTU81 | 100 | 99.57% | MW385279 | No effect |
| Wild Parviglumis | R1-1P-E | AS433 | *Sphingomonas* | *yabuuchiae* | OTU65 | 99 | 99.20% | MW370254 | No effect |
| Wild Parviglumis | R1-1P-P | AS440 | *Bacillus* | *licheniformis* | OTU86 | 100 | 99.65% | MW370256 | No effect |
| Wild Parviglumis | R1-1P-Q | AS-441 | *Bacillus* | *soli* | OTU39 | 99 | 98.34% | OL604307 |  |
| Wild Parviglumis | R1-1P-A | AS-471 | *Hymenobacter* | *gelipurpurascens* | OTU82 | 100 | 98.25% | OL604417 | No effect |
| Wild Parviglumis | R1-1P2020-C | AS-542 | *Pseudomonas* | *cerasi* | OTU54 | 99 | 99.86% | MW644745 | Minor inhibition |
| Wild Parviglumis | R1-1P2020-I | AS547 | *Acinetobacter* | *calcoaceticus* | OTU83 | 99 | 99.71% | MW370143 | No effect |
| Wild Parviglumis | R1-1P2020-V | AS-556 | *Enterobacter* | *kobei* | OTU71 | 99 | 99.26% | OL604418 | No effect |
| Wild Parviglumis | R1-1P2020-K | AS-558 | *Microbacterium* | *kyungheense* | OTU61 | 100 | 99.58% | OL604420 |  |
| Wild Parviglumis | R1-1P-W | AS566 | *Sphingomonas* | *aerolata* | OTU66 | 99 | 99.04% | MW370157 |  |

Table S4. Results of detailed literature searches of the pollen-associated bacterial species in terms of their pathogenicity and beneficial traits in maize and other plant species hosts. The OTUs and predicted species listed here are the ones that were conserved in two or more host accessions.

| **OTUs** | **Predicted species** | **Known maize pathogens** | **Known beneficial functions in maize** | **Known beneficial functions in other plants** |
| --- | --- | --- | --- | --- |
| OTU35 | *Acidovorax avenae* (99.35%) | Causes bacterial leaf blight in corn (Pataky et al., 1997)(Munkvold and White, 2016) | NA | Suppressed bacterial fruit blotch disease of watermelon (Krittidetch et al., 2013). |
| OTU13 | *Acinetobacter baylyi* (99.71%) |  | NA | Increased grain yield, and biomass production in black gram under stress (Yasin et al., 2018). |
| OTU51 | *Acinetobacter soli* (100%) |  | NA | Increased plant growth in *Erythrina brucei* under greenhouse conditions (Berza Beyene et al., 2022). |
| OTU85 | *Bacillus aryabhattai* (100%) |  | Promoted plant growth in maize (Marag and Suman, 2018). | Have phosphate-solubilizing, nitrogen fixation (Liu et al., 2006); and stress tolerance properties and enhanced the growth of soybean (Park et al., 2017). |
| OTU86 | *Bacillus licheniformis* (99.65%) |  | Maize seed endophyte (Marag and Suman, 2018); Biocontrol agent against *Exerohilum turcicum*, causing Turcicum leaf blight in maize (Bacon and Hinton, 2007); suppressed *Aspergillus flavus* and reduced aflatoxin accumulation in harvested maize grains (Rao et al., 2017; Hassan et al., 2019). | Increase plant growth and fresh weight in *Arabidopsis thaliana* (Bokhari, 2018). |
| OTU84 | *Bacillus metaterium* (99.51%) |  | Inhibited growth and mycotoxin biosynthesis of *A. flavus* and *F. verticillioides* (Saleh et al., 2021) and *F. moniliforme* (Fu et al., 2015) in maize suppressing ear rot diseases. | Showed antifungal activity against *Aspergillus flavus,* along with a reduction of aflatoxin production on stored rice grains (Mannaa et al., 2017). |
| OTU52 | *Bacillus pseudomycoides* (99.44%) |  | NA | Showed strong inhibition against *F. graminearum, F. proliferatum*, and *F. oxysporum* in alfalfa (Knežević et al., 2021); antagonism against *Fomes lamenensis* and *Ralstonia solani in vitro* (Vandana et al., 2018); controlled *F.oxysporum* and *Ralstonia syzigii* in chili pepper (Yanti et al., 2018); and delayed Fusarium infection in banana (Hadi et al., 2021) . |
| OTU39 | *Bacillus pumilus* (99.44%) |  | Inhibited *Aspergillus carbonarius* and reduced ochratoxin A contamination in maize kernels. | Biocontrol of *Rizoctonia solani* and *Fusarium oxysporum* in buckwheat (Agarwal et al., 2017); inhibited *A. flavus* and *A. terreus in vitro* (Bottone and Peluso, 2003). |
| OTU53 | *Bacillus simplex* (99.86%) |  | Increased yield in corn (Schwartz et al., 2013). | Controlled root rot disease caused by *F. camptoceras* in black cumin (Al-Sman et al., 2019) and *Phythium aphanidermatum*in tobacco (Miao et al., 2018); increased yield in soy and wheat (Senger et al., 2022). |
| OTU27 | *Bacillus toyonensis* (100%) |  | NA | Antagonistic against *F. graminearum*in dual culture assay (Saad et al., 2020); against *Verticillium dalhiae* and *F. oxysporum* in tomato causing Verticillium wilt disease (Essalimi et al., 2022); against *Rhizoctonia solani* in rice causing rice sheath blight (Khaskheli et al., 2020); against *Botrytis cinerea*  (Rojas-Solis et al., 2020). |
| OTU64 | *Brevundimonas vesicularis* (99.92%) |  | Maize seeds inoculated with *B. vesicularis* increased maize yield in field trials (Breedt et al., 2017). | Plant growth promotion with IAA production and nitrogen fixation *in vitro* (Liaqat and Eltem, 2016); can degrade herbicides 2,4,-D butyric acid which is a highly toxic contaminant (Dargahi et al., 2021). |
| OTU14 | *Chryseobacterium hagamense* (98.91%) |  | NA | NA |
| OTU23 | *Deinococcus arenae* (99.71%) |  | NA | Novel species isolated from sand in South Korea (Lee et al., 2016). |
| OTU18 | *Enterobacter ludwigii* (99.72) |  | Inoculation of maize with *E. ludwigii* along with PGPB enhanced plant resistance and growth (Wang et al., 2018) ; and increased maize yield (Fitriatin et al., 2021). | Promoted growth of wheat under stressed conditions (Ludueña et al., 2019); rice (Lee et al., 2019); and barley (Ribaudo et al., 2020); suppressed *Fusarium solani* in *Lolium perenne* grass (Shoebitz et al., 2009); and *Bursaphelenchus xylophilus* causing pine wilt diseases (Zhao et al., 2022). |
| OTU8 | *Erwinia aphidicola* (99.79%) |  | NA | NA |
| OTU2 | *Erwinia gerundensis* (98.89%) |  | NA | Suppressed *Fusarium tricinctum* and reduced the production of enniatins, a mycotoxin in barley (Gnonlonfoun et al., 2022). |
| OTU50 | *Erwinia rhapontici* (100%) |  | NA | NA |
| OTU3 | *Exiguobacterium acetylicum* (99.64%) |  | NA | Improved germination and growth in peas under pot culture conditions and inhibited *Rhizoctonia solani, Sclerotium rolfsii, Pythium,* and *F. oxysporium in vitro* (Selvakumar et al., 2009); *E. coli* and *B. subtilis* (Ranganathan et al., 2018)); controlled litchi downy blight caused by *Peronophythora litchii* (Zheng et al., 2019). |
| OTU4 | *Frigoribacterium faeni* (99.49%) |  | Increased root elongation and biomass of maize seedlings grown under metal-degraded soil contaminated with cadmium and zinc (Pereira and Castro, 2014). |  |
| OTU67 | *Kluyvera intermedia* (98.41%) |  | NA | Antagonistic *Rhizoctonia solani in vitro* (Anak et al., 2021); against *Pseudomonas syringae* pv. *actinidiae* causing bacterial canker disease in kiwifruit (Tontou et al., 2016); increased nitrogen and phosphorus uptake in wheat (Vazquez et al., 2000). |
| OTU31 | *Kocuria koreensis* (99.79%) |  | NA |  |
| OTU59 | *Massilia brevitalea* (98.50%) |  | NA | Novel bacterium isolated from lysimeter soil (Zul et al., 2008). |
| OTU15 | *Microbacterium testaceum* (99.78%) |  | A known maize endophyte (Zinniel et al., 2008). | Suppressed *A. flavus* and reduced aflatoxin production on stored rice grains (Mannaa et al., 2017); significantly reduced disease severity of coffee leaf rust (Silva et al., 2012) ; suppressed disease in potatoes caused by *Pectobacterium carotovorum* (Legein et al., 2020) ; enhanced growth in rice (Borah et al., 2018) . |
| OTU61 | *Microbacterium zeae* (99.58%) |  | A novel endophytic strain isolated from maize stem (Gao et al., 2017) . | NA |
| OTU5 | *Microbacterium zeae* (99.93%) |  |  |  |
| OTU7 | *Pantoea agglomerans* (97.46%) | Caused leaf blight and vascular wilt (Morales-Valenzuela et al., 2007) and dry stalk rot in maize (HuiYing et al., 2011) (. | Known as maize seed endophytes (Johnston-Monje and Raizada, 2011); enhanced growth of maize in saline soil (Gond et al., 2015). | Suppressed a broad range of fungal and bacterial pathogens including *Pyricularia grisea* in rice (Kim and Lee, 2019) ; *Erwinia amylovora* in apple and pear (Pusey et al., 2008) ; and suppressing bacterial wilt in beans (Hsieh et al., 2005); and promoted growth in sugarcane (Quecine et al., 2012); in rice (Feng et al., 2006) . |
| OTU17 | *Pantoea agglomerans* (99.72%) |  |  |  |
| OTU6 | *Pantoea ananatis* (99.36%) | Caused white spot disease in maize leaves (Paccola-Meirelles et al., 2001). | Increased growth in maize (Sheibani-Tezerji et al., 2015); inhibited maize-associated fungal pathogen *Lecanicillium aphanocladii in vitro* (Rijavec et al., 2007). | Reduced *Fg* DON mycotoxin in wheat heads (Deroo et al., 2022) ; suppressed rice blast disease caused by *Pyricularia oryzae* (Someya et al., 2003); increased plant growth and yield in rice (Megías et al., 2016) (Megías et al., 2017); suppressed Botrytis disease in grapevine caused by *Botrytis cinerea* (Roca-Couso et al., 2021); have N-fixation ability and produced auxin, a trait associated with root growth (Johnston-Monje and Raizada, 2011). |
| OTU16 | *Pantoea ananatis* (99.64%) |  |  |  |
| OTU42 | *Pantoea ananatis* (99.75%) |  |  |  |
| OTU43 | *Pedobacter suwonensis* (99.41%) |  |  | Reported to be isolated from the rhizosphere of Chinese Cabbage (Kwon et al., 2007). |
| OTU68 | *Pedobacter terrae* (99.12%) |  | Endophytes of maize roots (Gao et al., 2017a) |  |
| OTU54 | *Pseudomonas cerasi* (99.86%) |  | NA | NA |
| OTU24 | *Pseudomonas fulva* (99.64%) |  | Antagonistic against *Fusarium graminearum* and *F. culmorum* both *in vitro* and *in vivo* (Adeniji et al., 20)(Adeniji and Babalola, 2022). | Suppressed root diseases caused by *Rhizoctonia solani* in pine (Pokojska-Burdziej et al., 2004). |
| OTU20 | *Pseudomonas lurida* (100%) |  | NA | Increased root and shoot dry weight, nodulation, nutrient uptake, pod yield, and nutrient content of pods in common bean (Mishra et al., 2013); promoted wheat seedlings growth (Selvakumar et al., 2011). |
| OTU25 | *Pseudomonas psychrotolerans* (99.64%) |  | Increased biomass and chlorophyll content in maize under salinity stress (Kubi et al., 2021). | Reduced root disease severity caused by *Fusarium oxysporum* and *Macrophomina phaseolina* as well as increased overall biomass in chickpeas (Muhammad et al., 2015) ; enhanced growth in rice by contributing to nitrogen fixation (Liu et al., 2017); enhanced cucumber growth under heavy metal stress conditions (Kang et al., 2020). |
| OTU10 | *Rahnella aquatilis* (99.36%) |  | NA | Antagonistic against *Magnaporthe oryzae* and *F. graminearum* in rice (Sun et al., 2020) ; against grapevine crown gall (Chen et al., 2007); apple fire blight (Abo-Elyousr et al., 2011)); fruit storage rots (Navarta et al., 2011). |
| OTU63 | *Rhizobium nepotum* (99.63%) |  | NA | A plant growth promoter (Qaralleh et al., 2022); apple root endophytes (Muresan, 2017); increased in plant growth and yield of black soybean (Herliana et al., 2019). |
| OTU11 | *Rosenbergiella epipactidis* (100%) |  | NA | Endophytic bacterium isolated from capsicum (Gupta and Buch, 2020); from floral nectar of *Linaris vulgaris* (Bartlewicz et al., 2016) (Lenaerts et al., 2014); antagonistic against *Erwinia amylovora in vitro* (Barbé et al., 2022). |
| OTU47 | *Sphingomonas panni* (99.19%) |  | NA | Increased root growth in *Arabidopsis thaliana* (Thijs et al., 2014). |
| OTU65 | *Sphingomonas roseiflava* (99.85%) |  | NA | The function of this species is unclear (Hu et al., 2022); reported to be a novel species isolated from the ears of plants belonging to the family Gramineae (Yun et al., 2000). |
| OTU46 | *Sphingomonas sanguinis* (99.33%) |  | NA | Reported as rice seed endophyte (Midha et al., 2016); an endophyte of morning glory (Kim et al., 2021); suppressed bacterial speck and spot disease in tomatoes (Mbega et al., 2016). |
| OTU34 | *Staphylococcus lentus* (100%) |  | NA | Promoted seed germination rate in wheat, chickpea, and rice (Kamrun Nahar et al., 2022); enhanced the growth of wheat and sunflower under chromium-stressed conditions (Jamil et al., 2022). |
| OTU33 | *Staphylococcus xylosus*(99.7%) |  | NA | NA |
| OTU21 | *Stenotrophomonas maltophilia* (100%) |  | Enhanced maize plant growth and biomass in copper-stressed soil (Gopi et al., 2020); showed early growth in maize plants(Sang-Eun et al., 2005); increased root and shoot growth under saline stressed conditions (Naz and Bano, 2012). | Inhibited the growth of *Pythium ultimum in vitro* (Dunne et al., 1997) ; *Rhizoctonia solani,* and *Verticillium dahlia* (Berg et al., 1996); suppressed anthracnose in strawberries in the greenhouse (Alijani et al., 2020); *R. solanacearum* causing wilt disease in potato (Elhalag et al., 2015). Additionally, it promoted rice growth under metal-stressed soil (Zhou et al., 2020) ; and increased tomato seedling growth (Alijani et al., 2020). |

Table S5. SAS statistical output results of kernel disease severity for Score 4 (most diseased) and Score 1 (no disease symptoms)

**Table S5A.1. Greenhouse Trial 1 Kernel disease severity scoring SAS statistical output**

| **Greenhouse Trial 1 Kernel disease severity at the individual kernel level (Treatment Least Squares Means): For Score 4** | | | | | | | | |
| --- | --- | --- | --- | --- | --- | --- | --- | --- |
| **Trmt** | **Estimate** | **St. error** | **DF** | **t Value** | **Pr > \|t\|** | **Mean** | **SEM** | **TK Letters** |
| AS25 | -1.4483 | 0.2705 | 108 | -5.35 | <.0001 | 0.1903 | 0.04167 | c |
| AS404 | -0.1227 | 0.2698 | 108 | -0.45 | 0.6501 | 0.4694 | 0.06719 | b |
| AS541 | -1.8799 | 0.2710 | 108 | -6.94 | <.0001 | 0.1324 | 0.03113 | d |
| LB Control | 1.0763 | 0.2702 | 108 | 3.98 | 0.0001 | 0.7458 | 0.05122 | a |
| Proline PC | -6.9268 | 0.4639 | 108 | -14.93 | <.0001 | 0.000980 | 0.000454 | e |

**Table S5A.2. Greenhouse Trial 1 Kernel disease severity multiple comparisons SAS output**

| **Greenhouse Trial 1 Kernel disease severity differences of trmt Least Square Means Adjustment for Multiple Comparisons: Tukey-Kramer: For Score 4** | | | | | | | |
| --- | --- | --- | --- | --- | --- | --- | --- |
| **Trmt** | **_trmt** | **Estimate** | **St. error** | **DF** | **t Value** | **Pr > \|t\|** | **Adj P** |
| AS25 | AS404 | -1.3256 | 0.04628 | 108 | -28.64 | <.0001 | <.0001 |
| AS25 | AS541 | 0.4316 | 0.05207 | 108 | 8.29 | <.0001 | <.0001 |
| AS25 | LB Control | -2.5246 | 0.04880 | 108 | -51.74 | <.0001 | <.0001 |
| AS25 | Proline PC | 5.4785 | 0.3801 | 108 | 14.41 | <.0001 | <.0001 |
| AS404 | AS541 | 1.7572 | 0.04894 | 108 | 35.90 | <.0001 | <.0001 |
| AS404 | LB Control | -1.1990 | 0.04386 | 108 | -27.34 | <.0001 | <.0001 |
| AS404 | Proline PC | 6.8041 | 0.3798 | 108 | 17.92 | <.0001 | <.0001 |
| AS541 | LB Control | -2.9562 | 0.05137 | 108 | -57.55 | <.0001 | <.0001 |
| AS541 | Proline PC | 5.0469 | 0.3804 | 108 | 13.27 | <.0001 | <.0001 |
| LB Control | Proline PC | 8.0031 | 0.3801 | 108 | 21.05 | <.0001 | <.0001 |

**Table S5A.3. Greenhouse Trial 1 Kernel disease severity scoring SAS statistical output**

| **Greenhouse Trial 1 Kernel disease severity at the individual kernel level (Treatment Least Squares Means): For Score 1** | | | | | | | | |
| --- | --- | --- | --- | --- | --- | --- | --- | --- |
| **Trmt** | **Estimate** | **St. error** | **DF** | **t Value** | **Pr > \|t\|** | **Mean** | **SEM** | **TK Letters** |
| AS25 | 0.8617 | 0.3002 | 108 | 2.87 | 0.0049 | 0.7030 | 0.06269 | c |
| AS404 | -0.6066 | 0.3002 | 108 | -2.02 | 0.0458 | 0.3528 | 0.06854 | d |
| AS541 | 1.1484 | 0.3003 | 108 | 3.82 | 0.0002 | 0.7592 | 0.05490 | b |
| LB Control | -2.2670 | 0.3022 | 108 | -7.50 | <.0001 | 0.09389 | 0.02571 | e |
| Proline PC | 3.1353 | 0.3049 | 108 | 10.28 | <.0001 | 0.9583 | 0.01218 | a |

**Table S5A.4. Greenhouse Trial 1 Kernel disease severity multiple comparisons SAS output**

| **Greenhouse Trial 1 Kernel disease severity differences of trmt Least Square Means Adjustment for Multiple Comparisons: Tukey-Kramer: For Score 1** | | | | | | | |
| --- | --- | --- | --- | --- | --- | --- | --- |
| **Trmt** | **_trmt** | **Estimate** | **St. error** | **DF** | **t Value** | **Pr > \|t\|** | **Adj P** |
| AS25 | AS404 | 1.4683 | 0.04372 | 108 | 33.58 | <.0001 | <.0001 |
| AS25 | AS541 | -0.2867 | 0.04413 | 108 | -6.50 | <.0001 | <.0001 |
| AS25 | LB Control | 3.1287 | 0.05617 | 108 | 55.70 | <.0001 | <.0001 |
| AS25 | Proline PC | -2.2737 | 0.06877 | 108 | -33.06 | <.0001 | <.0001 |
| AS404 | AS541 | -1.7550 | 0.04421 | 108 | -39.69 | <.0001 | <.0001 |
| AS404 | LB Control | 1.6604 | 0.05488 | 108 | 30.25 | <.0001 | <.0001 |
| AS404 | Proline PC | -3.7419 | 0.06954 | 108 | -53.81 | <.0001 | <.0001 |
| AS541 | LB Control | 3.4154 | 0.05657 | 108 | 60.38 | <.0001 | <.0001 |
| AS541 | Proline PC | -1.9869 | 0.06878 | 108 | -28.89 | <.0001 | <.0001 |
| LB Control | Proline PC | -5.4024 | 0.07815 | 108 | -69.13 | <.0001 | <.0001 |

**Table S5B.1. Greenhouse Trial 2 Kernel disease severity scoring SAS statistical output**

| **Greenhouse Trial 2 Kernel disease severity at the individual kernel level (Treatment Least Squares Means): For Score 4** | | | | | | | | |
| --- | --- | --- | --- | --- | --- | --- | --- | --- |
| **Trmt** | **Estimate** | **St. error** | **DF** | **t Value** | **Pr > \|t\|** | **Mean** | **SEM** | **TK Letters** |
| AS25 | -0.8462 | 0.2805 | 109 | -3.02 | 0.0032 | 0.3002 | 0.05893 | c |
| AS404 | -0.2208 | 0.2803 | 109 | -0.79 | 0.4325 | 0.4450 | 0.06922 | b |
| AS541 | -1.0769 | 0.2806 | 109 | -3.84 | 0.0002 | 0.2541 | 0.05319 | d |
| LBControl | 0.6914 | 0.2805 | 109 | 2.47 | 0.0152 | 0.6663 | 0.06236 | a |
| Proline | -6.4063 | 0.4018 | 109 | -15.95 | <.0001 | 0.001648 | 0.000661 | e |

**Table S5B.2. Greenhouse Trial 2 Kernel disease severity multiple comparisons SAS output**

| **Greenhouse Trial 2 Kernel disease severity differences of trmt Least Square Means Adjustment for Multiple Comparisons: Tukey-Kramer: For Score 4** | | | | | | | |
| --- | --- | --- | --- | --- | --- | --- | --- |
| **Trmt** | **_trmt** | **Estimate** | **St. error** | **DF** | **t Value** | **Pr > \|t\|** | **Adj P** |
| AS25 | AS404 | -0.6254 | 0.04065 | 109 | -15.38 | <.0001 | <.0001 |
| AS25 | AS541 | 0.2307 | 0.04275 | 109 | 5.40 | <.0001 | <.0001 |
| AS25 | LBControl | -1.5376 | 0.04214 | 109 | -36.49 | <.0001 | <.0001 |
| AS25 | Proline | 5.5601 | 0.2906 | 109 | 19.13 | <.0001 | <.0001 |
| AS404 | AS541 | 0.8561 | 0.04177 | 109 | 20.50 | <.0001 | <.0001 |
| AS404 | LBControl | -0.9123 | 0.04052 | 109 | -22.51 | <.0001 | <.0001 |
| AS404 | Proline | 6.1854 | 0.2905 | 109 | 21.29 | <.0001 | <.0001 |
| AS541 | LBControl | -1.7684 | 0.04320 | 109 | -40.93 | <.0001 | <.0001 |
| AS541 | Proline | 5.3293 | 0.2907 | 109 | 18.33 | <.0001 | <.0001 |
| LBControl | Proline | 7.0977 | 0.2908 | 109 | 24.41 | <.0001 | <.0001 |

**Table S5B.3. Greenhouse Trial 2 Kernel disease severity scoring SAS statistical output**

| **Greenhouse Trial 2 Kernel disease severity at the individual kernel level (Treatment Least Squares Means): For Score 1** | | | | | | | | |
| --- | --- | --- | --- | --- | --- | --- | --- | --- |
| **Trmt** | **Estimate** | **St. error** | **DF** | **t Value** | **Pr > \|t\|** | **Mean** | **SEM** | **TK Letters** |
| AS25 | -0.06014 | 0.2595 | 109 | -0.23 | 0.8172 | 0.4850 | 0.06482 | b |
| AS404 | -0.7785 | 0.2597 | 109 | -3.00 | 0.0034 | 0.3146 | 0.05600 | c |
| AS541 | -0.02803 | 0.2595 | 109 | -0.11 | 0.9142 | 0.4930 | 0.06486 | b |
| LBControl | -2.4562 | 0.2625 | 109 | -9.36 | <.0001 | 0.07899 | 0.01910 | d |
| Proline | 3.4867 | 0.2676 | 109 | 13.03 | <.0001 | 0.9703 | 0.007711 | a |

**Table S5B.4. Greenhouse Trial 2 disease severity multiple comparisons SAS output**

| **Greenhouse Trial 2 Kernel disease severity differences of trmt Least Square Means Adjustment for Multiple Comparisons: Tukey-Kramer: For Score 1** | | | | | | | |
| --- | --- | --- | --- | --- | --- | --- | --- |
| **Trmt** | **_trmt** | **Estimate** | **St. error** | **DF** | **t Value** | **Pr > \|t\|** | **Adj P** |
| AS25 | AS404 | 0.7183 | 0.04003 | 109 | 17.94 | <.0001 | <.0001 |
| AS25 | AS541 | -0.03211 | 0.03887 | 109 | -0.83 | 0.4106 | 0.9221 |
| AS25 | LBControl | 2.3960 | 0.05558 | 109 | 43.11 | <.0001 | <.0001 |
| AS25 | Proline | -3.5469 | 0.07620 | 109 | -46.55 | <.0001 | <.0001 |
| AS404 | AS541 | -0.7504 | 0.04001 | 109 | -18.76 | <.0001 | <.0001 |
| AS404 | LBControl | 1.6777 | 0.05593 | 109 | 30.00 | <.0001 | <.0001 |
| AS404 | Proline | -4.2652 | 0.07719 | 109 | -55.26 | <.0001 | <.0001 |
| AS541 | LBControl | 2.4281 | 0.05553 | 109 | 43.73 | <.0001 | <.0001 |
| AS541 | Proline | -3.5148 | 0.07624 | 109 | -46.10 | <.0001 | <.0001 |
| LBControl | Proline | -5.9429 | 0.08648 | 109 | -68.72 | <.0001 | <.0001 |

Table S6. Type III secretion system operons with their functional name encoded in the genomes of pollen-associated anti-*Fusarium* bacteria that were tested in the replicated greenhouse trials.

| **Type III secretion system operons** | **Pollen-associated bacteria** | | | | |
| --- | --- | --- | --- | --- | --- |
| **Functional name*** | **AS541**  **(*Kluyvera intermedia*)** | | | **AS25**  **(*Bacillus cereus* group)** | **AS404 (*Bacillus pseudomycoides*)** |
| Inner MS ring protein | SctJ |  |  | - | - |
| Minor export apparatus protein |  | YscR/EscR/HrcR | Flip | - | - |
| Minor export apparatus protein |  | YscS/EscS/HrcS |  | - | - |
| Minor export apparatus protein | SctT |  | FliR | FliR | - |
| Export apparatus switch protein |  |  | FlhB | FlhB | - |
| Major export apparatus protein |  | YscV/EscV/HrcV | FlhA | - | - |
| ATPase | SctN |  |  | - | - |
| Needle Filament protein | SctF |  |  | - | - |
| Inner rod protein | SctI |  |  | - | - |
| Gatekeeper | SctW |  |  | - | - |

**References:**

Abo-Elyousr, A. M. K., Sallam, M. A. A., and Zeller, W. (2011). Effect of Acibenzolar-S-methyl and *Rahnella aquatilis* (Ra39) on fire blight of apple plants. *Acta Hortic.* 896, 511–518. doi: 10.17660/ACTAHORTIC.2011.896.75.

Adeniji, A. A., Aremu, O. S., Toit Loots, D., and Babalola, O. O. (2020). *Pseudomonas fulva* HARBPS9.1: candidate anti-*Fusarium* agent in South Africa. *Eur. J. Plant Pathol.* 157, 767–781. doi: 10.1007/s10658-020-02035-4.

Adeniji, A. A., and Babalola, O. O. (2022). Evaluation of *Pseudomonas fulva* PS9.1 and *Bacillus velezensis* NWUMFkBS10.5 as candidate plant growth promoters during maize-*Fusarium* interaction. *Plants* 11, 324. doi: 10.3390/PLANTS11030324/S1.

Agarwal, M., Dheeman, S., Dubey, R. C., Kumar, P., Maheshwari, D. K., and Bajpai, V. K. (2017). Differential antagonistic responses of *Bacillus pumilus* MSUA3 against *Rhizoctonia solani* and *Fusarium oxysporum* causing fungal diseases in *Fagopyrum esculentum* Moench. *Microbiol. Res.* 205, 40–47. doi: 10.1016/J.MICRES.2017.08.012.

Al-Sman, K. M., Abo-Elyousr, K., Eraky, A., and El-Zawahry, A. (2019). Potential activities of *Bacillus simplex* as a biocontrol agent against root rot of *Nigella sativa* caused by *Fusarium camptoceras*. *Egypt. J. Biol. Pest Control* 29, 79. doi: 10.1186/s41938-019-0191-z.

Alijani, Z., Amini, J., Ashengroph, M., and Bahramnejad, B. (2020). Volatile compounds mediated effects of *Stenotrophomonas maltophilia* strain UN1512 in plant growth promotion and its potential for the biocontrol of *Colletotrichum nymphaeae*. *Physiol. Mol. Plant Pathol.* 112, 101555. doi: 10.1016/J.PMPP.2020.101555.

Anak, H., Figen Donmez, M., and Coruh, I. (2021). Biological control of *Rhizoctonia Solani* Kühn. with *Rhizobacteria* isolated from different soil and *Calligonum polygonoides* L. Subsp. Comosum (L’hér.). *J. Agric.* 4, 92–107. doi: 10.46876/ja.986625.

Bacon, C. W., and Hinton, D. M. (2007). Biocontrol science and technology potential for control of seedling blight of wheat caused by *Fusarium graminearum* and related species using the bacterial endophyte *Bacillus mojavensis*. *Biocontrol Sci. Technol.* 17, 81–94. doi: 10.1080/09583150600937006.

Barbé, S., Figàs-Segura, À., Benada, M., Navarro-Herrero, I., Sampaio, T. M., Biosca, E. G., et al. (2022). Plant-associated microbiota as a source of antagonistic bacteria against the phytopathogen *Erwinia amylovora*. *Environ. Microbiol. Rep.* 14, 559–569. doi: 10.1111/1758-2229.13064.

Bartlewicz, J., Lievens, B., Honnay, O., and Jacquemyn, H. (2016). Microbial diversity in the floral nectar of *Linaria vulgaris* along an urbanization gradient. *BMC Ecol.* 16, 18. doi: 10.1186/S12898-016-0072-1/FIGURES/7.

Berg, G., Marten, P., and Ballin, G. (1996). *Stenotrophomonas maltophilia* in the rhizosphere of oilseed rape — occurrence, characterization and interaction with phytopathogenic fungi. *Microbiol. Res.* 151, 19–27. doi: 10.1016/S0944-5013(96)80051-6.

Berza Beyene, B., Pagano, M. C., Vaiyapuri R, P., and Assefa Tuji, F. (2022). Microbial consortia inoculation of woody legume *Erythrina brucei* increases nodulation and shoot nitrogen and phosphorus under greenhouse conditions. *Biotechnol. Reports* 33, e00707. doi: 10.1016/J.BTRE.2022.E00707.

Bokhari, A. (2018). Unearthing *Bacillus* endophytes from desert plants that enhance growth of *Arabidopsis thaliana* under abiotic stress conditions. PhD Thesis, King Abdullah University of Science and Technology, Saudi Arabia.

Borah, M., Das, S., Baruah, H., Boro, R. C., Barooah, M., and Boro, R. C. (2018). Diversity of culturable endophytic bacteria from wild and cultivated rice showed potential plant growth promoting activities. *bioRxiv*, 310797. doi: 10.1101/310797.

Breedt, G., Labuschagne, N., and Coutinho, T. A. (2017). Seed treatment with selected plant growth-promoting rhizobacteria increases maize yield in the field. *Ann. Appl. Biol.* 171, 229–236. doi: 10.1111/AAB.12366.

Chen, F., Guo, Y. B., Wang, J. H., Li, J. Y., and Wang, H. M. (2007). Biological control of grape crown gall by *Rahnella aquatilis* HX2. *Plant Dis.* 91, 957–963. doi: 10.1094/PDIS-91-8-0957.

Dargahi, A., Shokoohi, R., Asgari, G., Ansari, A., Nematollahi, D., and Samarghandi, M. R. (2021). Moving-bed biofilm reactor combined with three-dimensional electrochemical pretreatment (MBBR–3DE) for 2,4-D herbicide treatment: application for real wastewater, improvement of biodegradability. *RSC Adv.* 11, 9608–9620. doi: 10.1039/D0RA10821A.

Deroo, W., De Troyer, L., Dumoulin, F., De Saeger, S., De Boevre, M., Vandenabeele, S., et al. (2022). A novel in planta enrichment method employing *Fusarium graminearum*-infected wheat spikes to select for competitive biocontrol bacteria. *Toxins (Basel).* 14, 222. doi: 10.3390/TOXINS14030222/S1.

Dunne, C., Crowley, J. J., Moënne-Loccoz, Y., Dowling, D. N., De Bruijn, F. J., and O’Gara, F. (1997). Biological control of *Pythium ultimum* by *Stenotrophomonas maltophilia* W81 is mediated by an extracellular proteolytic activity. *Microbiology* 143, 3921–3931. doi: 10.1099/00221287-143-12-3921.

Elhalag, K. M., Emara, H. M., Messiha, N. A. S., Elhadad, S. A., and Abdallah, S. A. (2015). The relation of different crop roots exudates to the survival and suppressive effect of *Stenotrophomonas maltophilia* (PD4560), biocontrol agent of bacterial wilt of potato. *J. Phytopathol.* 163, 829–840. doi: 10.1111/JPH.12381.

Essalimi, B., Esserti, S., Rifai, L. A., Koussa, T., Makroum, K., Belfaiza, M., et al. (2022). Enhancement of plant growth, acclimatization, salt stress tolerance and verticillium wilt disease resistance using plant growth-promoting rhizobacteria (PGPR) associated with plum trees (*Prunus domestica*). *Sci. Hortic. (Amsterdam).* 291, 110621. doi: 10.1016/J.SCIENTA.2021.110621.

Feng, Y., Shen, D., and Song, W. (2006). Rice endophyte *Pantoea agglomerans* YS19 promotes host plant growth and affects allocations of host photosynthates. *J. Appl. Microbiol.* 100, 938–945. doi: 10.1111/J.1365-2672.2006.02843.X.

Fitriatin, B. N., Dellaocto, D., Ambarita, M., Rochimi, M., and Simarmata, T. (2021). The role of Rhizobacterial inoculum and formulated soil amendment in improving soil chemical-biological properties, chlorophyll content and agronomic efficiency of maize under marginal soils. *Jordan J. Biol. Sci.* 14, 601–605.

Fu, R., Yu, F., Gu, Y., Xue, T., Guo, Y., Wang, Y., et al. (2015). Improvement of Antagonistic Activity of *Bacillus megaterium* MHT6 against *Fusarium moniliforme* using He-Ne Laser Irradiation. *Int. J. Agric. Biol. Int. J. Agric. Biol* 17, 1141–1148. doi: 10.17957/IJAB/15.0048.

Gao, J. L., Sun, P., Mao, X. J., Du, Y. L., Liu, B. Y., and Sun, J. G. (2017a). *Pedobacter zeae* sp. Nov., an endophytic bacterium isolated from maize root. *Int. J. Syst. Evol. Microbiol.* 67, 231–236. doi: 10.1099/IJSEM.0.001603/CITE/REFWORKS.

Gao, J. lian, Sun, P., Wang, X. ming, Lv, F. yang, and Sun, J. guang (2017b). *Microbacterium zeae* sp. nov., an endophytic bacterium isolated from maize stem. *Antonie van Leeuwenhoek, Int. J. Gen. Mol. Microbiol.* 110, 697–704. doi: 10.1007/S10482-017-0837-3/FIGURES/1.

Gnonlonfoun, E., Fotin, G., Risler, A., Elfassy, A., Schwebel, S., Schmitt, M., et al. (2022). Inhibition of the growth of *Fusarium tricinctum* and reduction of its enniatin production by *Erwinia gerundensis* isolated from barley kernels. *J. Am. Soc. Brew. Chem.* 81, 340–350. doi: 10.1080/03610470.2022.2041970.

Gond, S. K., Torres, M. S., Bergen, M. S., Helsel, Z., and White, J. F. (2015). Induction of salt tolerance and up-regulation of aquaporin genes in tropical corn by rhizobacterium *Pantoea agglomerans*. *Lett. Appl. Microbiol.* 60, 392–399. doi: 10.1111/lam.12385.

Gupta, V., and Buch, A. D. (2020). Isolation and characterization of bacteria associated with sunscald-affected *Capsicum annuum* L. *Charusat J. by Charotar Univ. Sci. Technol.* 2, 58–63. Available at: http://www.ncbi.nlm.nih.gov [Accessed December 15, 2022].

Hadi, A. E., Khalisha, A., Pambudi, A., and Effendi, Y. (2021). Potential of bacteria consortium as growth controller of pathogenic fungi *Fusarium oxysporum* F. sp. *cubense* (Foc). *IOP Conf. Ser. Earth Environ. Sci.* 637, 012029. doi: 10.1088/1755-1315/637/1/012029.

Hassan, Z. U., Al Thani, R., Alnaimi, H., Migheli, Q., and Jaoua, S. (2019). Investigation and application of *Bacillus licheniformis* volatile compounds for the biological control of toxigenic *Aspergillus* and *Penicillium* spp. *ACS Omega* 4, 17186–17193. doi: 10.1021/acsomega.9b01638.

Herliana, O., Harjoso, T., Anwar, A. H. S., Fauzi, A., Soedirman, J., Dr, J. L., et al. (2019). The effect of *Rhizobium* and N fertilizer on growth and yield of black soybean (*Glycine max* (L) Merril). *IOP Conf. Ser. Earth Environ. Sci.* 255, 012015. doi: 10.1088/1755-1315/255/1/012015.

Hsieh, T. F., Huang, H. C., and Erickson, R. S. (2005). Biological control of bacterial wilt of bean using a bacterial endophyte, *Pantoea agglomerans*. *J. Phytopathol.* 153, 608–614. doi: 10.1111/J.1439-0434.2005.01027.X.

Hu, H., Liu, Y., Huang, Y., Zhang, Z., and Tang, H. (2022). The leaf microbiome of tobacco plants across eight Chinese provinces. *Microorganisms* 10, 450. doi: 10.3390/MICROORGANISMS10020450/S1.

HuiYing, C., HongJie, L., ZhenDong, Z., and XiaoMing, W. (2011). Seed transmission of *Pantoea agglomerans*, causal agent of dry stalk rot, in maize. *Acta Phytophylacica Sin.* 38, 31–36.

Jamil, N., Hyder, S., Valipour, M., Yasir, M., Iqbal, R., Roy, R., et al. (2022). Evaluation of the bioremediation potential of *Staphlococcus lentus* inoculations of plants as a promising strategy used to attenuate chromium toxicity. *Sustainability* 14, 13056. doi: 10.3390/SU142013056.

Johnston-Monje, D., and Raizada, M. N. (2011). Conservation and diversity of seed associated endophytes in *Zea* across boundaries of evolution, ethnography and ecology. *PLoS One* 6, e20396. doi: 10.1371/journal.pone.0020396.

Kamrun Nahar, M., Nazmul Haque, M., Kumar Paul, G., Islam, S., Abu Saleh, M., Salah Uddin, M., et al. (2022). Bacteria isolated from cultivated soil after liming promote seed germination and seedling growth of crop plants. *Curr. Res. Biotechnol.* 4, 21–31. doi: 10.1016/J.CRBIOT.2021.12.001.

Khaskheli, M. A., Wu, L., Chen, G., Chen, L., Hussain, S., Song, D., et al. (2020). Isolation and characterization of root-associated bacterial endophytes and their biocontrol potential against major fungal phytopathogens of rice (*Oryza sativa* L.). *Pathogens* 9, 172. doi: 10.3390/PATHOGENS9030172.

Kim, H., and Lee, Y. H. (2019). The rice microbiome: A model platform for crop holobiome. *Phytobiomes J.* 4, 5–18. doi: 10.1094/PBIOMES-07-19-0035-RVW/ASSET/IMAGES/LARGE/PBIOMES-07-19-0035-RVWF2.JPEG.

Kim, J.-S., Choi, K. R., Eun, H., Heo, J., Kwon, S.-W., Ko, Y.-J., et al. (2021). Complete genome sequence of *Sphingomonas sanguinis* NP2-R2, a carotenoid producing bacterium, isolated from morning glory. *J. Microbiol.* 57, 238–241. Available at: https://www.dbpia.co.kr/journal/articleDetail?nodeId=NODE10616464 [Accessed December 15, 2022].

Knežević, M. M., Stajković-Srbinović, O. S., Assel, M., Milić, M. D., Mihajlovski, K. R., Delić, D. I., et al. (2021). The ability of a new strain of *Bacillus pseudomycoides* to improve the germination of alfalfa seeds in the presence of fungal infection or chromium. *Rhizosphere* 18, 100353. doi: 10.1016/J.RHISPH.2021.100353.

Krittidetch, A., Wilawan, C., and Dusit, A. (2013). Detection of *Acidovorax avenae* subsp. avenae in commercial corn seeds and its correlation with seedling transmission. *African J. Biotechnol.* 12, 6376–6381. doi: 10.5897/AJB2013.12965.

Kubi, H. A. A., Khan, M. A., Adhikari, A., Imran, M., Kang, S. M., Hamayun, M., et al. (2021). Silicon and plant growth-promoting rhizobacteria *Pseudomonas psychrotolerans* CS51 mitigates salt stress in *Zea mays* L. *Agriculture* 11, 272. doi: 10.3390/AGRICULTURE11030272.

Kwon, S. W., Kim, B. Y., Lee, K. H., Jang, K. Y., Seok, S. J., Kwon, J. S., et al. (2007). *Pedobacter suwonensis* sp. nov., isolated from the rhizosphere of Chinese cabbage (*Brassica campestris*). *Int. J. Syst. Evol. Microbiol.* 57, 480–484. doi: 10.1099/IJS.0.64196-0/CITE/REFWORKS.

Lee, D., Cha, S., Jang, J. H., and Seo, T. (2016). *Deinococcus arenae* sp. nov., a novel species isolated from sand in South Korea. *Antonie van Leeuwenhoek, Int. J. Gen. Mol. Microbiol.* 109, 1055–1062. doi: 10.1007/S10482-016-0705-6/FIGURES/1.

Lee, K. E., Adhikari, A., Kang, S. M., You, Y. H., Joo, G. J., Kim, J. H., et al. (2019). Isolation and characterization of the high silicate and phosphate solubilizing novel strain *Enterobacter ludwigii* GAK2 that promotes growth in rice plants. *Agronomy* 9, 144. doi: 10.3390/AGRONOMY9030144.

Legein, M., Smets, W., Vandenheuvel, D., Eilers, T., Muyshondt, B., Prinsen, E., et al. (2020). Modes of action of microbial biocontrol in the phyllosphere. *Front. Microbiol.* 11, 1619. doi: 10.3389/FMICB.2020.01619/BIBTEX.

Lenaerts, M., Álvarez-Pérez, S., de Vega, C., Van Assche, A., Johnson, S. D., Willems, K. A., et al. (2014). *Rosenbergiella australoborealis* sp. nov., *Rosenbergiella collisarenosi* sp. nov. and *Rosenbergiella epipactidis* sp. nov., three novel bacterial species isolated from floral nectar. *Syst. Appl. Microbiol.* 37, 402–411. doi: 10.1016/J.SYAPM.2014.03.002.

Liaqat, F., and Eltem, R. (2016). Identification and characterization of endophytic bacteria isolated from *in vitro* cultures of peach and pear rootstocks. *3 Biotech* 6, 120. doi: 10.1007/S13205-016-0442-6.

Liu, R., Zhang, Y., Chen, P., Lin, H., Ye, G., Wang, Z., et al. (2017). Genomic and phenotypic analyses of *Pseudomonas psychrotolerans* PRS08-11306 reveal a turnerbactin biosynthesis gene cluster that contributes to nitrogen fixation. *J. Biotechnol.* 253, 10–13. doi: 10.1016/J.JBIOTEC.2017.05.012.

Liu, X., Zhao, H., and Chen, S. (2006). Colonization of maize and rice plants by strain *Bacillus megaterium* C4. *Curr. Microbiol.* 52, 186–190. doi: 10.1007/S00284-005-0162-3/FIGURES/1.

Ludueña, L. M., Anzuay, M. S., Angelini, J. G., McIntosh, M., Becker, A., Rupp, O., et al. (2019). Genome sequence of the endophytic strain *Enterobacter* sp. J49, a potential biofertilizer for peanut and maize. *Genomics* 111, 913–920. doi: 10.1016/j.ygeno.2018.05.021.

Mannaa, M., Oh, J. Y., and Kim, K. D. (2017). Microbe-mediated control of *Aspergillus flavus* in stored rice grains with a focus on aflatoxin inhibition and biodegradation. *Ann. Appl. Biol.* 171, 376–392. doi: 10.1111/AAB.12381.

Marag, P. S., and Suman, A. (2018). Growth stage and tissue specific colonization of endophytic bacteria having plant growth promoting traits in hybrid and composite maize (*Zea mays* L.). *Microbiol. Res.* 214, 101–113. doi: 10.1016/J.MICRES.2018.05.016.

Mbega, E. R., Wulff, E. G., Mabagala, R. B., Adriko, J., Lund, O. S., and Mortensen, C. N. (2016). Xanthomonads and other yellow-pigmented *Xanthomonas*-like bacteria associated with tomato seeds in Tanzania. *African J. Biotechnol.* 11, 14297–14304. doi: 10.4314/ajb.v11i78.

Megías, E., Megías, M., Ollero, F. J., and Hungria, M. (2016). Draft genome sequence of *Pantoea ananatis* strain AMG521, a rice plant growth-promoting bacterial endophyte isolated from the Guadalquivir marshes in southern Spain. *Genome Announc.* 4, 1681–1696. doi: 10.1128/GENOMEA.01681-15.

Megías, E., Reis Junior, F. B., Ribeiro, R. A., Megías, M., Ollero, F. J., and Hungria, M. (2017). Genome sequence of *Pantoea* sp. strain 1.19, isolated from rice rhizosphere, with the capacity to promote growth of legumes and nonlegumes. *Genome Announc.* 5, e00707-17. doi: 10.1128/GENOMEA.00707-17.

Miao, G. peng, Han, J., Wang, C. run, Zhang, K. gui, and Wang, S. chang (2018). Growth inhibition and induction of systemic resistance against *Pythium aphanidermatum* by *Bacillus simplex* strain HS-2. *Biocontrol Sci. Technol.* 28, 1114–1127. doi: 10.1080/09583157.2018.1514585.

Midha, S., Bansal, K., Sharma, S., Kumar, N., Patil, P. P., Chaudhry, V., et al. (2016). Genomic resource of rice seed associated bacteria. *Front. Microbiol.* 6, 1551. doi: 10.3389/FMICB.2015.01551/BIBTEX.

Mishra, P. K., Bisht, S. C., Jeevanandan, K., Kumar, S., Bisht, J. K., and Bhatt, J. C. (2013). Synergistic effect of inoculating plant growth-promoting *Pseudomonas* spp. and *Rhizobium leguminosarum*-FB1 on growth and nutrient uptake of rajmash (*Phaseolus vulgaris* L.). *Arch. Agron. Soil Sci.* 60, 799–815. doi: 10.1080/03650340.2013.843773.

Morales-Valenzuela, G., Silva-Rojas, H. V., Ochoa-Martínez, D., Valadez-Moctezuma, E., Alarcón-Zúñiga, B., Zelaya-Molina, L. X., et al. (2007). First report of *Pantoea agglomerans* causing Leaf blight and Vascular wilt in maize and sorghum in Mexico. *Plant Dis.* 91, 1365.1. doi: 10.1094/PDIS-91-10-1365A.

Muhammad, I.-H., Khalid, R., Ashfaq, M., Inam-Ul-Haq, M., Tahir, M. I., Hayat, R., et al. (2015). Bioefficacy of rhizobacterial isolates against root infecting fungal pathogens of chickpea (*Cicer arietinum* L.). *J Plant Pathol Microbiol* S3, 011. doi: 10.4172/2157-7471.S3-011.

Munkvold, G. P., and White, D. G. eds. (2016). *Compendium of corn diseases, Fourth Edition*. Fourth. St. Paul, MN, U.S.A: The American Phytopathological Society doi: 10.1094/9780890544945.

Muresan, L. E. (2017). Cultivable bacterial and fungal endophytes from apple tissues and their potential for biological control of *Venturia inaequalis*. Masters thesis, University of Guelph, Canada.

Navarta, L. G., Calvo, J., Calvente, V., Benuzzi, D., and Sanz, M. I. (2011). Freezing and freeze-drying of the bacterium *Rahnella aquatilis* BNM 0523: study of protecting agents, rehydration media and freezing temperatures. *Lett. Appl. Microbiol.* 53, 565–571. doi: 10.1111/J.1472-765X.2011.03150.X.

Paccola-Meirelles, Ferreira, Meirelles, Marriel, and Casela (2001). Detection of a bacterium associated with a Leaf spot disease of maize in Brazil. *J. Phytopathol.* 149, 275–279. doi: 10.1046/J.1439-0434.2001.00614.X.

Park, Y. G., Mun, B. G., Kang, S. M., Hussain, A., Shahzad, R., Seo, C. W., et al. (2017). *Bacillus aryabhattai* SRB02 tolerates oxidative and nitrosative stress and promotes the growth of soybean by modulating the production of phytohormones. *PLoS One* 12, e0173203. doi: 10.1371/JOURNAL.PONE.0173203.

Pataky, J. K., Toit, L. J. Du, and Kerns, M. R. (1997). Bacterial leaf blight on shrunken-2 sweet corn. *Plant Dis.* 81, 1293-1298. doi: 10.1094/PDIS.1997.81.11.1293. doi: 10.1094/PDIS.1997.81.11.1293.

Pereira, S. I. A., and Castro, P. M. L. (2014). Diversity and characterization of culturable bacterial endophytes from *Zea mays* and their potential as plant growth-promoting agents in metal-degraded soils. *Environ. Sci. Pollut. Res.* 21, 14110–14123. doi: 10.1007/S11356-014-3309-6/TABLES/4.

Pokojska-Burdziej, A., Strzelczyk, E., Dahm, H., and Li, C. Y. (2004). Effect of endophytic bacterium *Pseudomonas fulva* on growth of pine seedlings (*Pinus sylvestris*) formation of mycorrhizae and protection against pathogens. *Phytopathology* 32, 33–47.

Pusey, P. L., Stockwell, V. O., and Rudell, D. R. (2008). Antibiosis and acidification by *Pantoea agglomerans* strain E325 may contribute to suppression of *Erwinia amylovora*. *Biol. Control* 98, 1136–1143. doi: 10.1094/PHYTO-98-10-1136.

Qaralleh, H., Khleifat, K. M., Hajleh, M. N. A., Al-Limoun, M. O., Alshawawreh, R., Magharbeh, M. K., et al. (2022). Plant growth-promoting *Rhizobium nepotum* phenol utilization: Characterization and kinetics. *J. Hunan Univ. Nat. Sci.* 49, 94–107. doi: 10.55463/ISSN.1674-2974.49.4.11.

Quecine, M. C., Araújo, W. L., Rossetto, P. B., Ferreira, A., Tsui, S., Lacava, P. T., et al. (2012). Sugarcane growth promotion by the endophytic bacterium *Pantoea agglomerans* 33.1. *Appl. Environ. Microbiol.* 78, 7511–7518. doi: 10.1128/AEM.00836-12.

Ranganathan, A. K., Dela Peña, R. A., Malison, M. T., Punzalan, O. V., Pangilinan, C. R., and Gracilla, D. E. (2018). Cell-free supernatant from *Exiguobacterium acetylicum* isolated from water cabbage (*Pistia stratiotes*) roots inhibits *Bacillus subtilis* and *Escherichia coli*. *Pharmacogn. J.* 10, 198–201. doi: 10.5530/PJ.2018.1.34.

Rao, R. K., Vipin, A. V., Hariprasad, P., Anu Appaiah, K. A., and Venkateswaran, G. (2017). Biological detoxification of Aflatoxin B1 by *Bacillus licheniformis* CFR1. *Food Control* 71, 234–241. doi: 10.1016/J.FOODCONT.2016.06.040.

Ribaudo, C., Zaballa, J. I., and Golluscio, R. (2020). Effect of the phosphorus-solubilizing bacterium *Enterobacter ludwigii* on barley growth promotion. *Am. Acad. Sci. Res. J. Eng. Technol. Sci.* 63, 144–157. Available at: https://www.asrjetsjournal.org/index.php/American_Scientific_Journal/article/view/5484 [Accessed December 14, 2022].

Rijavec, T., Lapanje, A., Dermastia, M., and Rupnik, M. (2007). Isolation of bacterial endophytes from germinated maize kernels. *Can. J. Microbiol.* 53, 802–808. doi: 10.1139/W07-048/ASSET/IMAGES/LARGE/W07-048F1.JPEG.

Roca-Couso, R., David Flores-Félix, J., and Rivas, R. (2021). Mechanisms of action of microbial biocontrol agents against *Botrytis cinerea*. *J. Fungi* 7, 1045. doi: 10.3390/jof7121045.

Rojas-Solis, D., Ángel Vences-Guzmán, M., Sohlenkamp, C., and Santoyo, G. (2020). Antifungal and plant growth-promoting *Bacillus* under saline stress modify their membrane composition. *J. Soil Sci. Plant Nutr.* 20, 1549–1559. doi: 10.1007/s42729-020-00246-6.

Saad, M. M. G., Kandil, M., and Mohammed, Y. M. M. (2020). Isolation and identification of plant growth-promoting bacteria highly effective in suppressing root rot in fava beans. *Curr. Microbiol.* 77, 2155–2165. doi: 10.1007/S00284-020-02015-1/TABLES/6.

Saleh, A. E., Ul-Hassan, Z., Zeidan, R., Al-Shamary, N., Al-Yafei, T., Alnaimi, H., et al. (2021). Biocontrol activity of *Bacillus megaterium* BM344-1 against toxigenic fungi. *ACS Omega* 6, 10984–10990. doi: 10.1021/acsomega.1c00816.

Schwartz, A. R., Ortiz, I., Maymon, M., Herbold, C. W., Fujishige, N. A., Vijanderan, J. A., et al. (2013). Bacillus simplex—A little known PGPB with anti-fungal activity—alters pea legume root architecture and nodule morphology when coinoculated with *Rhizobium leguminosarum* bv. viciae. *Agronomy* 3, 595–620. doi: 10.3390/AGRONOMY3040595.

Selvakumar, G., Joshi, P., Nazim, S., Mishra, P. K., Kundu, S., and Gupta, H. S. (2009). *Exiguobacterium acetylicum* strain 1P (MTCC 8707) a novel bacterial antagonist from the north western Indian himalayas. *World J. Microbiol. Biotechnol.* 25, 131–137. doi: 10.1007/S11274-008-9874-4/TABLES/2.

Selvakumar, G., Joshi, P., Suyal, P., Mishra, P. K., Joshi, G. K., Bisht, J. K., et al. (2011). *Pseudomonas lurida* M2RH3 (MTCC 9245), a psychrotolerant bacterium from the Uttarakhand himalayas, solubilizes phosphate and promotes wheat seedling growth. *World J. Microbiol. Biotechnol.* 27, 1129–1135. doi: 10.1007/S11274-010-0559-4/TABLES/2.

Senger, M., Moresco, E., Dalbosco, M., Santin, R., Inderbitzin, P., and Barrocas, E. N. (2022). Methods to quantify *Bacillus simplex*-based inoculant and its effect as a seed treatment on field-grown corn and soybean in Brazil. *J. Seed Sci.* 44, 2022. doi: 10.1590/2317-1545V44263329.

Sheibani-Tezerji, R., Naveed, M., Jehl, M. A., Sessitsch, A., Rattei, T., and Mitter, B. (2015). The genomes of closely related *Pantoea ananatis* maize seed endophytes having different effects on the host plant differ in secretion system genes and mobile genetic elements. *Front. Microbiol.* 6, 440. doi: 10.3389/FMICB.2015.00440/ABSTRACT.

Shoebitz, M., Ribaudo, C. M., Pardo, M. A., Cantore, M. L., Ciampi, L., and Curá, J. A. (2009). Plant growth promoting properties of a strain of *Enterobacter ludwigii* isolated from *Lolium perenne* rhizosphere. *Soil Biol. Biochem.* 41, 1768–1774. doi: 10.1016/J.SOILBIO.2007.12.031.

Silva, H. S. A., Tozzi, J. P. L., Terrasan, C. R. F., and Bettiol, W. (2012). Endophytic microorganisms from coffee tissues as plant growth promoters and biocontrol agents of coffee leaf rust. *Biol. Control* 63, 62–67. doi: 10.1016/J.BIOCONTROL.2012.06.005.

Someya, N., Numata, S., Nakajima Akira Hasebe, M., Hibi, T., Akutsu, K., Someya, N., et al. (2003). Biological control of rice blast by the epiphytic bacterium *Erwinia ananas* transformed with a chitinolytic enzyme gene from an antagonistic bacterium, *Serratia marcescens* strain B2. *J. Gen. Plant Pathol.* 69, 276–282. doi: 10.1007/S10327-003-0043-1.

Sun, X., Ma, W., Xu, Y., Jin, X., and Ni, H. (2020). Complete genome sequence of *Rahnella aquatilis* MEM40, a plant growth-promoting rhizobacterium isolated from rice rhizosphere soil, with antagonism against *Magnaporthe oryzae* and *Fusarium graminearum*. *Microbiol. Resour. Announc.* 9, e00651-20. doi: 10.1128/MRA.00651-20.

Thijs, S., Van Dillewijn, P., Sillen, W., Truyens, S., Holtappels, M., D´Haen, J., et al. (2014). Exploring the rhizospheric and endophytic bacterial communities of *Acer pseudoplatanus* growing on a TNT-contaminated soil: towards the development of a rhizocompetent TNT-detoxifying plant growth promoting consortium. *Plant Soil* 385, 15–36. doi: 10.1007/S11104-014-2260-0/FIGURES/5.

Tontou, R., Giovanardi, D., Ferrari, M., and Stefani, E. (2016). Isolation of bacterial endophytes from *Actinidia chinensis* and preliminary studies on their possible use as antagonists against *Pseudomonas syringae* pv. *actinidiae*. *J. Berry Res.* 6, 395–406. doi: 10.3233/JBR-160118.

Vandana, U. K., Chopra, A., Choudhury, A., Adapa, D., and Mazumder, P. B. (2018). Genetic diversity and antagonistic activity of plant growth promoting bacteria, isolated from tea-rhizosphere: a culture dependent study. *Biomed. Res.* 29, 853–864. doi: 10.1007/s12088-014-0470-z.

Vazquez, P., Holguin, G., Puente, M. E., Lopez-Cortes, A., and Bashan, Y. (2000). Phosphate-solubilizing microorganisms associated with the rhizosphere of mangroves in a semiarid coastal lagoon. *Biol. Fertil. Soils 2000 305* 30, 460–468. doi: 10.1007/S003740050024.

Wang, J., Yang, M., Song, Y., Acevedo, F. E., Hoover, K., Zeng, R., et al. (2018). Gut-associated bacteria of *Helicoverpa zea* indirectly trigger plant defenses in maize. *J. Chem. Ecol.* 44, 690–699. doi: 10.1007/S10886-018-0970-0/TABLES/1.

Yanti, Y., Warnita, Reflin, and Busniah, M. (2018). Indigenous endophyte bacteria ability to control *Ralstonia* and *Fusarium* wilt disease on chili pepper. *Biodiversitas* 19, 1532–1538. doi: 10.13057/biodiv/d190446.

Yasin, N. A., Khan, W. U., Ahmad, S. R., Ali, A., Ahmad, A., and Akram, W. (2018). Imperative roles of halotolerant plant growth-promoting rhizobacteria and kinetin in improving salt tolerance and growth of black gram (*Phaseolus mungo*). *Environ. Sci. Pollut. Res.* 25, 4491–4505. doi: 10.1007/S11356-017-0761-0/TABLES/8.

Yun, N. R., Shin, Y. K., Hwang, S. Y., Kuraishi, H., Sugiyama, J., and Kawahara, K. (2000). Chemotaxonomic and phylogenetic analyses of *Sphingomonas* strains isolated from ears of plants in the family Gramineae and a proposal of *Sphingomonas roseoflava* sp. nov. *J. Gen. Appl. Microbiol.* 46, 9–18. doi: 10.2323/JGAM.46.9.

Zhao, Y., Yuan, Z., Wang, S., Wang, H., Chao, Y., Sederoff, R. R., et al. (2022). Gene sdaB is involved in the nematocidal activity of *Enterobacter ludwigii* AA4 against the pine wood nematode *Bursaphelenchus xylophilus*. *Front. Microbiol.* 13, 870519. doi: 10.3389/FMICB.2022.870519.

Zheng, L., Situ, J. jian, Zhu, Q. feng, Xi, P. gen, Zheng, Y., Liu, H. xia, et al. (2019). Identification of volatile organic compounds for the biocontrol of postharvest litchi fruit pathogen *Peronophythora litchii*. *Postharvest Biol. Technol.* 155, 37–46. doi: 10.1016/J.POSTHARVBIO.2019.05.009.

Zhou, J., Li, P., Meng, D., Gu, Y., Zheng, Z., Yin, H., et al. (2020). Isolation, characterization and inoculation of Cd tolerant rice endophytes and their impacts on rice under Cd contaminated environment. *Environ. Pollut.* 260, 113990. doi: 10.1016/J.ENVPOL.2020.113990.

Zinniel, D. K., Feng, Z., Blum, P. H., Barletta, R. G., and Vidaver, A. K. (2008). Plasmid transformation and expression of the firefly luciferase in *Microbacterium testaceum* type and endophytic colonizing field strains. *Can. J. Microbiol.* 54, 964–970. doi: 10.1139/W08-086.

Zul, D., Wanner, G., and Overmann, J. (2008). *Massilia brevitalea* sp. nov., a novel betaproteobacterium isolated from lysimeter soil. *Int. J. Syst. Evol. Microbiol.* 58, 1245–1251. doi: 10.1099/IJS.0.65473-0/CITE/REFWORKS.
