# Supplementary figures and images for: Maize pollen carry bacteria that suppress a fungal pathogen that enters through the male gamete fertilization route
Source: Front Plant Sci. 2024 Jan 10;14:1286199. doi: 10.3389/fpls.2023.1286199 (PMC10806238; doi:10.3389/fpls.2023.1286199)

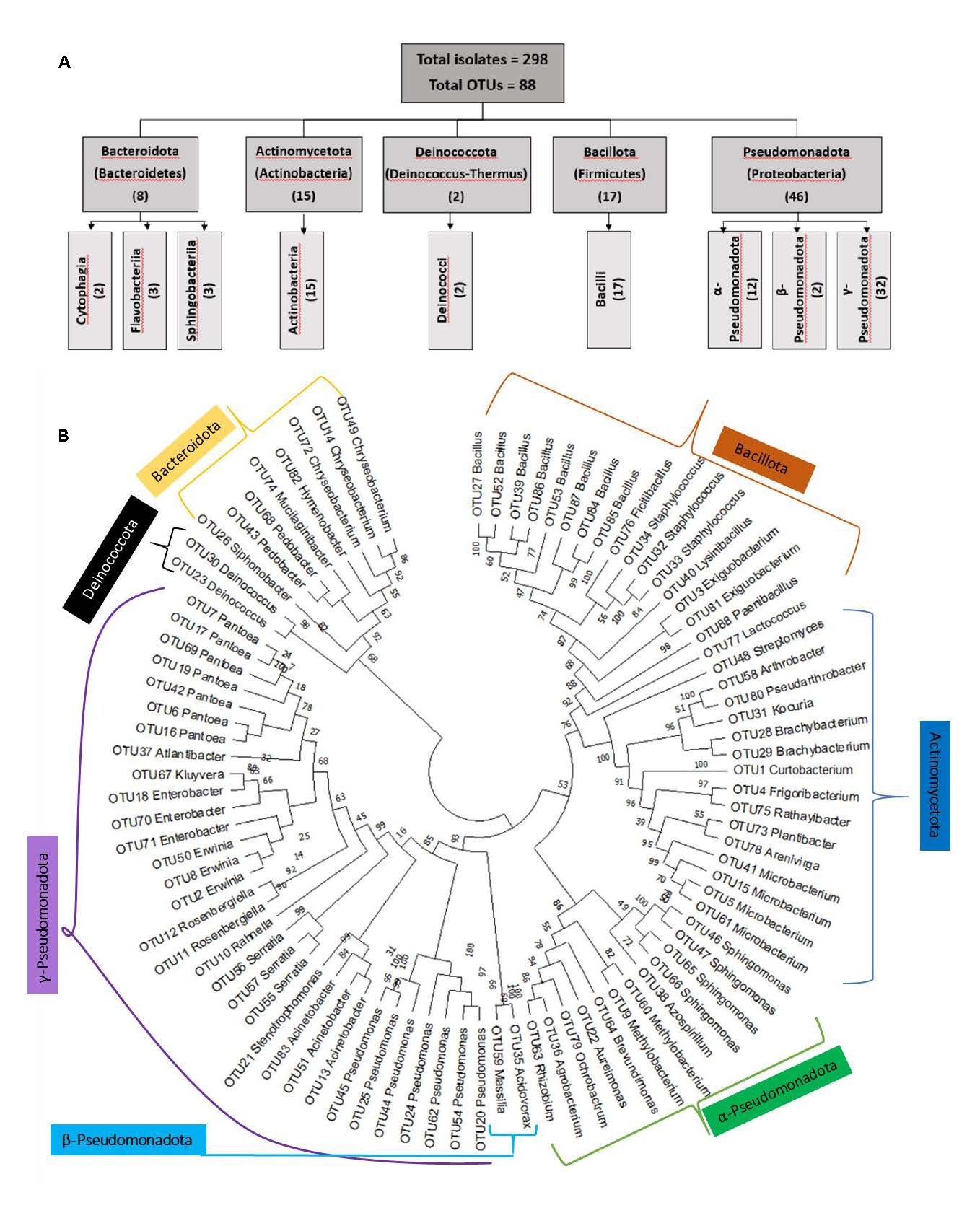

Supplement: Supplementary Figure 1 — Summary of the taxonomy of cultured bacteria from pollen of diverse American maize grown in a common field at the phylum, class, and OTU level. (A) Diagrammatic sketch of the taxonomies (full-length 16S RNA) of the pollen-associated bacteria based on phylum and class. (B) Maximum likelihood (ML) phylogenetic tree of pollen-associated bacteria cultured from different host maize accessions based on unique operational taxonomic units (OTUs). Bootstrap values are indicated above the branches. [file DataSheet_1.zip › Supplementary Figure S1.JPEG]

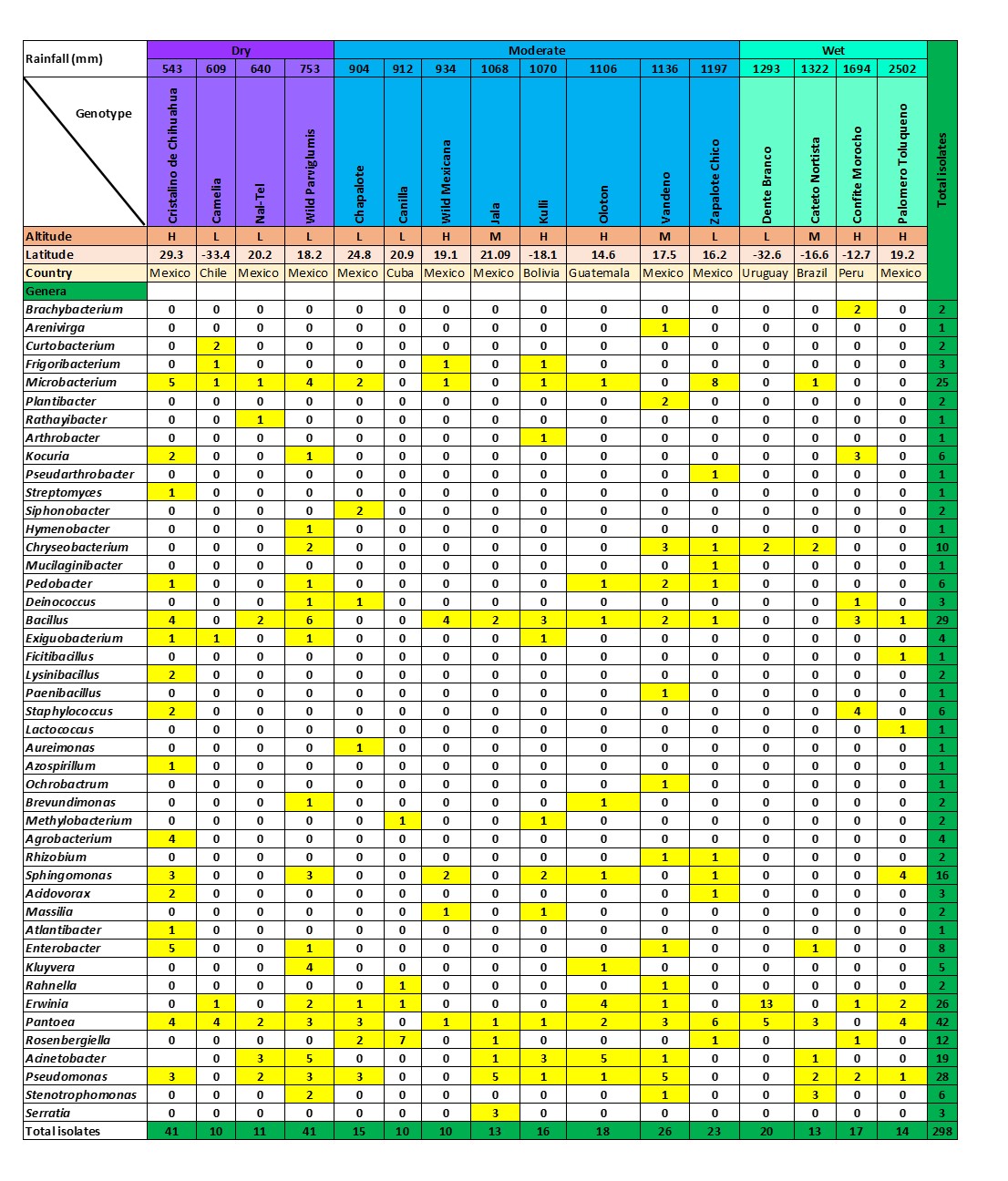

Supplement: Supplementary Figure 1 — Summary of the taxonomy of cultured bacteria from pollen of diverse American maize grown in a common field at the phylum, class, and OTU level. (A) Diagrammatic sketch of the taxonomies (full-length 16S RNA) of the pollen-associated bacteria based on phylum and class. (B) Maximum likelihood (ML) phylogenetic tree of pollen-associated bacteria cultured from different host maize accessions based on unique operational taxonomic units (OTUs). Bootstrap values are indicated above the branches. [file DataSheet_1.zip › Supplementary Figure S2.JPEG]

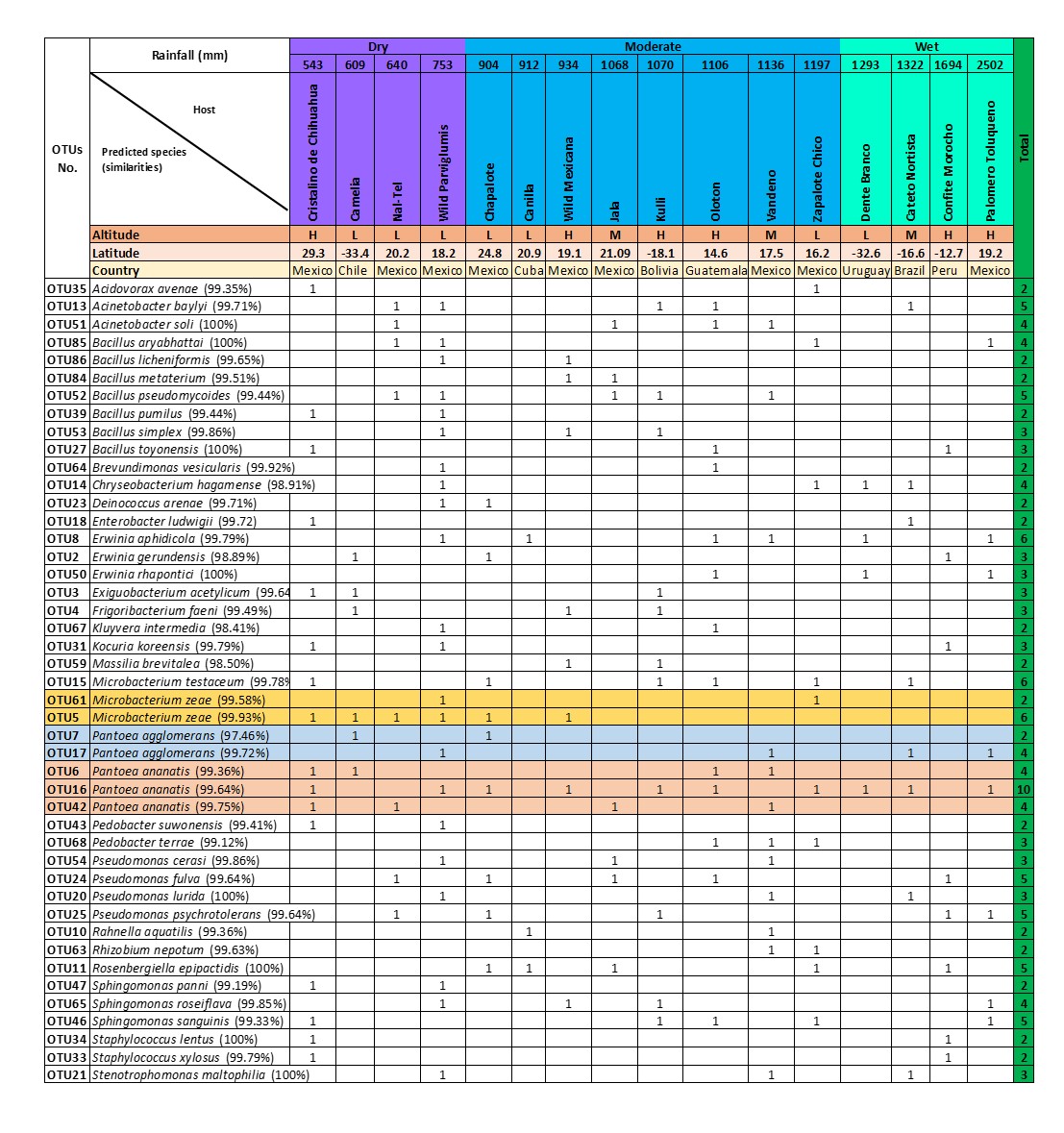

Supplement: Supplementary Figure 1 — Summary of the taxonomy of cultured bacteria from pollen of diverse American maize grown in a common field at the phylum, class, and OTU level. (A) Diagrammatic sketch of the taxonomies (full-length 16S RNA) of the pollen-associated bacteria based on phylum and class. (B) Maximum likelihood (ML) phylogenetic tree of pollen-associated bacteria cultured from different host maize accessions based on unique operational taxonomic units (OTUs). Bootstrap values are indicated above the branches. [file DataSheet_1.zip › Supplementary Figure S3.JPEG]

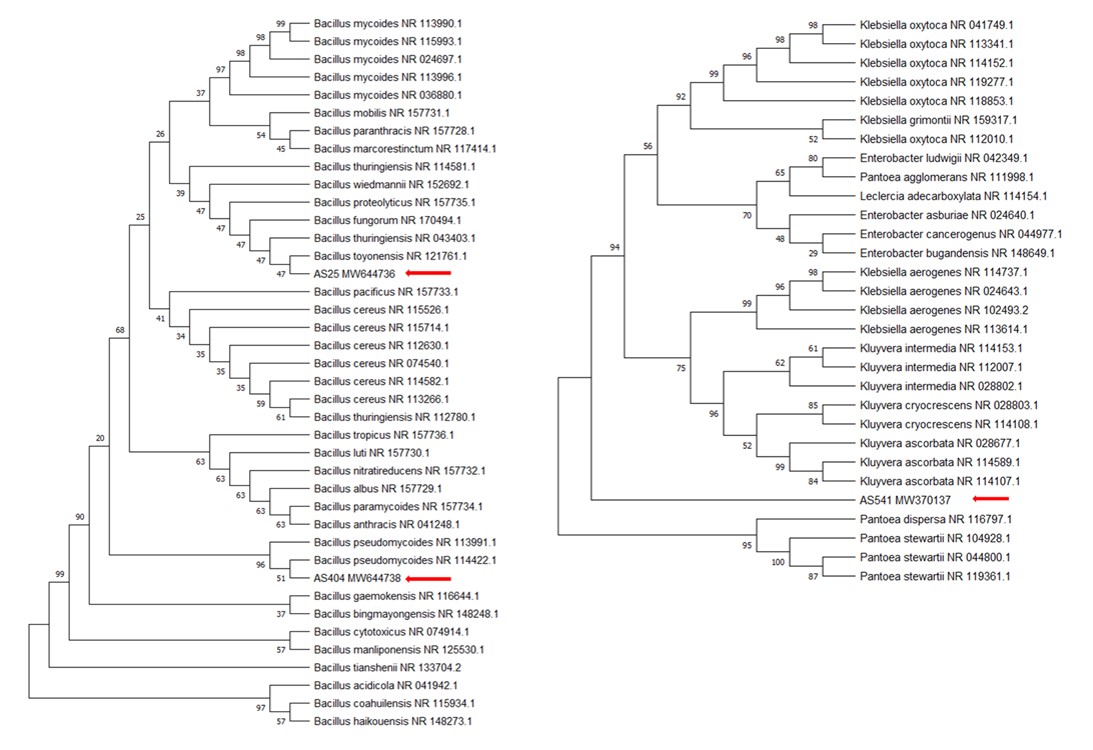

Supplement: Supplementary Figure 1 — Summary of the taxonomy of cultured bacteria from pollen of diverse American maize grown in a common field at the phylum, class, and OTU level. (A) Diagrammatic sketch of the taxonomies (full-length 16S RNA) of the pollen-associated bacteria based on phylum and class. (B) Maximum likelihood (ML) phylogenetic tree of pollen-associated bacteria cultured from different host maize accessions based on unique operational taxonomic units (OTUs). Bootstrap values are indicated above the branches. [file DataSheet_1.zip › Supplementary Figure S4.JPEG]

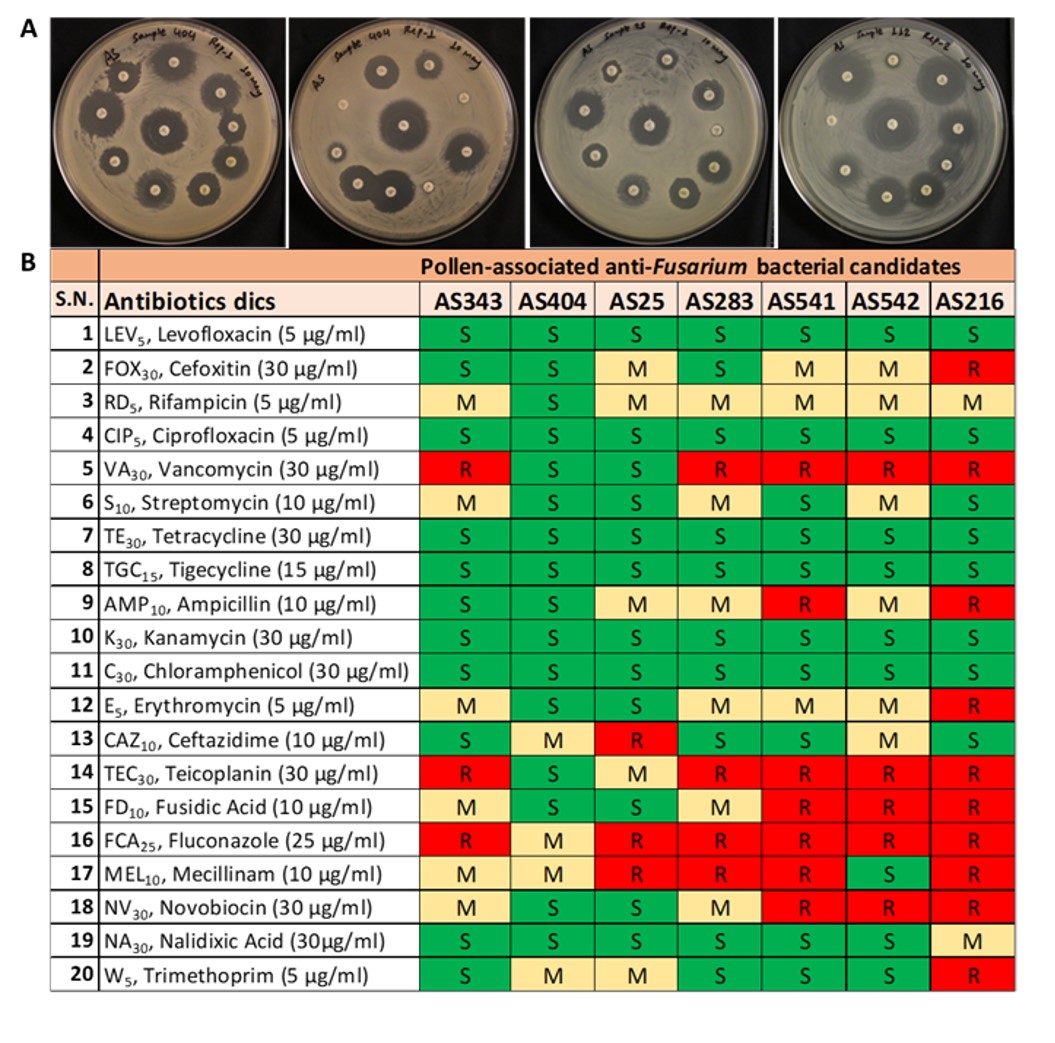

Supplement: Supplementary Figure 1 — Summary of the taxonomy of cultured bacteria from pollen of diverse American maize grown in a common field at the phylum, class, and OTU level. (A) Diagrammatic sketch of the taxonomies (full-length 16S RNA) of the pollen-associated bacteria based on phylum and class. (B) Maximum likelihood (ML) phylogenetic tree of pollen-associated bacteria cultured from different host maize accessions based on unique operational taxonomic units (OTUs). Bootstrap values are indicated above the branches. [file DataSheet_1.zip › Supplementary Figure S5.JPEG]
